# Acylated Flavonoid Glycosides are the Main Pigments that Determine the Flower Colour of the Brazilian Native Tree *Tibouchina pulchra* (Cham.) Cogn.

**DOI:** 10.3390/molecules24040718

**Published:** 2019-02-16

**Authors:** Fernanda Mendes Rezende, Marcelo José Pena Ferreira, Mads Hartvig Clausen, Magdalena Rossi, Claudia Maria Furlan

**Affiliations:** 1 Botany Department, Institute of Bioscience, University of São Paulo (USP), São Paulo 05508-060, Brazil; marcelopena@ib.usp.br (M.J.P.F.); magdarossirosso@gmail.com (M.R.); 2 Center for Nanomedicine and Theranostics, Department of Chemistry, Technical University of Denmark, Kgs. 2800 Lyngby, Denmark; mhc@kemi.dtu.dk

**Keywords:** flavonol, kaempferol, anthocyanin, Melastomataceae

## Abstract

*Tibouchina pulchra* (Cham.) Cogn. is a plant native to Brazil whose genus and family (Melastomataceae) are poorly studied with regards to its metabolite profile. Phenolic pigments of pink flowers were studied by ultra-performance liquid chromatography with a photodiode array detector and electrospray ionization quadrupole time-of-flight mass spectrometry. Therein, twenty-three flavonoids were identified with eight flavonols isolated by preparative high-performance liquid chromatography and analysed by one- and two-dimensional nuclear magnetic resonance. Kaempferol derivatives were the main flavonols, encompassing almost half of the detected compounds with different substitution patterns, such as glucoside, pentosides, galloyl-glucoside, *p*-coumaroyl-glucoside, and glucuronide. Concerning the anthocyanins, petunidin *p*-coumaroyl-hexoside acetylpentoside and malvidin *p*-coumaroyl-hexoside acetylpentoside were identified and agreed with previous reports on acylated anthocyanins from Melastomataceae. A new kaempferol glucoside was identified as kaempferol-(2′′-*O*-methyl)-4′-*O*-α-d-glucopyranoside. Moreover, twelve compounds were described for the first time in the genus with five being new to the family, contributing to the chemical characterisation of these taxa.

## 1. Introduction

*Tibouchina* Aubl., the most representative genus within Melastomataceae, has approximately 460 species [1,2,3]. Melastomataceae can be recognised among eudicots by the characteristic leaf acrodromous venation pattern [4]. The family is the fifth largest group among Angiosperms in Brazil [5], comprising 4500 species and approximately 170 genera. In spite of pantropical distribution, the greatest diversity of species is found in the Neotropics (ca. 3000 species), with 929 species native to Brazil [6]. Out of the 166 *Tibouchina* species reported in Brazil, 105 are endemic [6], occurring mainly in the Atlantic Rainforest and in the Cerrado (Brazilian savannahs); both biomes are recognised as biodiversity hotspots [7]. This native vegetation is constantly under illegal deforestation and agribusiness expansion, generating a need for programmes for biodiversity conservation and, consciously, resources exploitation.

*Tibouchina* species occur in open areas, such as forest edges and clearings, and are considered important for restoration/reforestation purposes [8]. Moreover, *Tibouchina granulosa* (Desr.) Cogn. and *Tibouchina pulchra* (Cham.) Cogn. have been characterised as possible biomonitors of air pollution, such as particulate matter and ozone [9,10,11,12,13,14,15,16]. Despite the ecological importance of this genus, the main use of *Tibouchina* is urban ornamentation and nowadays, several cultivars are available in the flower market. A large contributor to the beauty and fascinating feature of *T. pulchra* is the colour change of the flowers from white to intense pink during development [17].

Few examples of traditional uses are described in the literature for *Tibouchina*. Among them are anti-inflammatory, antioxidant [18], antinociceptive (relieving chronic pain) [19], antibacterial [20,21], antifungal [22,23], antiparasitic [20,24,25], and anticancer [26] activities of leaf extracts. Nonetheless, the chemical composition of *Tibouchina* largely remains elusive, with only eleven species being characterised phytochemically. These few reports described the presence of several natural products, such as flavonoids (flavonol glycosides, isoflavonoids and anthocyanins), phenolic derivatives, tannins, and triterpenes in distinct organs of the plant. Structural elucidation by nuclear magnetic resonance (NMR) was performed only for some triterpenes, tannins, flavonols and anthocyanins in *T. urvilleana* (DC.) Cogn. and *T. lepidota* (Bonpl.) Baill. [27,28,29] (Table 1).

Due to the importance of *Tibouchina* species for ornamentation and ecological purposes, the present work aimed to assess the qualitative profile of acidified alcoholic extract from *T. pulchra* flowers. An ultra-performance liquid chromatography with photodiode array detector and electrospray ionization quadrupole time-of-flight mass spectrometry (UPLC-PAD-ESI-QTOF-MS) method was established, and thirty-two compounds were detected with twenty-three identified, many of them reported for the first time in the species, genus, and family, as well as a new flavonol: kaempferol-(2′′-*O*-methyl)-4′-*O*-α-d-glucopyranoside.

## 2. Results and Discussion

### 2.1. Chemical Screening of *Tibouchina pulchra* Flowers

**Figure 1:**
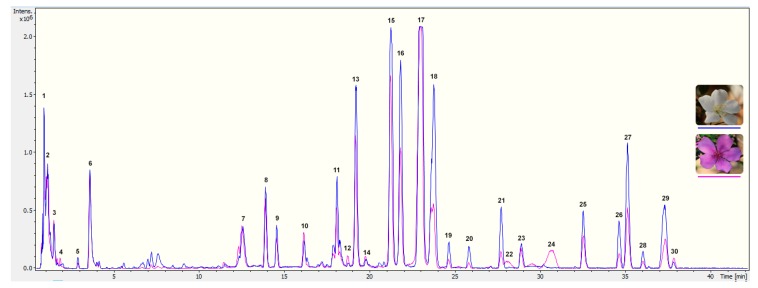
Chromatogram obtained by UPLC-PDA-ESI-QTOF-MS from *T. pulchra* petals extracted with acidified methanol. Chromatographic separation was performed with a column Waters Acquity UPLC C18 (1.7 µm, 100 × 2.1 mm) at a flow rate of 0.3 mL min^−1^, using 4 µL of injection volume, column temperature of 45°C and a solvent system composing 1% formic acid in water (A) and 1% formic acid in acetonitrile (B). Gradient elution were as follow: 5 to 25% of B (0–40 min), 25 to 100% of B (40–42 min), 100% of B (42.0–42.5 min), 100 to 5% of B (42.5–43.0 min) and 5% of B (43–46 min). MS scans were performed in positive ion mode (MS+) in the range *m*/*z* 75−1,250, and in the following conditions: capillary voltage set to 4,500 V, end plate offset at −500 V, nebulizer at 2 Bar, dry gas at 12 L min^−1^ and dry gas temperature at 200°C. MS was calibrated using sodium formate. All data were processed using Data analysis software 4.2 (Bruker). Numbers correspond to the identification presented in Table 2.

To explore the pigment profiling of *T. pulchra* petals, the first step was to analyse acidic alcoholic extracts from white and pink flowers by UPLC-PAD-ESI-QTOF-MS. The exclusive difference between the two floral stages was the presence of anthocyanins in pink flower extracts (Figure 1). In order to perform a complete characterisation of floral pigments, the pink floral stage was chosen for isolation and identification of constituents.

In the chromatograms shown in Figure 1, two classes of phenolics were found: phenolic acids (including cinnamic derivatives, constituents **1** to **6**) and flavonoids (flavonols and anthocyanins, constituents **7** to **30**). Based on analysis of the MS data (Figure S1), the presence of thirty-two compounds is suggested (Table 2), due to the co-elution of some compounds in the chromatographic analysis.

The main flavonols identified in the petal extract were kaempferol, quercetin, and myricetin (Table 2 and Table 3), which were previously described in leaves of *T. pulchra* but only with hexosyl and pentosyl substituents [10,36]. The most abundant flavonol skeleton was kaempferol (*m*/*z* 287.0548) with different substituents as glucuronyl methyl ester (constituent **23**), galloylhexosides (constituents **13**, **16** and **19**), and *p*-coumaroylhexosides (**21**, **27**, **28** and **30**). Quercetin derivatives (*m*/*z* 303.0496, constituents **10**, **11**, **12** and **25**) were the second most abundant flavonol identified, followed by myricetin derivatives (*m*/*z* 319.0445, constituents **8** and **9**).

Compounds **8**, **9**, **11**, **15** and **17** showed the loss of 162 amu during MS analysis, indicating the presence of a hexose, probably a galactosyl or a glucosyl group (Figure S1). Pentoses, as arabinose, apiose or xylose, were also found as substituents in the case of compounds **18** and **20**, which exhibited a mass loss of 132 amu. Compounds **12** and **23** showed hexauronic acids as substituents, identified by the mass loss of 176 and 190 amu, respectively. The difference is presumably the methyl group in compound **23** as consequence of solvent artefact during extraction procedures. Compound **23** was analysed by NMR and confirmed as glucuronic acid methyl ester substituent. Literature describes glucose as the most commonly identified sugar in flavonoids, while galactose, rhamnose, xylose, and arabinose are less frequent. Yet, mannose, fructose, glucuronic, and galacturonic acids are rare [41,42,43].

Mass loss of 314 amu indicates the presence of galloylhexoside. This group was identified in compound **10**, a quercetin derivative, and in the isomers **13**, **16** and **19**, which are kaempferol derivatives. The fragment *m*/*z* 153.0181 was intense for these compounds, which can be ascribed to a galloyl substituent and to a typical ion signal from a fragment of A-ring+ [41], generated by retro Diels–Alder fragmentation of the C-ring (Table 3). The mass spectrum of compound **19** showed the fragment *m*/*z* 449.1071, a loss of 152 amu from *m*/*z* 601.1170 [M + H]^+^, corroborating the galloyl substitution [41,44,45]. Moreover, the additional mass loss of 162 amu confirmed the presence of hexoside (glucoside or galactoside).

Regarding the *p*-coumaroyl group, we identified eight compounds with this acylation pattern: **21**, **22**, **24**, **25**, **26**, **27**, **28**, and **30**. A mass loss of 308 amu is indicative of *p*-coumaroylhexose substitution, but it can also indicate a rutinosyl group (6-rhamnosylglucose) as substituent. The fragment of *m*/*z* 147.0439 found in compounds **25**, **27**, **28**, and **30** confirmed the presence of either a *p*-coumaroyl or a rhamnosyl substituent. Furthermore, acylation with hydroxycinnamic acids, as *p*-coumaric acid, shifts the band I from ultraviolet/visible (UV/VIS) spectra of the flavonols to a lower wavelength, resulting in a peak or shoulder at 305–310 nm. In addition, acylation of the sugar moiety also increases retention time in a chromatographic analysis [46,47,48], as shown in Table 2.

Concerning anthocyanins, malvidin and petunidin were the aglycones identified (Table 2 and Table 4). Petunidin *p*-coumaroylhexoside acetylpentoside (**22**) showed a molecular ion of *m*/*z* 799.2077 [M + H]^+^, in which fragmentation resulted in *m*/*z* 625.1552 [M − 174]^+^, *m*/*z* 491.1176 [M − 308]^+^ and *m*/*z* 317.0665 [M − 482]^+^, corresponding to the neutral loss of acetylpentoside and *p*-coumaroylhexoside from a petunidin. The other anthocyanin, malvidin *p*-coumaroylhexoside acetylpentoside (**24**), exhibited a fragment of *m*/*z* 813.2243 [M + H]^+^, whose fragmentation resulted in *m*/*z* 639.1716 [M − 174]^+^, *m*/*z* 505.1336 [M − 308]^+^ and *m*/*z* 331.0812 [M − 482]^+^ consistent with the loss of acetylpentoside and *p*-coumaroylhexoside from malvidin (Figure S1). Although previously reported in *T. lepdota* and *T. urvilleana* [27,29], acylated anthocyanins were identified here for the first time in *T. pulchra*.

The chemical composition of polar extracts of *T. pulchra* revealed thirty-two compounds, with twenty-three identified by UV/VIS and MS. Nuclear magnetic resonance spectroscopy was used as additional technique to support the structural elucidation of eight compounds.

### 2.2. Structural Elucidation of Acylated Flavonoids by NMR and Identification of a New Flavonol Glucoside

Flavonoid acylation can influence the biological activity of compounds by altering their solubility, stability, reactivity, and interaction with cellular targets [49], and with regards to the colour of flowers, esterification typically enhances the intensity [50]. Thus, we further isolated the acylated flavonoids by preparative high-performance liquid chromatography (HPLC) to investigate their structure through NMR spectroscopy. Successful isolation was achieved for kaempferol 3-*O*-(2′′-*O*-galloyl)-β-d-glucopyranoside (**13**, **16** or **19**; Figures S2–S8 and Table S1), kaempferol 3-*O*-(6′′-*O*-galloyl)-β-d-glucopyranoside (**13**, **16** or **19**; Figures S9–S15 and Table S1), kaempferol 3-*O*-glucuronide-6′′-*O*-methylester (**23**; Figures S22–S26 and Table S2), quercetin 3-*O*-(6′′-*O*-*p*-coumaroyl)-β-d-glucopyranoside (**25**; Figures S27–S31 and Table S3), kaempferol 3-*O*-(6′′-*O*-*p*-coumaroyl)-β-d-glucopyranoside (**27**; Figures S32–S38 and Table S3), kaempferol (**29**; Figures S39–S41 and Table S4), and a mixture of kaempferol 3-*O*-β-d-glucopyranoside and kaempferol-(2′′-*O*-methyl)-4′-*O*-α-d-glucopyranoside (**17**; Table 5, Figure 2 and Figure S16–S21), which furnished a new compound.

**Figure 2:**
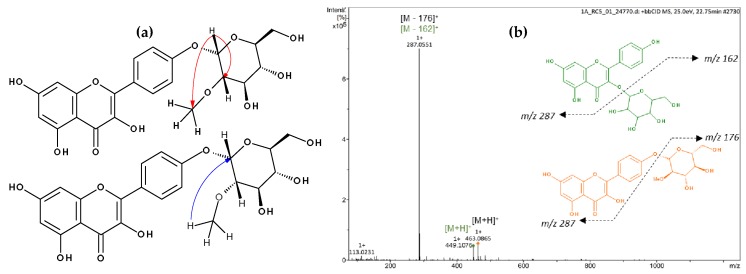
Spectrometric analyses of mixture **17**. (**a**) Principal HMBC correlations of kaempferol-(2′′-*O*-methyl)-4′-*O*-*α*-d-glucopyranoside. (**b**) Mass Spectrum and main fragmentation.

The mass spectrum of **17** was indicative of a mixture of two compounds, which differed from each other in 14 amu, suggesting the presence of an additional methyl group in one of the structures (Figure 2). In the NMR spectrum of **17**, seven signals typical of aromatic hydrogens were observed: three doublets at δ 6.22 (1H, d, *J* = 2.0 Hz, H6), δ 6.21 (1H, d, *J* = 2.0 Hz, H6), and δ 6.46 (2H, s, H8), corresponding to a *meta*-coupling of these protons which were attributed to the flavonoid A-ring; and another three doublets with *ortho*-coupling constants at δ 6.89 (2H, d, *J* = 8.4 Hz, H3′ and 5′), δ 6.93 (2H, d, *J* = 8.5 Hz, H3′ and 5′) and δ 8.04 (4H, d, *J* = 8.4 Hz, H2′ and 6′), suggesting two *para*-substituted B-rings of flavonoids. The anomeric protons appeared at δ 5.46 (1H, d, *J* = 7.6 Hz, H1′′) and δ 4.51 (1H, d, *J* = 3.6 Hz, H1′′). The smaller coupling constant of the latter suggests an α-linked carbohydrate. Signals between δ 3.09 and δ 3.58 were attributed to the hydrogens of the sugar moiety. The presence of a methyl group was confirmed by signals at δ 3.26 (s, 3H) and δ 54.74 (*O*Me). Furthermore, the heteronuclear multiple bond correlation (HMBC) spectrum showed two relevant correlations: the first between the anomeric protons at δ 5.46 with C3 (δ 133.63) from the flavonoid C-ring, confirming the position of the sugar moiety in kaempferol 3-*O*-β-d-glucopyranoside (astragalin), and the second one, the anomeric proton (δ 4.51) with the methyl group at δ 54.74, suggesting the presence of 2-methoxyglycosyl moiety (Figure 2). Although the correlation between the anomeric proton and carbon C4′ of the flavonoid was not observed, the position of the glycoside was supported by the ^13^C NMR chemical shifts at C2 (δ 147.27) and C3 (δ 136.10) positions of the flavonoid, which is consistent with the presence of a free hydroxyl group at C3 [51].

Astragalin is a common flavonol present in red wine and in many plants [52]. Flavonols are usually substituted at positions 3 and 7 [53]. The 4′ moiety is unusual but kaempferol 4′-*O*-β-d-glucopyranoside has already been described [54]. However, to the best of our knowledge, the 2′′ methylated, α-linked sugar in the 4′ position of the kaempferol-(2′′-*O*-methyl)-4′-*O*-α-d-glucopyranoside has not previously been reported in the literature.

### 2.3. Flavonoids in *Tibouchina* and Melastomataceae

In this work, seventeen compounds were described for the first time in *T. pulchra*, with twelve described for the first time in *Tibouchina*. Flavonols, especially myricetin derivatives, are characteristic within Mytales [4]. In *Tibouchina*, the most common flavonols are quercetin and isorhamnetin and in *T. pulchra*, kaempferol was the main flavonol. Moreover, kaempferol derivatives have only been described before in *T. ciliaris* and *T. pereirae*. Regarding anthocyanins, malvidin has already been identified in *T. lepidota*, *T. grandiflora*, *T. semidecantra*, and *T. urvelleana*, while petunidin has been described exclusively in *T. granulosa* (Table 1). Although the acylation of anthocyanins has already been reported for the Melastomatoideae [4], this is the first characterisation of anthocyanins (i.e., malvidin and petunidin derivatives) in *T. pulchra*.

The most common acyl groups generally found as flavonoid substituents are hydroxycinnamic acids (e.g., caffeic, ferulic and *p*-coumaric acids) [55]. Flavonoids with a *p*-coumaroyl group have already been described for *Tibouchina*, such as *T. ciliaris* (kaempferol 7-*O*-*p*-coumaroyl), *T. grandiflora* (malvidin 3-(*p*-coumaroyl)-sambubioside-5-glucoside), malvidin 3-(*p*-coumaroyl-glucoside)-5-glucoside), and *T. urvilleana* (malvidin 3-*O*-(6-*O*-*p*-coumaryl-β-d-glucopyranoside)-5-*O*-(2-*O*-acetyl-β-d-xylopyranoside)) [27,30,31,40], which are in agreement with the results obtained for *T. pulchra*.

Acylation with hydroxybenzoic acids, such as gallic acid, is rare in angiosperms because the active production of hydrolysable tannins is restricted to certain orders as Alismatales, Cornales, Dilleniales, Ericales, Fagales, Geraniales, Juglandales, Myrtales, Proteales, Rosales, Sapindales, and Saxifragales [56]. Tannin occurrence has been described in at least three species of *Tibouchina*: *T. semidecantra*, *T. pulchra* and *T. multiflora* [10,12,34,36,38] (Table 1). Although hydrolysable tannins were neither identified in *T. ciliaris* nor in *T. granulosa*, the presence of quercetin 6′′-*O*-gallate and quercetin 3-(*O*-galloyl)-hexoside, respectively [31,34], is indicative of the existence of this class of metabolites. Here, we found quercetin galloylhexoside and three isomers of kaempferol galloylhexoside (**13**, **16** and **19**), with two of them successfully isolated for NMR analysis (kaempferol-3-*O*-(6′′-*O*-galloyl)-β-d-glucopyranoside and kaempferol-3-*O*-(2′′-*O*-galloyl)-β-d-glucopyranoside) and one not isolated. This substitution pattern agrees with previous findings of tannins in *T. pulchra* [10,12,36].

Although in Melastomataceae, many compounds have been isolated and identified by extensive spectrometric analyses. However, considering the size of the family, the number of studied species is still low. The most commonly found natural products in this family are terpenes, simple phenolics, quinones, lignans and flavonoids, as well as a vast range of tannins, mainly hydrolysable ones [57].

The results obtained for *T. pulchra* in the present study describe, for the first time, the presence of five compounds in this family: kaempferol 3-*O*-(6′′-*O*-galloyl)-β-d-glucopyranoside (**13**, **16** or **19**), kaempferol 3-*O*-(2′′-*O*-galloyl)-β-d-glucopyranoside (**13**, **16** or **19**), kaempferol-(2′′-*O*-methyl)-4′-*O*-α-d-glucopyranoside (**17**), kaempferol 3-*O*-glucuronide-6′′-*O*-methylester (**23**) and kaempferol 3-*O*-(6′′-*O*-*p*-coumaroyl)- β-d-glucopyranoside (**27**). Serna and Martinéz (2015) reviewed the chemical characterisation of Melastomataceae by considering only constituents identified by NMR. Kaempferol 3-*O* substitute was found only in *Miconia cabucu* Hoehne and *M. rubiginosa* (Bonpl.) DC., but usually kaempferol is 7-*O* substituted in this family [58]. Kaempferol aglycone (**29**) was also found in *Medinilla magnifica* Lindley and *Centradenia floribunda* Planch [57]. Regarding anthocyanins, malvidin *p*-coumaroylhexoside acetylpentoside (**24**) was the major anthocyanin in *T. pulchra*, which agrees with the proposition of malvidin as the most common anthocyanin nucleus in Melastomataceae [57]. This was the first description of petunidin *p*-coumaroylhexoside acetylpentoside (**22**) in *T. pulchra* and in Melastomataceae, since previous studies had described pelargonidin, cyanidin, peonidin, delphinidin, and malvidin glycosides or acylglycosides [57] in this species and family. Further NMR studies of *T. pulchra* anthocyanins are necessary to underpin the identification performed here by MS and UV.

The large number of flavonols identified in the pink stage of *T. pulchra* flowers might be an effect of co-pigmentation. It is known that this class of substances is related with white colour and co-pigmentation in coloured tissues. Co-pigmentation can be defined as the formation of noncovalent complexes involving an anthocyanin or anthocyanin-derived pigment and a co-pigment (in the presence or absence of metal ions), as well as subsequent changes in optical properties of the pigment. There are over ten thousand compounds of different classes of phenolic compounds (e.g., hydrolysable tannins, flavonoids, and phenolic acids) that help to stabilise the colour of flowers and increase colour intensity. In addition, glycosylation and acylation enhance the brightness of anthocyanin colours [50].

In conclusion, the Melastomataceae, and in particular, *Tibouchina* taxa are poorly characterised chemically. Here, out of the seventeen compounds described for the first time in *T. pulchra*, five of them are reported in the family: **13**, **16**, and **19** (we isolated two isomers); **17** (only kaempferol-(2′′-*O*-methyl)-4′-*O*-α-d-glucopyranoside); **23**; and **27**. Moreover, a novel flavonol was identified as kaempferol 4′-*O*-(2′′-methyl)-α-d-glucopyranoside. Recent advances in spectrometric techniques offer a unique opportunity to improve our knowledge about the chemical structure of natural products. Studies about flower anthocyanins are scarce, and the understanding of their structure, biosynthesis, and the regulatory mechanisms involved in their accumulation pattern helps to improve our knowledge about plant secondary metabolism—as well as the relationship between flower colour and the attraction of pollinators—and brings new insights for future biotechnological applications.

## 3. Materials and Methods

### 3.1. Plant Material

A pool of white and pink petals of *Tibouchina pulchra* were sampled from five different plants at Praça Carlos José Gíglio, São Paulo (Latitude: −23.57998, Longitude: −46.73403) in the most vigorous flowering period (May and June 2016) between 08:00 and 09:00. Petals were immediately frozen in liquid nitrogen and stored at −80 °C until processing. Freeze-dried (K202, Liobras, São Carlos, Brazil) samples were crushed in a ball mill for further analyses. A voucher (ID: Furlan73) was deposited in the Herbarium of the University of São Paulo.

### 3.2. Extraction and Analysis by UPLC-PAD-ESI-QTOF-MS

Phenolic compounds were extracted from 100 mg of petal powder twice with 1.5 mL of 0.2% hydrochloric acid (HCl) in methanol (MeOH). The samples were sonicated for 10 min and centrifuged at 10,000 rpm for 10 min. The extract was filtered (0.45 μm) and analysed by UPLC-PAD-ESI-QTOF-MS. The MS/MS analysis was performed with a Broadband Collision Induced Dissociation (bbCID) detector (Bruker, Bremen, Germany). Separation was achieved by using a C18 column at a flow rate of 0.3 mL min^−1^ and 4 µL of injection volume. The column temperature was 45 °C, and the solvent system was composed of 1% formic acid in water (A) and 1% formic acid in acetonitrile (B). Gradient elutions were as follow: 5 to 25% of B (0–40 min), 25 to 100% of B (40–42 min), 100% of B (42.0–42.5 min), 100 to 5% of B (42.5–43.0 min), and 5% of B (43–46 min). Separated compounds were first monitored using a photodiode array detector (PAD) (200 to 600 nm), and then MS scans were performed in positive ion mode (MS^+^) in the range *m*/*z* 75–1250, under the following conditions: capillary voltage set to 4500 V, end plate offset at −500 V, nebulizer at 2 Bar, dry gas flow of 12 L min^−1^ and dry gas temperature at 200 °C. The MS signal was calibrated using sodium formate. All data were processed using data analysis.

### 3.3. Isolation by Preparative HPLC and Identification by NMR

Acylated flavonoids were isolated from 10 g of pink petal powder by extracting four times with 200 mL of 0.2% HCl in MeOH. Samples were sonicated for 15 min, pillowed for 10 min, and vacuum filtered and concentrated using a rotary evaporator. The crude extract was diluted to approximately 250 mg/mL and analysed by preparative HPLC with a PAD. Separation was achieved on a C18 column at a flow rate of 20 mL min^−1^ using 1 mL of injection volume and a solvent system composed of 1% formic acid in water (A) and 1% formic acid in acetonitrile (B). Gradient elution were as follow: 10% of B (0–3 min), 10 to 15% of B (3–30 min), 15% of B (30–50 min), 15 to 20% of B (50–60 min), 20 to 25% of B (60–80 min), 25 to 35% of B (80–90 min), 35 to 45% of B (90–95 min), 45 to 100% of B (95–96 min), 100% of B (96–98 min), 100 to 10% of B (98.0–98.5 min), 10% of B (98.5–102.0 min) and monitored using PAD (200 to 600 nm). All the fractions were concentrated using a rotary evaporator. An aliquot was resuspended in 0.2% HCl in MeOH to check the purity by UPLC-MS. For the isolated compounds, the dried sample was dissolved in deuterated dimethyl sulfoxide (DMSO-d_6_) for NMR analysis. ^1^H and ^13^C NMR spectra were obtained using an AVANCE III HD spectrometer operating at frequency of 800.182 and 201.2 MHz, respectively, and equipped with a 5 mm TCI CryoProbe. Analyses of HMBC and heteronuclear single quantum coherence (HSQC) were also performed. All data were processed using MestreNova.

### 3.4. NMR Description

Kaempferol 3-*O*-(6′′-*O*-galloyl)-β-d-glucoside (**13**, **16** or **19**) appears as pale-yellow amorphous powder (yield of 0.6 mg). UV λ_max_ = 266, 290, 350 nm. [M + H]^+^ *m*/*z* 601.1183. ^1^H NMR (800 MHz, DMSO-d6): δH 12.52 (1H, s, OH-C5), 10.87 (1H, s, OH-C7), 10.06 (1H, s, OH-C4′), 7.94 (2H, d, *J* = 8.8 Hz, H-2′, H-6′), 6.92 (2H, s, H-2′′′, H-6′′′), 6.77 (2H, d, *J* = 8.8 Hz, H-3′, H-5′), 6.45 (1H, d, *J* = 2.0 Hz, H-8), 6.21 (1H, d, *J* = 2.0 Hz, H-6), 5.45 (1H, d, *J* = 7.6 Hz, H-1′′), 4.28 (1H, dd, *J* = 2.1, 11.8 Hz, H-6′′b), 4.17 (1H, dd, *J* = 12.0, 3.8 Hz, H-6′′a), 3.51 -3.48 (4H, m, H-2′′- H-5′′). ^13^C NMR (200 MHz, DMSO-d6): δC 165.36 (C-7′′′), 164.50 (C-7), 161.90 (C-5), 157.26 (C-9), 157.15 (C-2), 150.58 (C-4′); 146.11 (C-3′′′, C-5′′′), 138.94 (C-4′′′), 133.52 (C-3), 131.19 (C-2′, C-6′), 121.04 (C-1′), 120.00 (C-1′′′), 116.46 (C-3′, C-5′), 109.70 (C-2′′′, C-6′′′), 104.66 (C-10), 102.17 (C-1′′), 99.58 (C-6), 94.91 (C-8), 76.51 (C-3′′), 74.60 (C-5′′), 74.52 (C-2′′), 69.89 (C-4′′), 63.15 (C-6′′). Signal assignments were performed by comparison to similar data from the literature [42,51,59].

Kaempferol 3-*O*-(2′′-*O*-galloyl)-β-d-glucoside (**13**, **16** or **19**) is a pale-yellow amorphous powder (yield of 3,9 mg). UV λ_max_: 260,300 sh, 348 nm. [M + H]^+^ *m*/*z* 601.1183. ^1^H NMR (800 MHz, DMSO-d6): δH 8.04 (2H, d, *J* = 8.8 Hz, H-2′, H-6′), 6.90 (2H, s, H-2′′′, H-6′′′), 6.89/6.93 (2H, d, *J* = 8.8 Hz, H-3′, H-5′), 6.45 (1H, d, *J* = 2.0 Hz, H-8), 6.21 (1H, d, *J* = 2.0 Hz, H-6), 5.47 (1H, d, *J* = 7.6 Hz, H-1′′), 3.62/3.38 (2H, H6′’), 3.51 -3.48 (4H, m, H- 2′′- H-5′′). ^13^C NMR (200 MHz, DMSO-d6): δC 163.30 (C-7), 162.11 (C-7′′′), 160.0 (C-5), 158.40 (C-4′), 155.50 (C-9), 155.46 (C-2), 145.96 (C-3′′′, C-5′′′), 138.86 (C-4′′′), 133.22 (C-3), 129.54 (C-2′,C-6′), 124.0 (C-1′′′), 120.90 (C-1′), 114.66 (C-3′, C-5′), 108.45 (C-2′′′, C-6′′′), 102.70 (C-10), 100.38 (C-1′′), 98.02 (C-6), 93.05 (C-8), 76.74 (C-5′′), 75.87 (C-3′′), 73.68 (C-2′′), 69.48 (C-4′′), 60.68 (C-6′′). Signal assignments were performed by comparison to similar data from the literature [43,52,60].

The mixture of kaempferol 3-*O*-β-d-glucopyranoside, kaempferol-(2′′-*O*-methyl)-4′-*O*-α-d-glucopyranoside **(17)** appears a pale yellow to dark amorphous powder (yield of 4.2 mg). UV λ_max_ = 266, 348 nm. [M + H]^+^ *m*/*z* 449.1079 and *m*/*z* 463.0865. ^1^H NMR (800 MHz, DMSO-d6) and ^13^C NMR (200 MHz, DMSO-d6) are shown in in Table 5. Correlations of HMBC spectrum are shown in Figure 2. Signal assignments were performed by comparison to similar data from the literature [42,51,54,59].

Kaempferol 3-*O*-glucuronide-6′′-*O*-methylester (**23**) is a pale-yellow liquid (yield of 0.5 mg). UV λ_max_ = 268, 320 nm. [M + H]^+^ *m*/*z* 477.1031. ^1^H NMR (800 MHz, DMSO-d6) δH 8.02 (2H, d, *J* = 8.9 Hz, H-2′ and 6′), 6.89 (2H, d, *J* = 8.9 Hz, H-3′ and 5′), 6.45 (1H, d, *J* = 2.0 Hz, H-8), 6.23 (1H, d, *J* = 2.0 Hz, H--6), 5.47 (1H, d, *J* = 7.7 Hz, H-1′′), 3.57 (3H, s, OMe). The HMBC spectrum showed three relevant correlations: the anomeric hydrogen (δ 5.47) with C3 (δ 133.58) from the flavonoid C-ring, the H5′′ (δ 3.73) from the sugar moiety and the methoxyl group (δ 3.57) both with C6′′ (δ 169.52) from the carboxyl group in the glucuronic acid. Analyses of HSQC and HMBC spectra confirmed the assignment of the carbon signals aggressed with previously reported data [42,51,60].

Quercetin 3-*O*-(6′′-*O*-*p*-coumaroyl)-β-d-glucopyranoside (**25**) is a light pink to dark amorphous powder (yield of 0,5 mg). UV λ_max_ = 271, 312 nm. [M + H]^+^ *m*/*z* 611.1393. ^1^H NMR (800 MHz, DMSO-d6) δH 7.66 (1H, dd, *J* = 8.5 Hz and *J* = 2.3 Hz, H6′), 7.52 (1H, d, *J* = 2.3 Hz, H-2′), 7.37 (2H, d, *J* = 8.4 Hz, H-2′′′, H-6′′′), 7.36 (1H, d, *J* = 15.7 Hz, H7′′′).6.83 (2H, d, *J* = 8.4 Hz, H-3′′, H-5′′′), 6.78 (1H, d, *J* = 8.4 Hz, H5′), 6.38 (1H, d, *J* = 2.1 Hz, H-8), 6.14 (1H, d, *J* = 2.1 Hz, H-6), 6.13 (1H, d, *J* = 15.7 Hz, H8′′′), 5.42 (1H, d, *J* = 7.8 Hz) and 4.12 (1H, dd, *J* = 11.5 Hz and *J* = 4.6 Hz, H-1′′′, H-6′′′). HSQC spectrum allowed assignment of carbon signals in agreement with data from the literature [42,51,61].

Kaempferol 3-*O*-(6′′-*O*-*p*-coumaroyl)-β-d-glucopyranoside (**27**) appears as light pink to dark amorphous powder (yield of 0,7 mg). UV λ_max_ = 268, 314 nm. [M + H]^+^ *m*/*z* 595.1418. ^1^H NMR (800 MHz, DMSO-d6) δH 8.05 (2H, d, *J* = 8.4 Hz, H-2′, H-6′), 7.34 (1H, d, *J* = 15.9 Hz, H-7′′′), 6.86 (2H, d, *J* = 8.7 Hz, H-2′′′, H-6′′′), 6.78 (2H, d, *J* = 8.4 Hz, H-3′, H-5′), 6.40 (1H, d, *J* = 2.1 Hz, H-8), 6.14 (1H, d, *J* = 2.1 Hz, H-6), 6.11 (1H, d, *J* = 15.9 Hz, H-8′′′), 5.41 (1H, d, *J* = 7.7 Hz, H-1′′), 4.10 (2H, d, *J* = 6.2 Hz, H-6′’). The HMBC spectrum showed one relevant correlation confirming the *p*-coumaroyl position: the H6′′ (δ 4.10) from the sugar moiety with the C9′′′ from the *p*-coumaroyl group. HSQC and HMBC spectra reinforced the assignment of the carbon signals in accordance to the literature [42,51,62].

Kaempferol (**29**) is a pale-yellow amorphous powder (yield of 2,1 mg). UV λmax (MeOH): 260,300 sh, 348 nm. [M + H]^+^ *m*/*z* 287.0546. ^1^H NMR (800 MHz, DMSO-d6): δH 12.48 (1H, s, OH-C5), 10.83 (1H, s, OH-C7), 10.13 (1H, s, OH-C4′), 8.05 (2H, d, *J* = 8.9 Hz, H-2′, H-6′), 6.94 (2H, d, *J* = 8.9 Hz, H-3′, H-5′), 6.46 (1H, d, *J* = 2.0 Hz, H-8), 6.21 (1H, d, *J* = 2.0 Hz, H-6). ^13^C NMR (200 MHz, DMSO-d6): δC 176.36 (C-4), 164.38 (C-7), 161.15 (C-5), 159.67 (C-4′), 156.63 (C-9), 147.27 (C-2), 136.11 (C-3), 129.95 (C-2′, C-6′), 122.12 (C-1′), 115.91 (C- 3′, C-5′), 103.49 (C-10), 96.68 (C-6), 93.95 (C-8) [42,51,63,64].

## Figures and Tables

**Table 1:** Natural products reported for *Tibouchina* species.

Species	Metabolite Subclass	Compound	Plant Material	Reference
*T. candolleana* Cogn.	flavone/isoflavoid/steroid/triterpene	luteolin/genistein/β- sitosterol/α- and β-amyrin, oleanolic and ursolic acids	aerial parts	[21]
*T. ciliaris* (Vent.) Cogn.	flavonol	kaempferol 7-*O*-*p*-coumaroyl, quercetin 3-*O*-rhamnopyranoside, quercetin 3-*O*-arabnoside	leaves	[30]
*T. grandiflora* Cogn.	anthocyanin/flavonol	peonidin 3-sophoroside, peonidin 3-sambubioside, malvidin 3,5-diglucoside, malvidin 3-(*p*-coumaroyl)-sambubioside-5-glucoside/quercetin 3-*O*-β-d-glucuronide, quercetin 3-*O*-β-d-glucopyranoside, quercetin 3-*O*-β-d-galactopyranoside, quercetin 3-*O*-α-l-rhamnopyranoside, quercetin 3-*O*-β-l-arabinopyranoside, quercetin 3-*O*-β-d-(6″-*p*-coumaroyl)-glucopyranoside	leaves/flowers	[23,31]
*T. granulosa* (Desr.) Cogn.	anthocyanin/flavone/flavonol/proanthocyanidin	petunidin, pelargonidin/hispidulin 7-*O*-glucoside/isorhamnetin 3-*O*-glucuronide, isorhamnetin 3-*O*-diglucoside, isorhamnetin 3-*O*-rutinoside, quercetin 3-(*O*-galloyl)-hexoside/β-type procyanidin monomer, dimer, trimer and pentamer	flowers/leaves	[32,33]
*T. lepidota* (Bonpl.) Baill.	anthocyanin/flavonol/phenolic acid	malvidin 3-(*p*-coumaryl-glucoside)-5-(acetyl-xyloside)/quercetin 3-*O*-arabnoside, quercetin, quercetin 3-glucoside, isorhamnetin 3-rutinoside/gallic acid, 2,3,5-trihydroxybenzoic acid	flowers	[29]
*T. multiflora* Cogn.	tannin	nobotanins O and P	leaves	[34]
*T. paratropica* (Grised.) Cogn.	flavonol/phenolic derivative	isoquercitrin/2,8-dihydroxy-7H-furo (2,3-f)-chromen-7-one	aerial parts	[20]
*T. pereirae* Brade & Markgr.	flavonol	isorhamnetin 3-*O*-galloyl-glucoside, isorhamnetin-3-*O*-glucoside, kaempferol 3-*O*-rutinoside, quercetin 3-*O*-(galloyl)-glucoside, rutin	aerial parts	[35]
*T. pulchra* (Cham.) Cogn.	flavone/flavonol/phenolic acid	luteolin/kaempferol 3-*O*-galactoside, kaempferol 3-*O*-glucoside, myricetin 3-*O*-galactoside, myricetin 3-*O*-glucoside, quercetin, myricetin, kaempferol/gallic acid	leaves	[10,36]
*T. semidecantra* (Mart & Schrank ex DC.) Cogn.	anthocyanin/flavonol/proanthocyanidin/tannin	malvidin 3-(*p*-coumaroylglucoside)-5-glucoside/quercetin, myricetin, quercetin 3-*O*-(6″-*O*-galloyl) galactoside, quercetin 3-*O*-α-l-(2″-*O*-acetyl) arabinofuranoside, quercetin 3-*O*-arabnoside, quercetin 3-*O*-rhamnopyranoside/leucodelphinidin, leucocyanidin/1,2,6-tri-*O*-galloyl-β-d-glucose, 1,4,6-tri-O-galloyl-β-d-glucoside, 1,2,3,6-tetra-*O*-galloyl-β-d-glucoside, nobotanin A, B, C, D, E and F, casuarictin, pedunculagin, praecoxin A and B, casuarinin, 2,3-*O*-(S)-hexahydroxydiphenoyl-d-glucopyranoside, castalagin, vescalagin, 1-*O*-methylvescalaginnobotanins A, B, F, 3,3′-*O*-dimethyl ellagic acid 4-*O*-α-l-rhamnopyranoside	aerial parts	[37,38,39,40]
*T. urvilleana* (DC.) Cong.	anthocyanin/flavone/flavonol/steroid/triterpene	malvidin 3-*O*-(6-*O*-*p*-coumaryl-β-d-glucopyranoside)- 5-*O*-(2-*O*-acetyl-β-d-xylopyranosyl)/hispidulin 7-*O*-β-d-glucopyranoside/quercetin 3-*O*-arabinoside/β- sitosterol/α- and β-amyrin, glutinol, taraxerol, oleanolic and ursolic acids	aerial parts	[27,28]

**Table 2:** Chromatographic and spectrometric data (UPLC-PAD-ESI-QTOF-MS) of phenolic constituents from *T. pulchra* petal extracts (acidified methanol).

Compound	RT ^1^ (min)	UV/VIS (nm)	Mass Spectrum MS/MS	Suggestion
1	00.97	278	L.Q. ^3^	Phenolic acid
2	01.13	278, (sh ^2^) 308	L.Q.	Cinnamic acid derivative
3	01.55	278	L.Q.	Phenolic acid
4	01.76	278	L.Q.	Phenolic acid
5	02.95	278, (sh) 308	L.Q.	Cinnamic acid derivative
6	03.65	278	L.Q.	Phenolic acid
7	12.69	270	453.0083 [M + H]^+^, 303.0134 [M − 150]^+^	N.I. ^4^
8	14.05	268, 294 (sh), 354	481.0967 [M + H]^+^, 319.0446 [M − 162]^+^	Myricetin galactoside
9	14.70	268, 294 (sh), 354	481.0964 [M + H]^+^, 319.0445 [M − 162]^+^	Myricetin glucoside
**10**	16.33	269, 290 (sh), 354	639.0946 [M+Na]^+^, 617.1121 [M + H]^+^, 303.0498 [M − 314]^+^	**Quercetin galloylhexoside**
11	18.25	269, 290 (sh), 355	465.1020 [M + H]^+^, 303.0499 [M − 162]^+^	Quercetin hexoside
**12**	18.89	269, 290 (sh), 355	479.0804 [M + H]+, 303.0493 [M − 176]^+^	**Quercetin glucuronide**
**13**	19.36	266,290,350	601.1183 [M + H]^+^, 287.0552 [M − 314]^+^	**Kaempferol galloylhexoside**
14	19.95	270	453.0083 [M + H]^+^, 303.0134 [M − 150]^+^	N.I.
**15**	21.42	266, 346	471.0894 [M + Na]^+^, 449.1073 [M + H]^+^, 287.0549 [M − 162]^+^	**Kaempferol hexoside**
**16**	22.00	266,290,350	601.1184 [M + H]^+^, 287.0551 [M − 314]^+^	**Kaempferol galloylhexoside**
**17**	23.18	266, 348	449.1079 [M + H]^+^, 287.0551 [M − 162]^+^/463.0865 [M + H]^+^, 287.0551 [M − 176]^+^	Mixture: Kaempferol 3-*O*-β-d-glucopyranoside (Astragalin)/**Kaempferol-(2″-*O*-methyl)-4′-*O*-α-d-glucopyranoside**
**18**	23.93	266, 355	441.0790 [M + Na]^+^, 419.0971 [M + H]^+^, 287.0551 [M − 132]^+^	**Kaempferol pentoside**
**19**	24.81	266,290,350	623.1000 [M + Na]^+^, 601.117 [M + H]^+^, 287.0547 [M − 314]^+^	**Kaempferol galloylhexoside**
**20**	25.98	266, 355	441.0785 [M + Na]^+^, 419.0959 [M + H]^+^, 287.0545 [M − 132]^+^	**Kaempferol pentoside**
**21**	27.87	268, 314	595.1445 [M + H]^+^, 287.0551 [M − 308]^+^	**Kaempferol *p*-coumaroylhexoside**
**22**	28.24	282, 305(sh), 530	799.2077 [M + H]^+^, 625.1552 {M − 174]^+^, 491.1176 [M − 308]^+^, 317.0655 [M − 482]^+^	**Petunidin *p*-coumaroylhexoside acetylpentoside**
**23**	29.04	268, 320, 530	499.0839 [M + Na]^+^, 477.1031 [M + H]^+^, 287.0547 [M − 190]^+^/771.2138 [M + H]^+^, 317.0665 [M − 454]^+^	Mixture- **Kaempferol 3-*O*-glucuronide-6″-*O*-methylester/Petunidin derivative**
**24**	30.82	282, 310(sh), 534	813.2243 [M + H]^+^, 639.1716 [M − 174]^+^, 505.1336 [M − 308]^+^, 331.0812 [M − 482]^+^	Malvidin *p*-coumaroylhexoside acetylpentoside
**25**	32.68	271, 312	633.1203 [M + Na]^+^, 611.1393 [M + H]^+^, 303.0496 [M − 308]^+^	**Quercetin 3-*O*-(6″-*O*-*p*-coumaroyl)-β-d-glucopyranoside (Helichrysoside)**
26	34.78	266, 349	593.0892 [M + H]^+^, 285.0603 [M − 308]^+^	N.I.
**27**	35.27	268, 314	617.1258 [M + Na]^+^, 595.1437 [M + H]^+^, 287.0546 [M − 308]^+^	**Kaempferol 3-*O*-(6″-*O*-*p*-coumaroyl)-β-d-glucopyranoside (Tiliroside**)
**28**	36.17	268, 314	617.1256 [M + Na]^+^, 595.1418 [M + H]^+^, 287.0545 [M − 308]^+^	**Kaempferol *p*-coumaroylhexoside**
29	37.48	270, 368	287.0546 [M + H]^+^	Kaempferol
**30**	37.98	268, 314	617.1248 [M + Na]^+^, 595.1455 [M + H]^+^, 287.0549 [M − 308]^+^	**Kaempferol *p*-coumaroylhexoside**

^1^ retention time in minutes, ^2^ shoulder, ^3^ low quality spectrum, ^4^ not identified. Numbers highlighted in bold indicate compounds identified for the first time in *T. pulchra*.

**Table 3:**
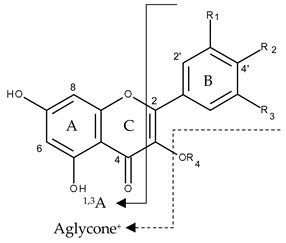
Flavonol structures and substituents groups in *T. pulchra* flower extracts identified by UPLC-PAD-ESI-QTOF-MS and NMR (only bold compounds).

Compound	R1	R2	R3	R4
8	OH	OH	OH	galactosyl
9	OH	OH	OH	glucosyl
10	OH	OH	H	galloylhexoside
11	OH	OH	H	hexosyl
12	OH	OH	H	glucuronyl
**13**	H	OH	H	galloylhexoside
15	H	OH	H	hexosyl
**16**	H	OH	H	galloylhexoside
**17**	H	OH	H	3-*O*-β-d-glucopyranosyl
**17**	H	2″-*O*-methyl-4′-*O*-α-d-glucopyranoside	H	H
18	H	OH	H	pentosyl
**19**	H	OH	H	galloylhexoside
20	H	OH	H	pentosyl
21	H	OH	H	*p*-coumaroylhexoside
**23**	H	OH	H	3-*O*-glucuronide-6″-*O*-methylester
**25**	OH	OH	H	3-*O*-(6″-*O*-*p*-coumaroyl)-β-d-glucopyranosyl
**27**	H	OH	H	3-*O*-(6″-*O*-*p*-coumaroyl)-β-d-glucopyranosyl
28	H	OH	H	*p*-coumaroylhexoside
**29**	H	OH	H	H
30	H	OH	H	*p*-coumaroylhexoside

* R1, R2, R3 and R4 indicate substituents. In the chemical formula continuous arrow indicates retro Dies–Alder fragmentation, and dotted arrow indicates the usual acyl and glucosyl lost.

**Table 4:**
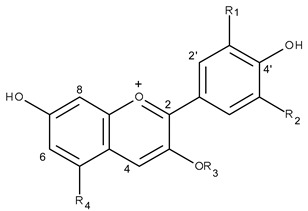
Anthocyanin structures and substituents groups in *T. pulchra* flower extracts identified by UPLC-PAD-ESI-QTOF-MS.

Compound	R1	R2	R3	R4
22	OH	OCH_3_	*p*-coumaroylhexoside	acetylpentoside
24	OCH_3_	OCH_3_	*p*-coumaroylhexoside	acetylpentoside

* R1, R2, R3 and R4 indicate substituents.

**Table 5:**
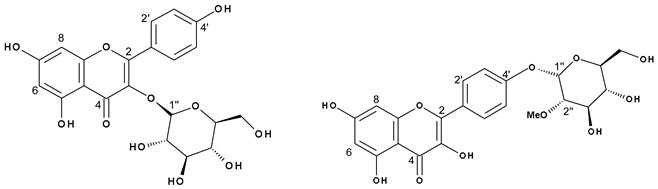
Chemical formula and NMR data of mixture 17: Astragalin and Kaempferol-(2″-*O*-methyl)-4′-*O*-α-D-glucopyranoside.

	Astragalin			Kaempferol-(2″-*O*-methyl)-4′-*O*-α-d-glucopyranoside		
Carbon Number	^1^H	^13^C	HMBC	^1^H	^13^C	HMBC
2	-	156.62	-	-	147.27	-
3	-	133.63	-	-	136.10	-
4	-	177.92	-	-	176.35	-
5	-	161.65	-	-	161.13	-
6	6.22 d (*J* = 2.0 Hz)	99.16	C5, C7, C8, C10	6.21 d (*J* = 2.0 Hz)	98.68	C5, C7, C8, C10
7	-	164.62	-	-	164.40	-
8	6.46 sl	94.12	C4, C6, C7, C9, C10	6.46 sl	93.95	C6, C7, C9
9	-	156.82	-	-	156.71	-
10	-	104.45	-	-	103.48	-
1′	-	122.33	-	-	122.10	-
2′,6′	8.04 d (*J* = 8.4 Hz)	131.33	C2, C4′, C3′ or 5′, C2′ or 6′	8.04 d (*J* = 8.5 Hz)	129.95	C2, C3′ or 5′, C4′
3′,5′	6.89 d (*J* = 8.4 Hz)	115.58	C1, C3′ or 5′, C4′	6.93 d (*J* = 8.5 Hz)	115.91	C1′, C3′ or 5′, C4′
4′	-	160.44	-	-	159.68	-
1″	5.46 d (*J* = 7.6 Hz)	101.81	C3, C5″	4.51 d (*J* = 3.6 Hz)	100.12	C2″, OMe
2″	3.18 m	74.67	C1″, C3″, C4″	3.37 m	73.84	C3″
3″	3.22 m	76.88	C2″, C4″	3.18 m	72.44	C2″
4″	3.09 m	70.33	C6″, C5″	3.29 m	73.04	C1″
5″	3.09 m	77.96	C6″, C4″	3.04 m	70.79	C6″, C4″
6″	3.58 d (*J* = 11.6 Hz) 3.33 d (*J* = 11.6 Hz)	61.28	C5″, C4″	3.62 d (*J* = 11.7 Hz) 3.44 m	61.42	C5″, C4″
2″*O*Me	-	-	-	3.26 s	54.74	C1″

## Supplementary Materials

The following are available online, Figure S1: MS^+^ spectra of compounds **1** to **30**, Figures S2–S8: NMR spectra of kaempferol 3-*O*-(2″-galloyl)-β-d-glucopyranoside (**13**, **16** or **19**), Figures S9–S15: NMR spectra of kaempferol 3-*O*-(6″-galloyl)-β-d-glucopyranoside (**13**, **16** or **19**), Figures S16–S21: NMR spectra of mixture (**17**), Figures S22–S26: NMR spectra of kaempferol 3-*O*-glucoronide-6″-*O*-methylester (**23**), Figures S27–S31: NMR spectra of quercetin 3-*O*-(6″-*p*-coumaroyl)-β-d-glucopyranoside (**25**), Figures S32–S38: NMR spectra of kaempferol 3-*O*-(6″-*p*-coumaroyl)-β-d-glucopyranoside (**27**), Figures S39–S41: NMR spectra of kaempferol (**29**).
