# Supplementary material for: Acylated Flavonoid Glycosides are the Main Pigments that Determine the Flower Colour of the Brazilian Native Tree Tibouchina pulchra (Cham.) Cogn
Source: Molecules. 2019 Feb 16;24(4):718. doi: 10.3390/molecules24040718 (PMC6412660; doi:10.3390/molecules24040718)
Supplement: Supplementary file 1 [file molecules-24-00718-s001.zip › Article_Supplementary figures feb 15.pdf]

molecules

MDPI

Article

Acylated flavonoid glycosides are the main pigments that determine the flower colour of the Brazilian native tree *Tibouchina pulchra* (Cham.) Cogn.

Fernanda Mendes Rezende [1,*], Marcelo José Pena Ferreira [1], Mads Hartvig Clausen [2], Magdalena Rossi [1] and Claudia Maria Furlan [1,*]

[1] Botany Department, Institute of Bioscience, University of São Paulo (USP), São Paulo, Brazil; marcelopena@ib.usp.br (M.J.P.F.); magdarossirosso@gmail.com (M.R.)

[2] Center for Nanomedicine and Theranostics, Department of Chemistry, Technical University of Denmark, Kgs. Lyngby, Denmark; mhc@kemi.dtu.dk

* Correspondence: rezendefm@usp.br (F.M.R.); furlancm@ib.usp.br (C.M.F.); Tel.: +55-011-3091-8065

SUPPLEMENTARY MATERIAL

Figure S1. MS+ spectra of compounds **1** to **30**. Structures represent the proposed compound and its main fragmentation……1

Figure S2. ^{1}H NMR spectrum (DMSO-*d6*) of kaempferol 3-*O*-(2′′-galloyl)-β-D-glucopyranoside (**13**, **16** or **19**)……8

Figure S3. Zoom from 5.2 to 8.5 ppm, ^{1}H NMR spectrum (DMSO-*d6*) of kaempferol 3-*O*-(2′′-galloyl)-β-D-glucopyranoside (**13**, **16** or **19**) ……8

Figure S4. HSQC NMR spectrum (DMSO-*d6*) of kaempferol 3-*O*-(2′′-galloyl)-β-D-glucopyranoside (**13**, **16** or **19**)……9

Figure S5. Zoom from 2.7 to 3.8 ppm, HSQC NMR spectrum (DMSO-*d6*) of kaempferol 3-*O*-(2′′-galloyl)-β-D-glucopyranoside (**13**, **16** or **19**)……9

Figure S6. HMBC NMR spectrum (DMSO-*d6*) of kaempferol 3-*O*-(2′′-galloyl)-β-D-glucopyranoside (**13**, **16** or **19**)……10

Figure S7. Zoom from 2.7 to 3.9 ppm, HMBC NMR spectrum (DMSO-*d6*) of kaempferol 3-*O*-(2′′-galloyl)-β-D-glucopyranoside (**13**, **16** or **19**) ……10

Figure S8. Zoom from 5.2 to 8.6 ppm, HMBC NMR spectrum (DMSO-*d6*) of kaempferol 3-*O*-(2′′-galloyl)-β-D-glucopyranoside (**13**, **16** or **19**) ……11

Figure S9. ^{1}H NMR spectrum (DMSO-*d6*) of kaempferol 3-*O*-(6′′-galloyl)-β-D-glucopyranoside (**13**, **16** or **19**)……11

Figure S10. Zoom from 5.1 to 8.1 ppm, ^{1}H NMR spectrum (DMSO-*d6*) of kaempferol 3-*O*-(6′′-galloyl)-β-D-glucopyranoside (**13**, **16** or **19**). ……12

Figure S11. HSQC NMR spectrum (DMSO-*d6*) of kaempferol 3-*O*-(6′′-galloyl)-β-D-glucopyranoside (**13**, **16** or **19**)……12

Figure S12. Zoom from 3.0 to 4.6 ppm, HSQC NMR spectrum (DMSO-*d6*) of kaempferol 3-*O*-(6′′-galloyl)-β-D-glucopyranoside (**13**, **16** or **19**)…… 13

Figure S13. HMBC NMR spectrum (DMSO-d6) of kaempferol 3-O-(6′′-galloyl)-β-D-glucopyranoside (13, 16 or 19)……13

Figure S14. Zoom from 2.8 to 4.9 ppm, HMBC NMR spectrum (DMSO-*d6*) of kaempferol 3-*O*-(6′′-galloyl)-β-D-glucopyranoside (**13**, **16** or **19**)…… .14

Figure S15. Zoom from 5.2 to 8.4 ppm, HMBC NMR spectrum (DMSO-*d6*) of kaempferol 3-*O*-(6′′-galloyl)-β-D-glucopyranoside (**13**, **16** or **19**)…… 14

Figure S16. ^{1}H NMR spectrum (DMSO-*d6*) of mixture (**17**)……15

Figure S17. Zoom from 5.2 to 8.2 ppm, ^{1}H NMR spectrum (DMSO-*d6*) of mixture (**17**)……15

Figure S18. ^{13}C NMR spectrum (DMSO-*d6*) of mixture (**17**)……16

Figure S19. HMBC NMR spectrum (DMSO-*d6*) of mixture (**17**)……16

Figure S20. Zoom from 2.4 to 4.5 ppm, HMBC NMR spectrum (DMSO-*d6*) of mixture (**17**)……17

Figure S21. Zoom from 4.8 to 8.6 ppm, HMBC NMR spectrum (DMSO-*d6*) of mixture (**17**)……17

Figure S22. ^{1}H NMR spectrum (DMSO-*d6*) of kaempferol 3-*O*-glucoronide-6′′-*O*-methylester (**23**)……18

Figure S23. Zoom from 5.1 to 8.1 ppm, ^{1}H NMR spectrum (DMSO-*d6*) of kaempferol 3-*O*-glucoronide-6′′-*O*-methylester (**23**)……18

Figure S24. HSQC NMR spectrum (DMSO-*d6*) of kaempferol 3-*O*-glucoronide-6′′-*O*-methylester (**23**)……19

Figure S25. Zoom from 2.9 to 4.5 ppm, HSQC NMR spectrum (DMSO-*d6*) of kaempferol 3-*O*-glucoronide-6′′-*O*-methylester (**23**)……19

Figure S26. Zoom from 5.0 to 8.6 ppm, HMBC NMR spectrum (DMSO-*d6*) of kaempferol 3-*O*-glucoronide-6′′-*O*-methylester (**23**)……20

Figure S27. ^{1}H NMR spectrum (DMSO-*d6*) of quercetin 3-*O*-(6′′-*p*-coumaroyl)-β-D-glucopyranoside (**25**)……20

Figure S28. Zoom from 5.1 to 8.1 ppm, ^{1}H NMR spectrum (DMSO-*d6*) of quercetin 3-*O*-(6′′-*p*-coumaroyl)-β-D-glucopyranoside (**25**)……21

Figure S29. HSQC NMR spectrum (DMSO-*d6*) of quercetin 3-*O*-(6′′-*p*-coumaroyl)-β-D-glucopyranoside (**25**)……21

Figure S30. Zoom from 2.7 to 4.7 ppm, HSQC NMR spectrum (DMSO-*d6*) of quercetin 3-*O*-(6′′-*p*-coumaroyl)-β-D-glucopyranoside (**25**)……22

Figure S31. Zoom from 5.0 to 8.8 ppm, HSQC NMR spectrum (DMSO-*d6*) of quercetin 3-*O*-(6′′-*p*-coumaroyl)-β-D-glucopyranoside (**25**)……22

Figure S32. ^{1}H NMR spectrum (DMSO-*d6*) of kaempferol 3-*O*-(6′′-*p*-coumaroyl)-β-D-glucopyranoside (**27**)……23

Figure S33. Zoom from 5.1 to 8.1 ppm, ^{1}H NMR spectrum (DMSO-*d6*) of kaempferol 3-*O*-(6′′-*p*-coumaroyl)-β-D-glucopyranoside (**27**)……23

Figure S34. HSQC NMR spectrum (DMSO-*d6*) of kaempferol 3-*O*-(6′′-*p*-coumaroyl)-β-D-glucopyranoside (**27**)……24

Figure S35. Zoom from 3.0 to 4.6 ppm, HSQC NMR spectrum (DMSO-*d6*) of kaempferol 3-*O*-(6′′-*p*-coumaroyl)-β-D-glucopyranoside (**27**)……24

Figure S36. HMBC NMR spectrum (DMSO-*d*6) of kaempferol 3-*O*-(6′′-*p*-coumaroyl)-β-D-glucopyranoside (**27**)……25
Figure S37. Zoom from 2.9 to 4.8 ppm, HMBC NMR spectrum (DMSO-*d*6) of kaempferol 3-*O*-(6′′-*p*-coumaroyl)-β-D-glucopyranoside (**27**)……25
Figure S38. Zoom from 5.2 to 8.4 ppm, HMBC NMR spectrum (DMSO-*d*6) of kaempferol 3-*O*-(6′′-*p*-coumaroyl)-β-D-glucopyranoside (**27**)……26
Figure S39. ^{1}H NMR spectrum (DMSO-*d*6) of kaempferol (**29**)……26
Figure S40. Zoom from 6.1 to 8.2 ppm, ^{1}H NMR spectrum (DMSO-*d*6) of kaempferol (**29**)……27
Figure S41. ^{13}C NMR spectrum (DMSO-*d*6) of kaempferol (**29**)……27
Table S1. NMR data of ^{1}H, ^{13}C and HMBC for kaempferol 3-*O*-(6′′-*O*-galloyl)-β-D-glucopyranoside and Kaempferol 3-*O*-(2′′-*O*-galloyl)-β-D-glucopyranoside (13, 16 or 19)……28
Table S2. NMR data of ^{1}H, ^{13}C and HMBC for kaempferol 3-*O*-glucuronide-6''-*O*-methylester (**23**)……29
Table S3. NMR data of ^{1}H, ^{13}C and HMBC for quercetin 3-*O*-(6′′-*O*-*p*-coumaroyl)-β-D-glucopyranoside (**25**) and Kaempferol 3-*O*-(6′′-*O*-*p*-coumaroyl)-β-D-glucopyranoside (**27**)……30
Table S4. NMR data of ^{1}H and ^{13}C for kaempferol (29)……31

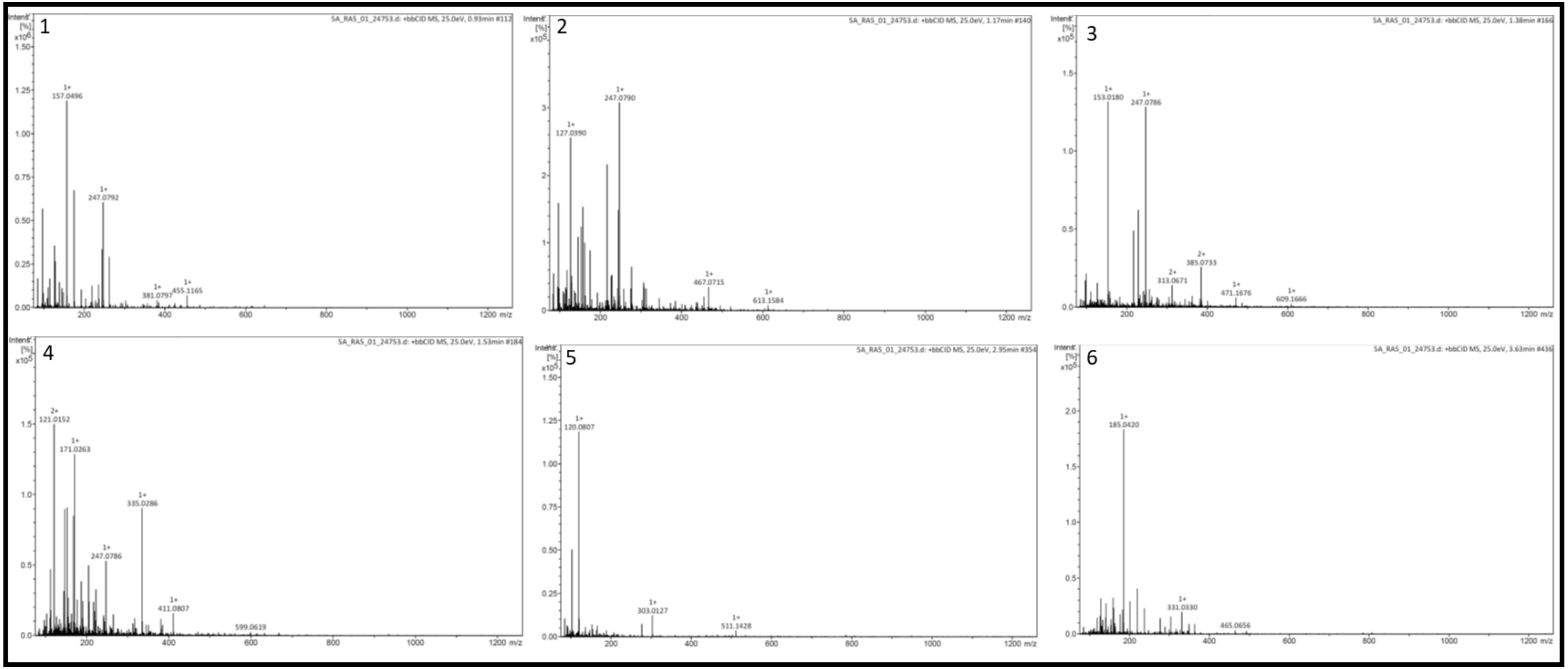

Figure S1. MS⁺ spectra of compounds **1** to **30**. Structures represent the proposed compound and its main fragmentation.

Figure S1 (continue).

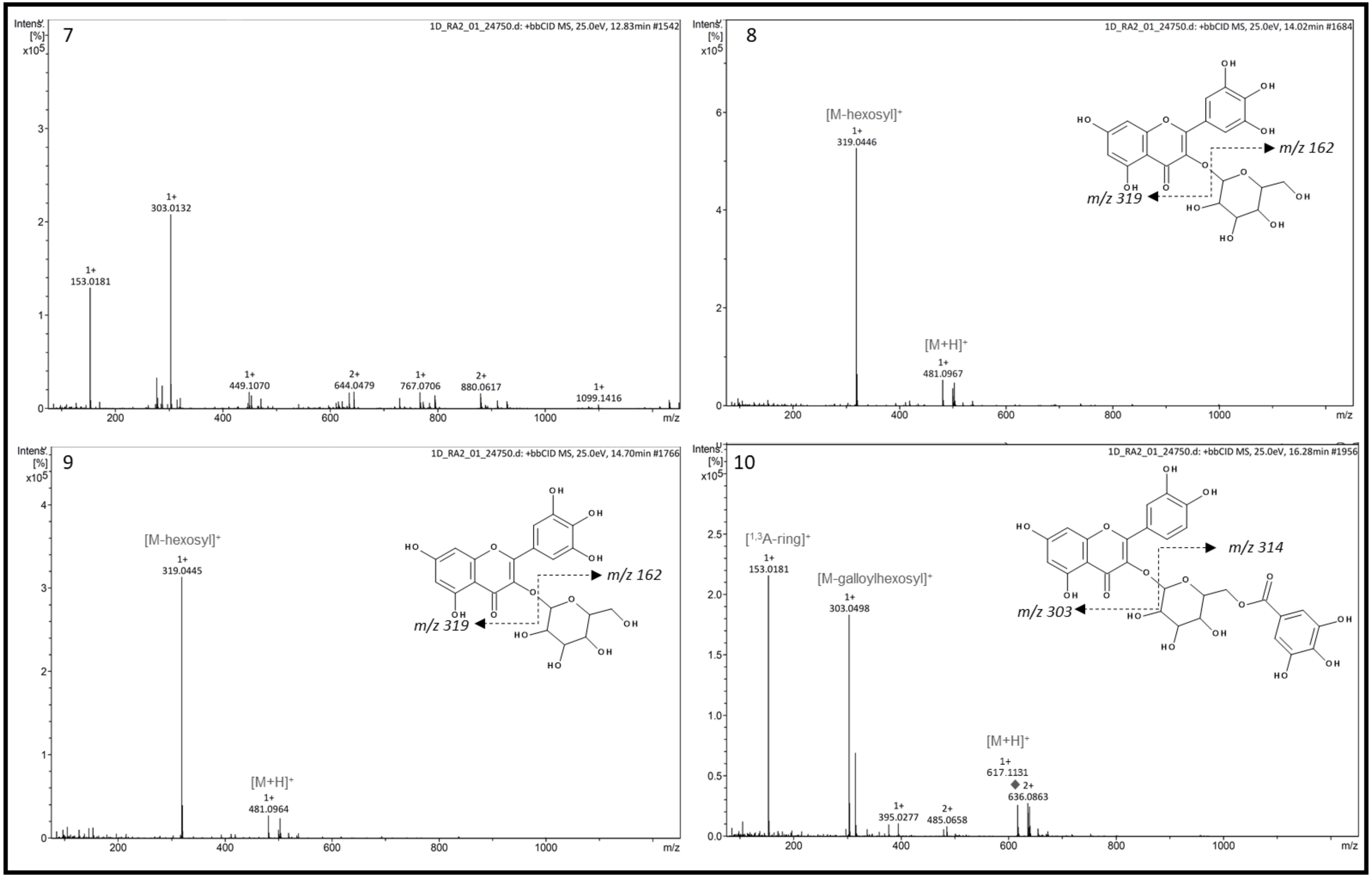

Figure S1 (continue).

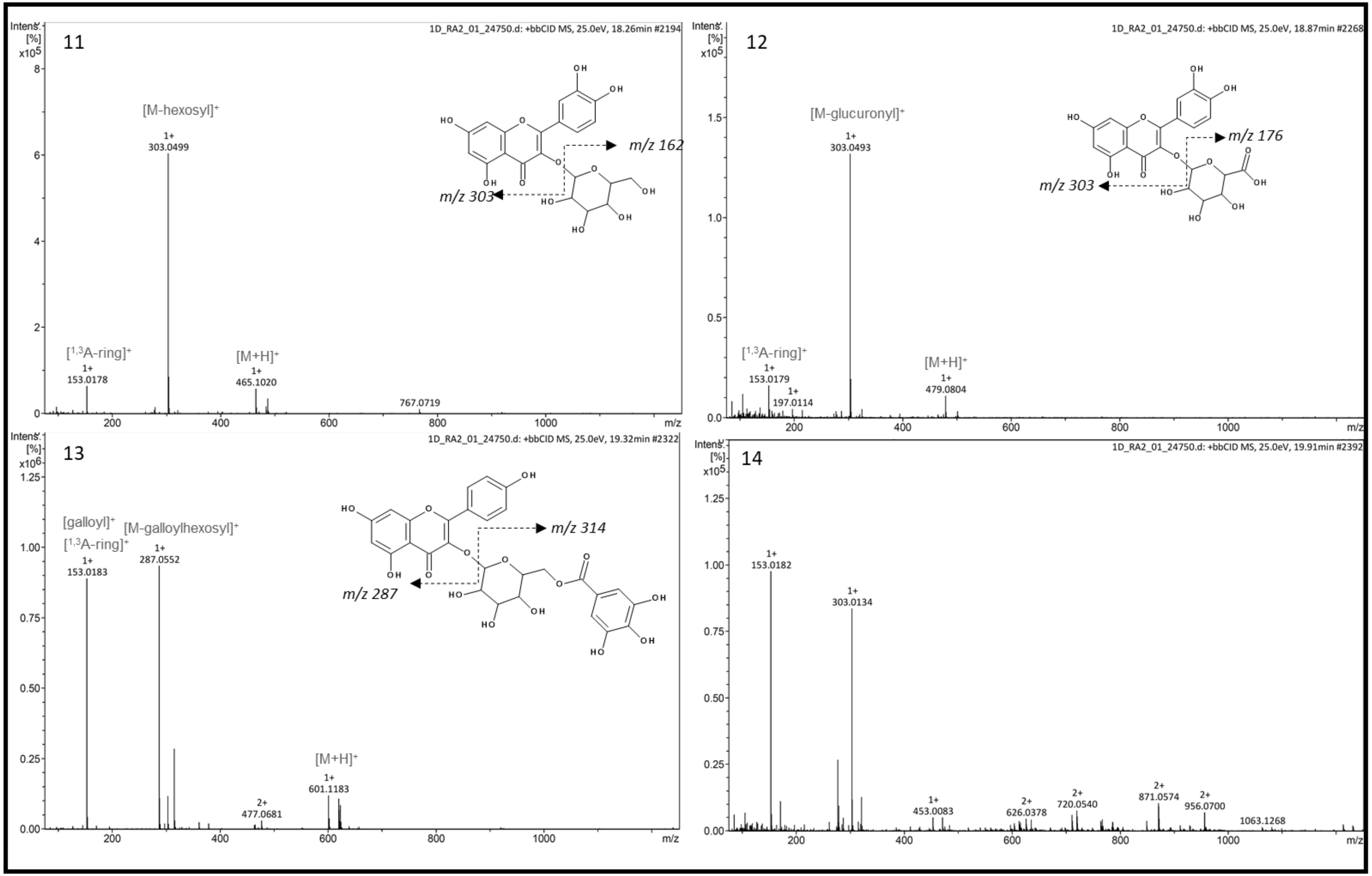

109 **Figure S1 (continue).**

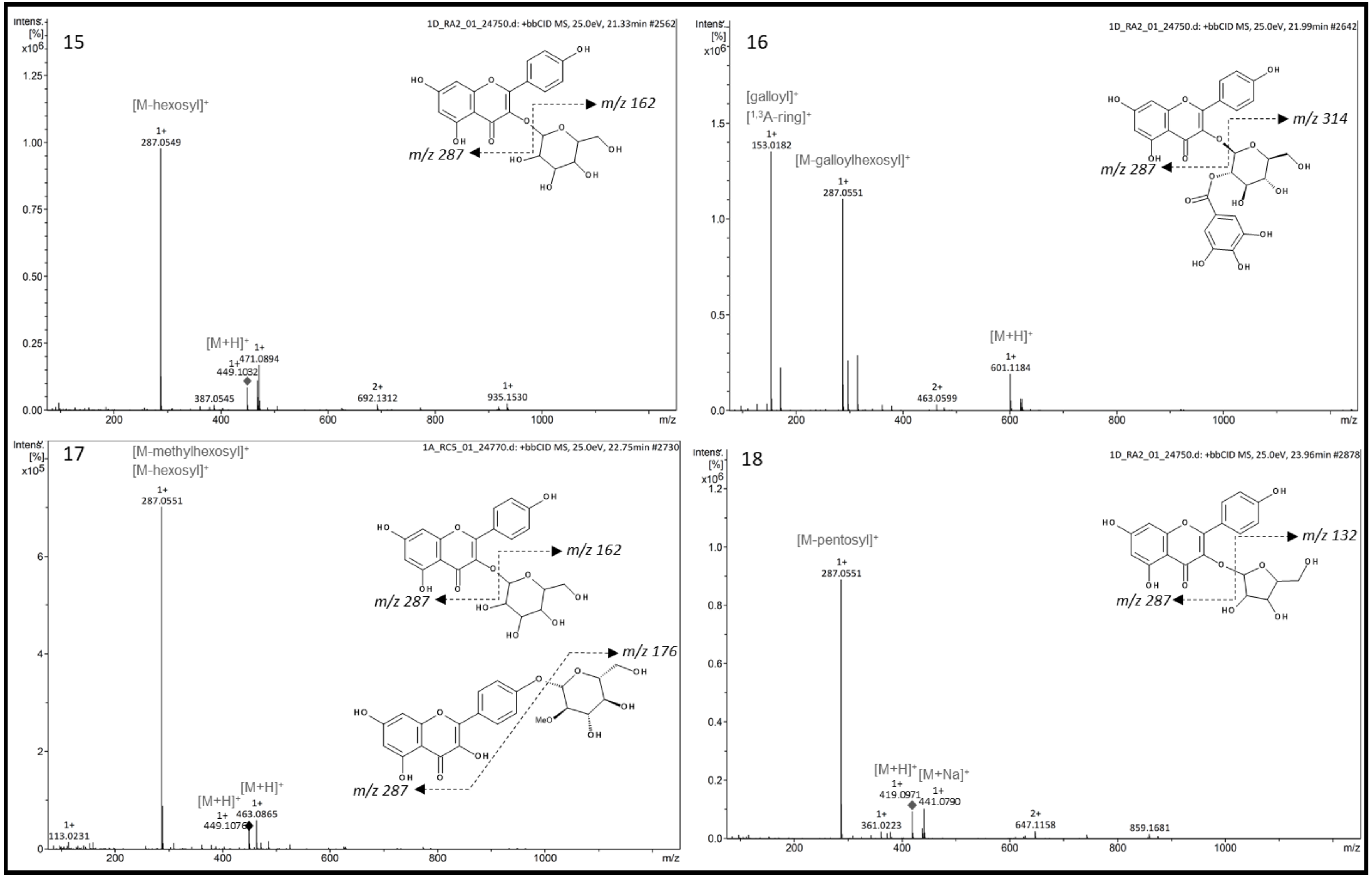

110

Figure S1 (continue).

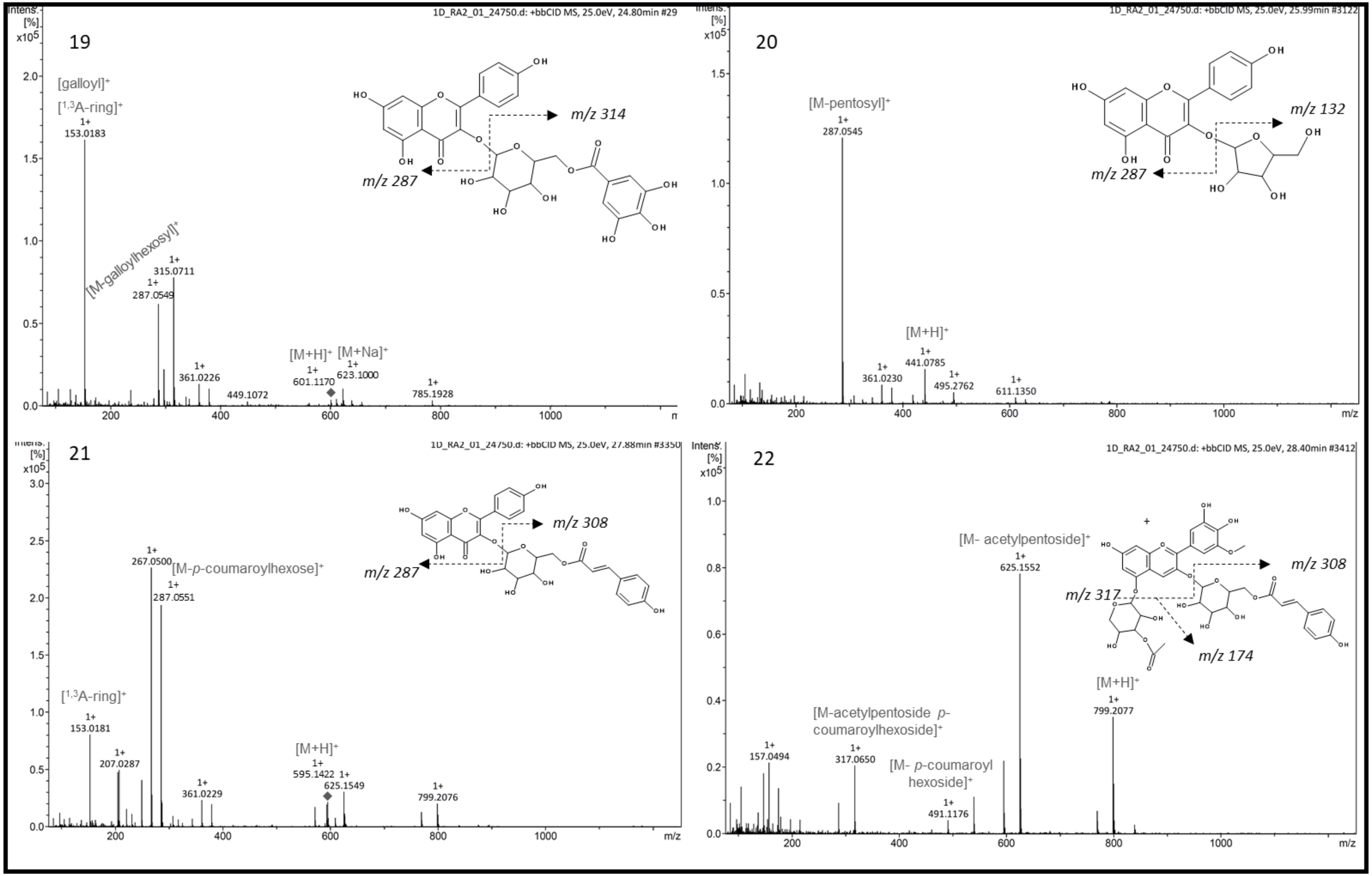

Figure S1 (continue).

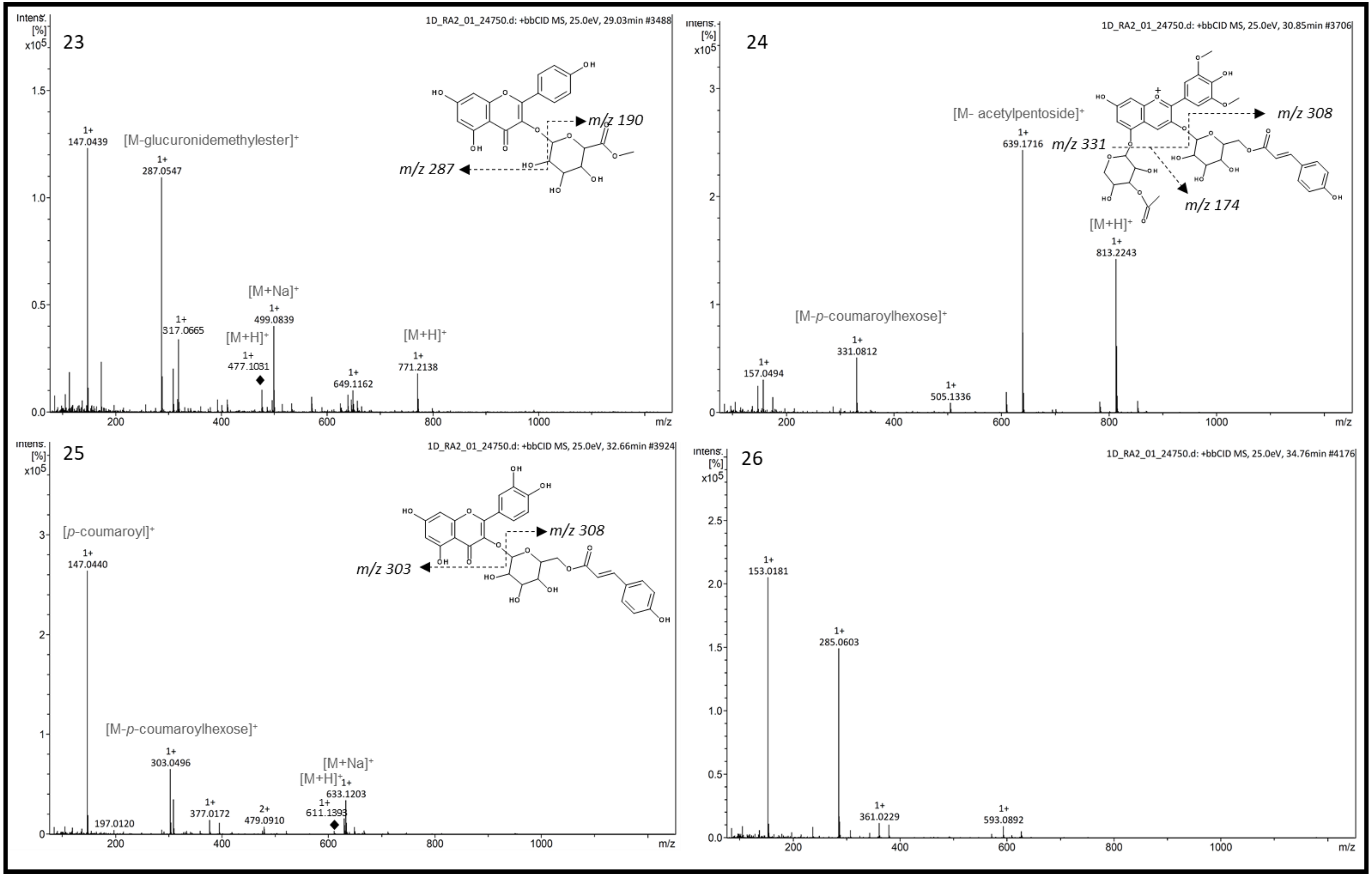

Figure S1 (continue).

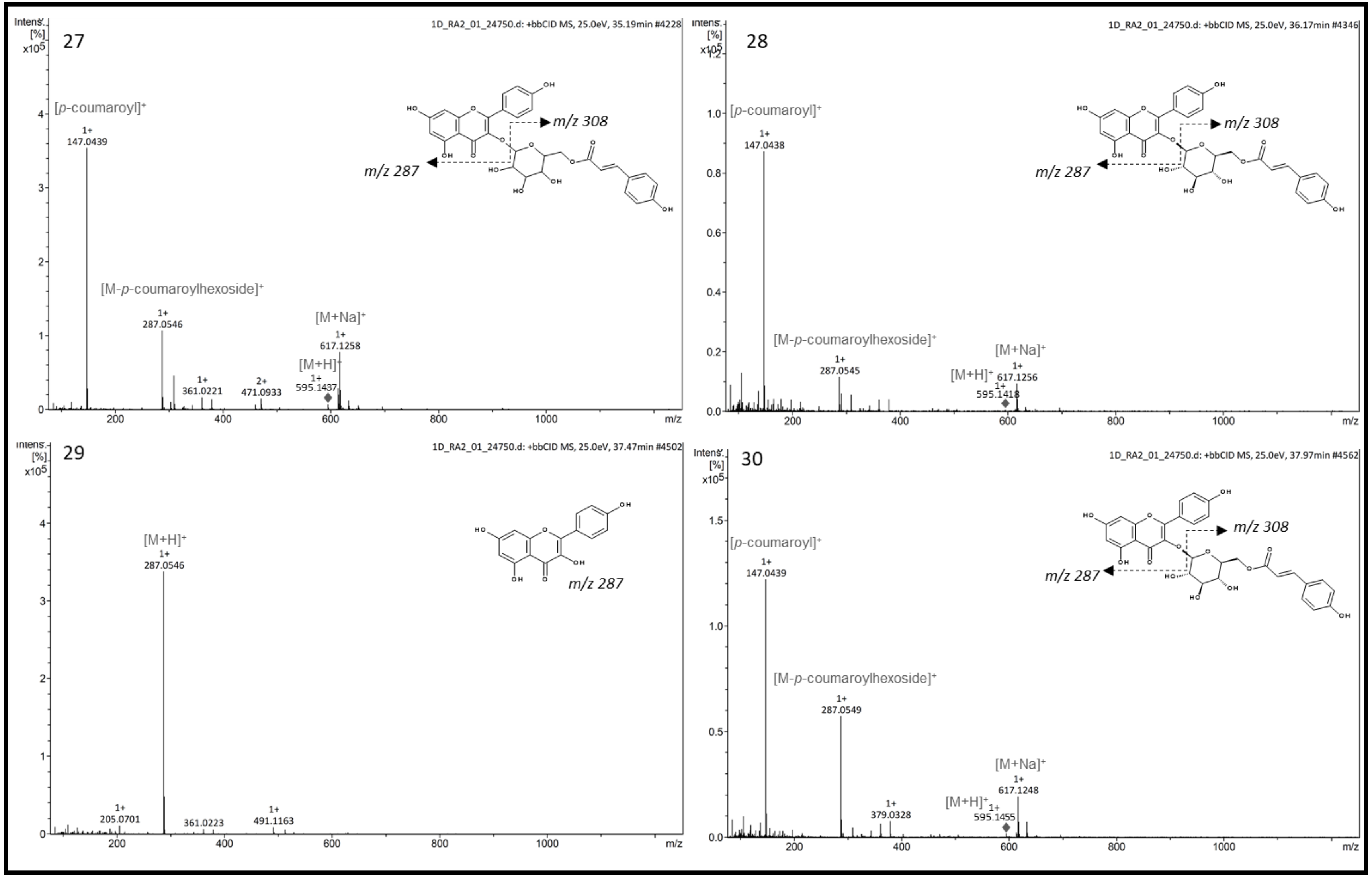

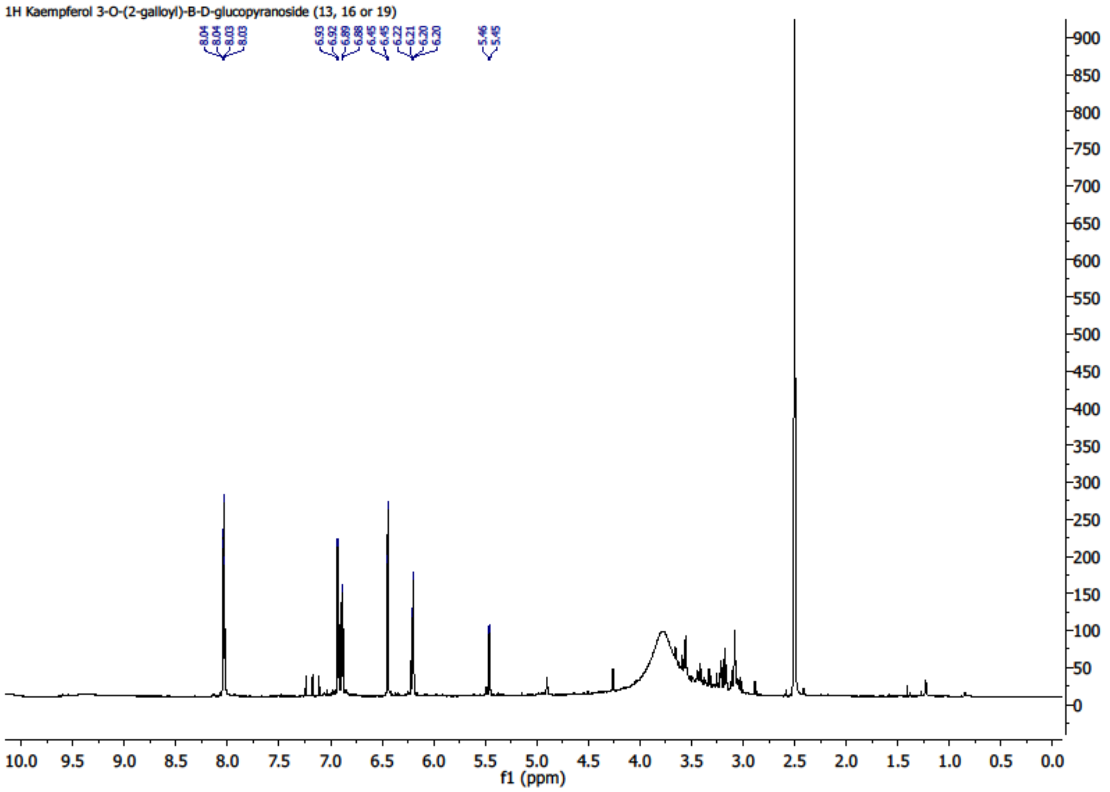

Figure S2. ^{1}H NMR spectrum (DMSO-*d*6) of kaempferol 3-*O*-(2′′-galloyl)-β-D-glucopyranoside (**13**, **16** or **19**).

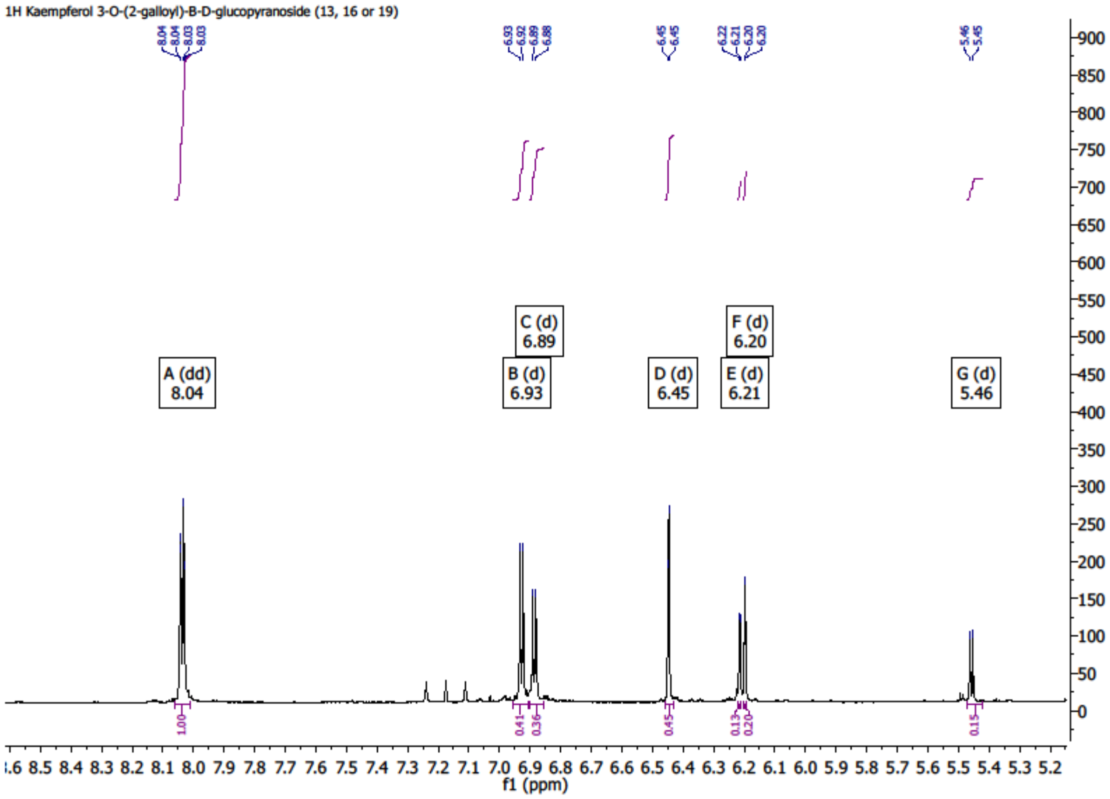

Figure S3. Zoom from 5.2 to 8.5 ppm, ^{1}H NMR spectrum (DMSO-*d*6) of kaempferol 3-*O*-(2′′-galloyl)-β-D-glucopyranoside (**13**, **16** or **19**).

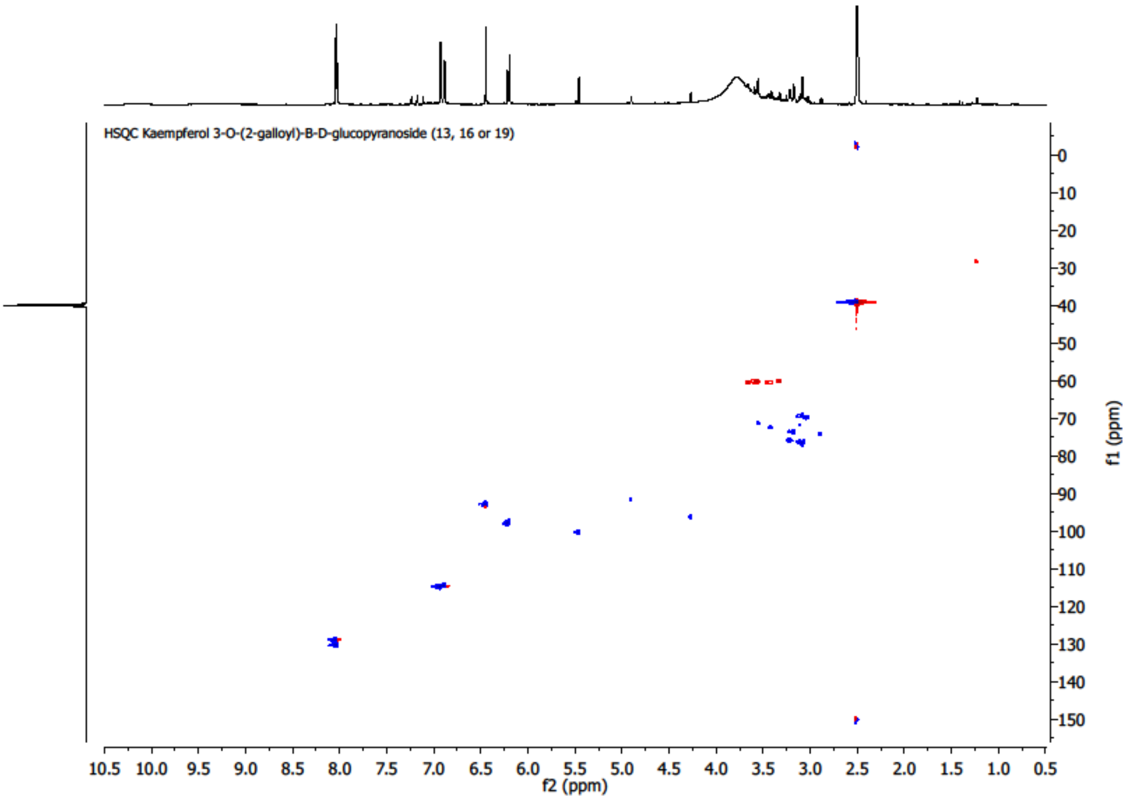

Figure S4. HSQC NMR spectrum (DMSO-*d*6) of kaempferol 3-*O*-(2′′-galloyl)-β-D-glucopyranoside (**13**, **16** or **19**).

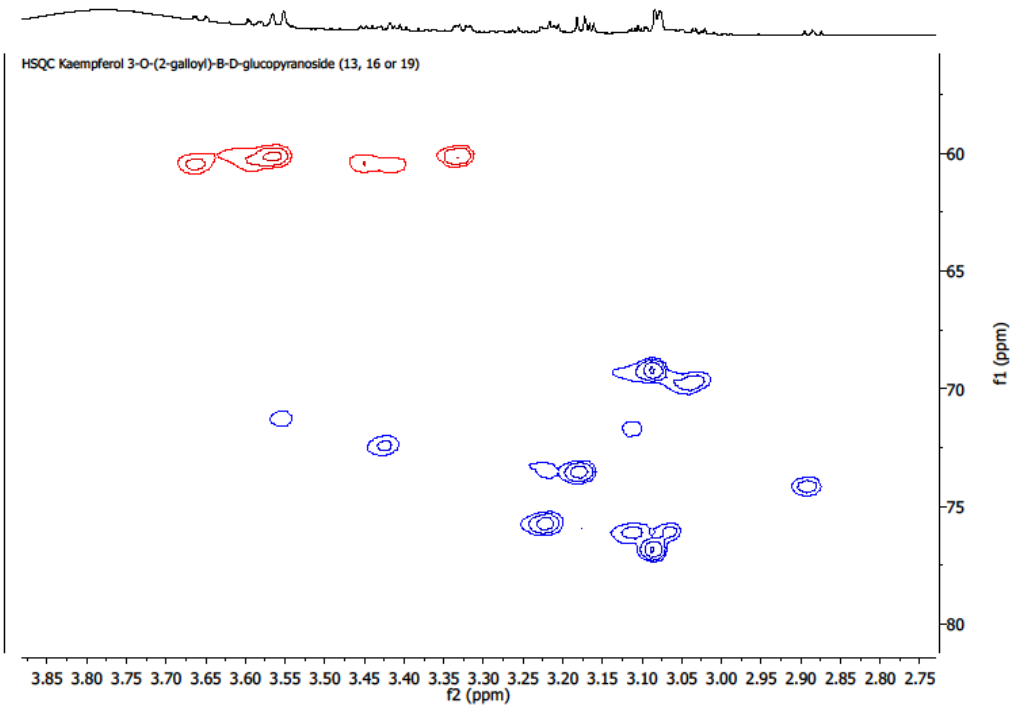

Figure S5. Zoom from 2.7 to 3.8 ppm, HSQC NMR spectrum (DMSO-*d*6) of kaempferol 3-*O*-(2′′-galloyl)-β-D-glucopyranoside (**13**, **16** or **19**).

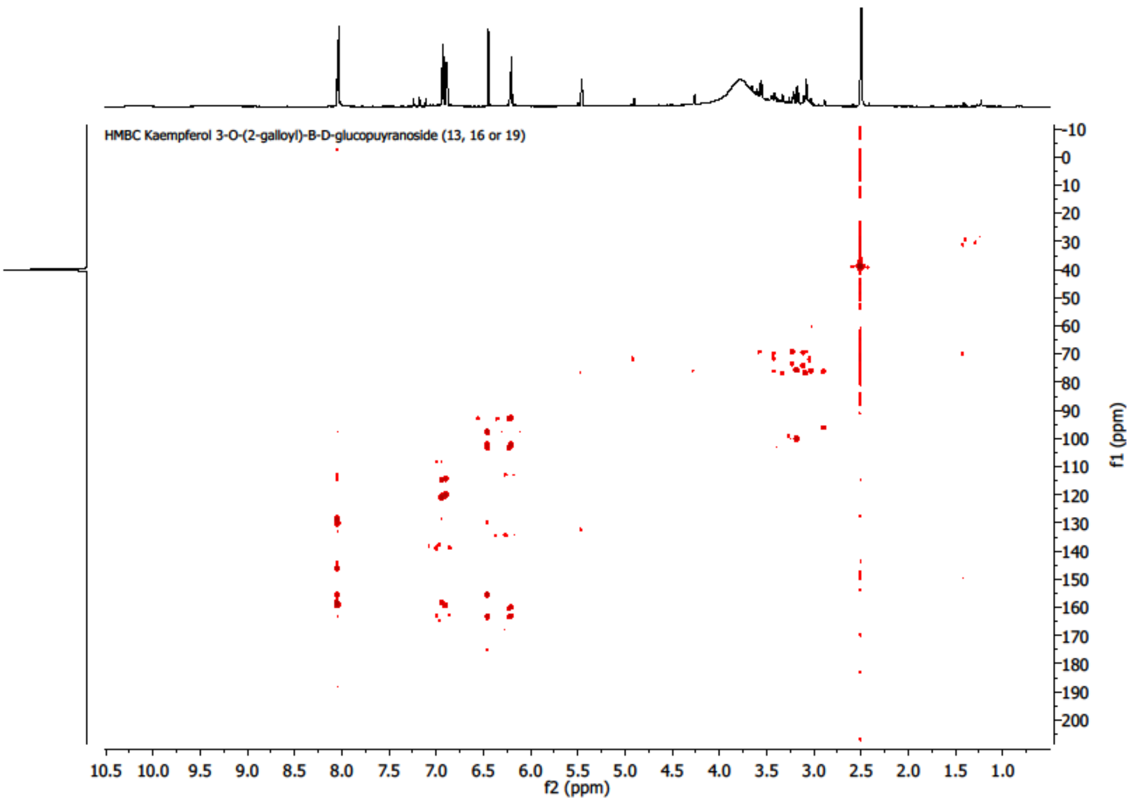

Figure S6. HMBC NMR spectrum (DMSO-*d*6) of kaempferol 3-*O*-(2′′-galloyl)-β-D-glucopyranoside (**13**, **16** or **19**).

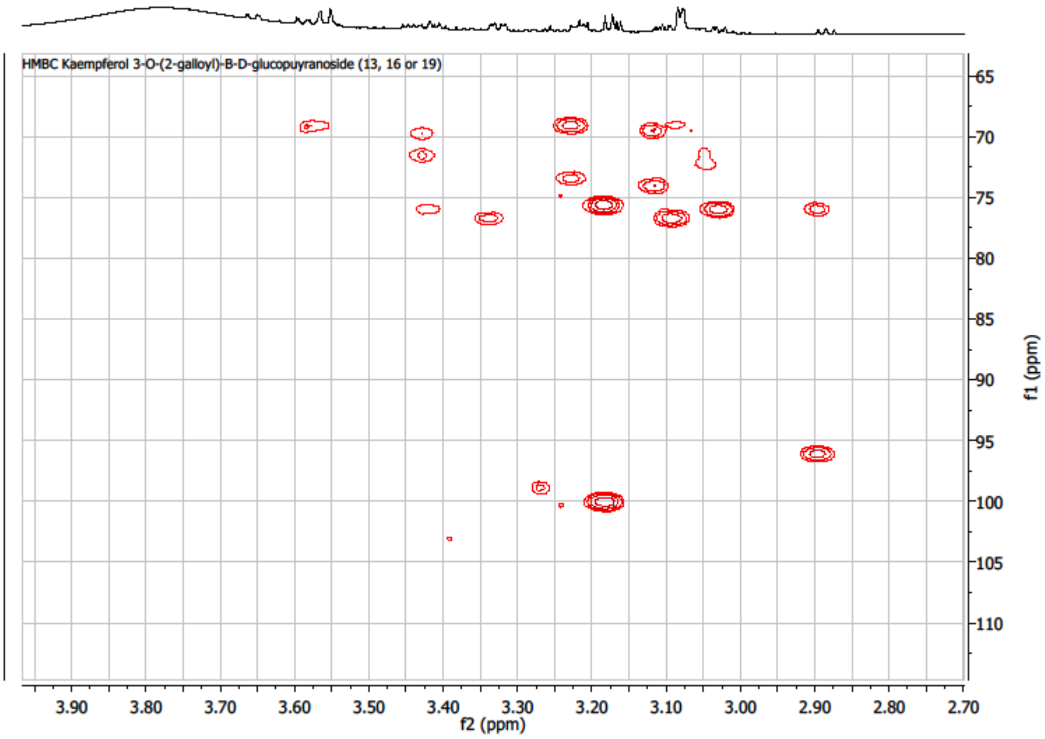

Figure S7. Zoom from 2.7 to 3.9 ppm, HMBC NMR spectrum (DMSO-*d*6) of kaempferol 3-*O*-(2′′-galloyl)-β-D-glucopyranoside (**13**, **16** or **19**).

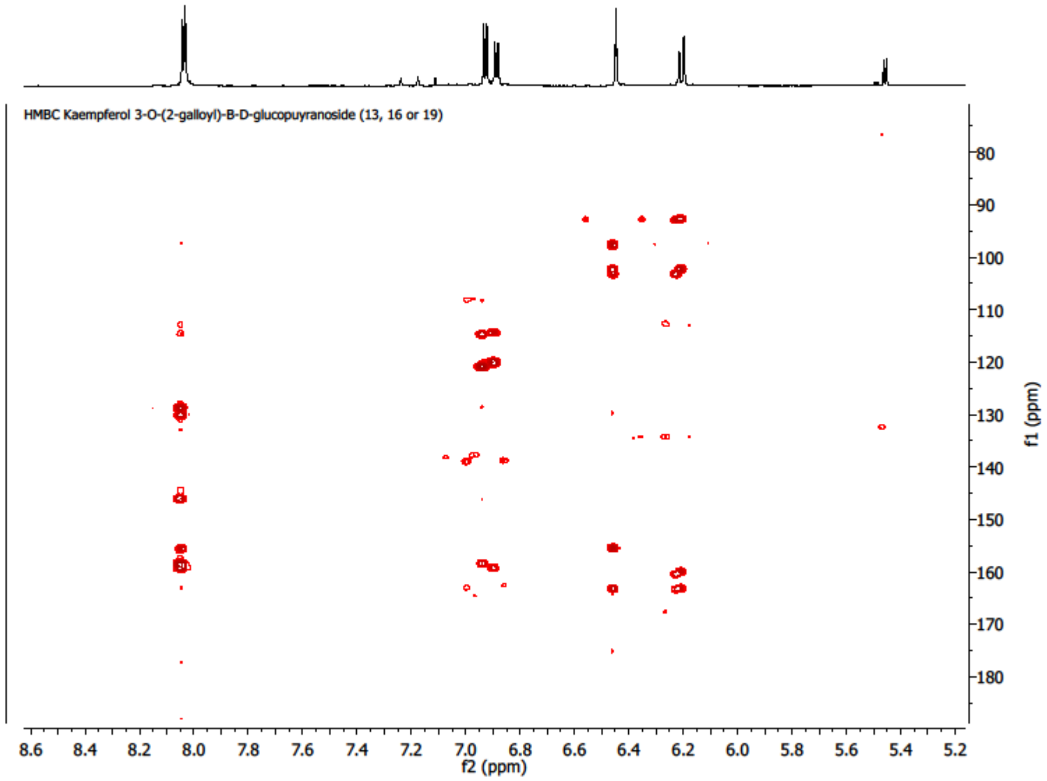

Figure S8. Zoom from 5.2 to 8.6 ppm, HMBC NMR spectrum (DMSO-*d*6) of kaempferol 3-*O*-(2′′-galloyl)-β-D-glucopyranoside (**13**, **16** or **19**).

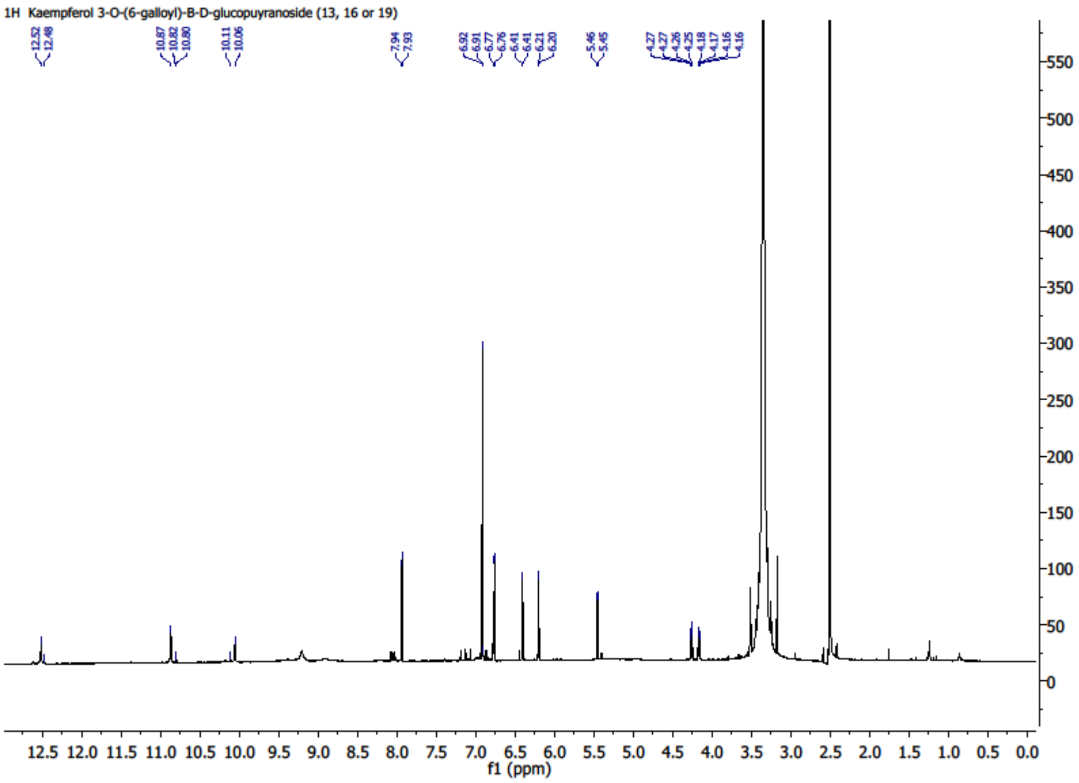

Figure S9. ^{1}H NMR spectrum (DMSO-*d*6) of kaempferol 3-*O*-(6′′-galloyl)-β-D-glucopyranoside (**13**, **16** or **19**).

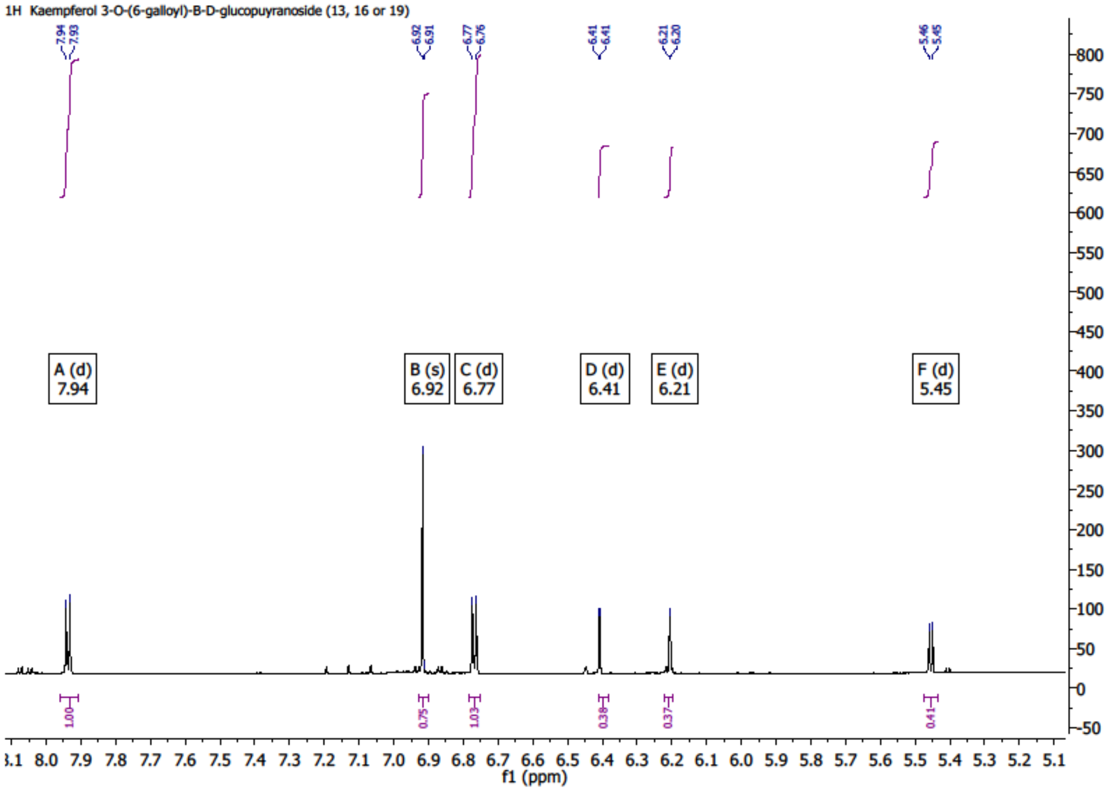

Figure S10. Zoom from 5.1 to 8.1 ppm, ^{1}H NMR spectrum (DMSO-*d*6) of kaempferol 3-*O*-(6′′-galloyl)-β-D-glucopyranoside (**13**, **16** or **19**).

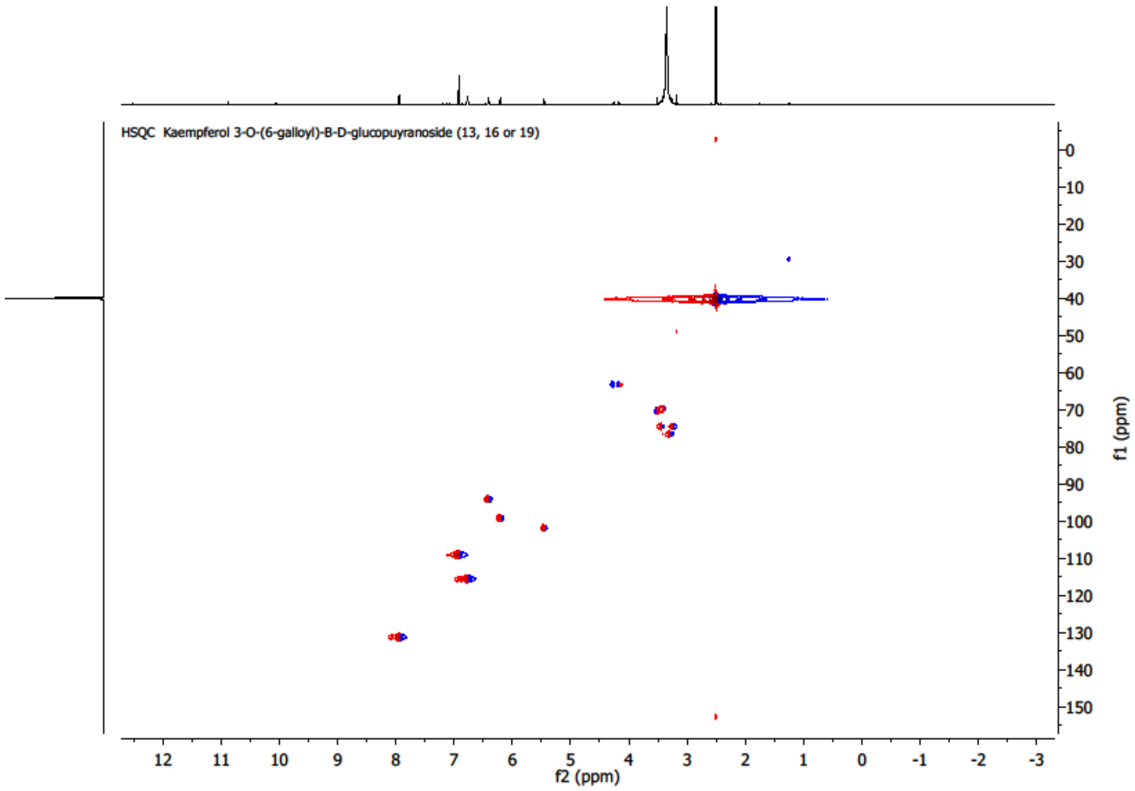

Figure S11. HSQC NMR spectrum (DMSO-*d*6) of kaempferol 3-*O*-(6′′-galloyl)-β-D-glucopyranoside (**13**, **16** or **19**).

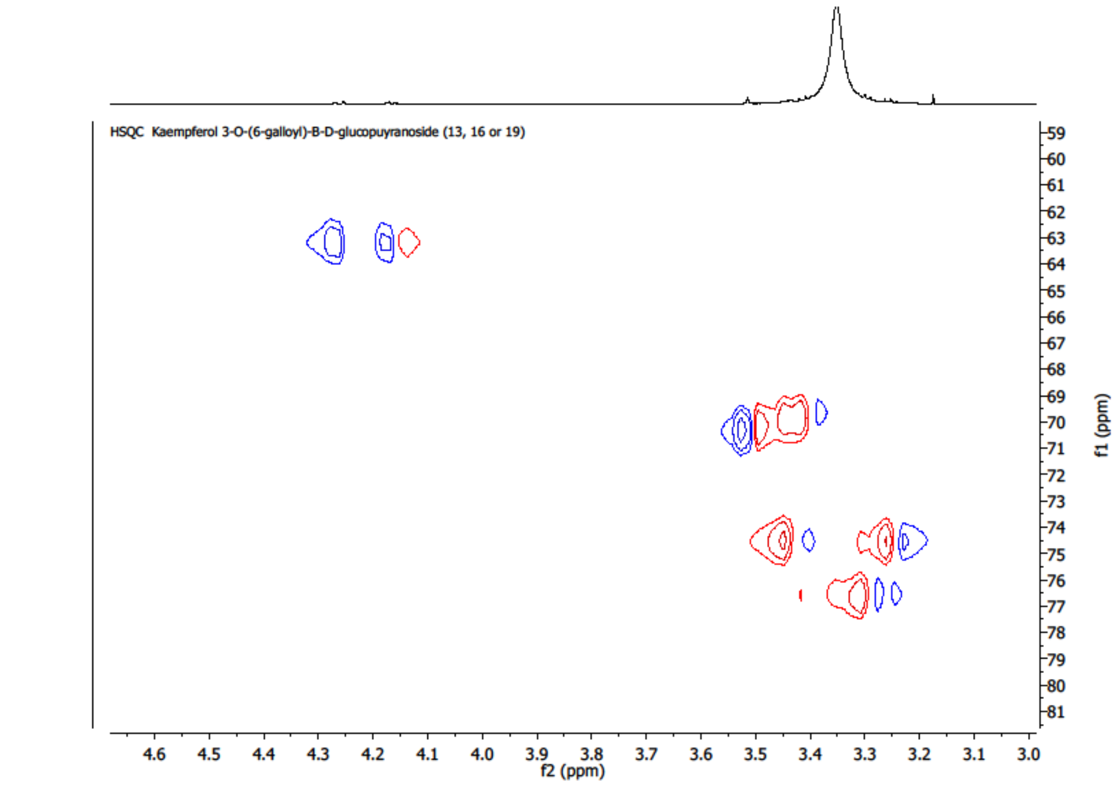

Figure S12. Zoom from 3.0 to 4.6 ppm, HSQC NMR spectrum (DMSO-*d*6) of kaempferol 3-*O*-(6′′-galloyl)-β-D-glucopyranoside (**13**, **16** or **19**).

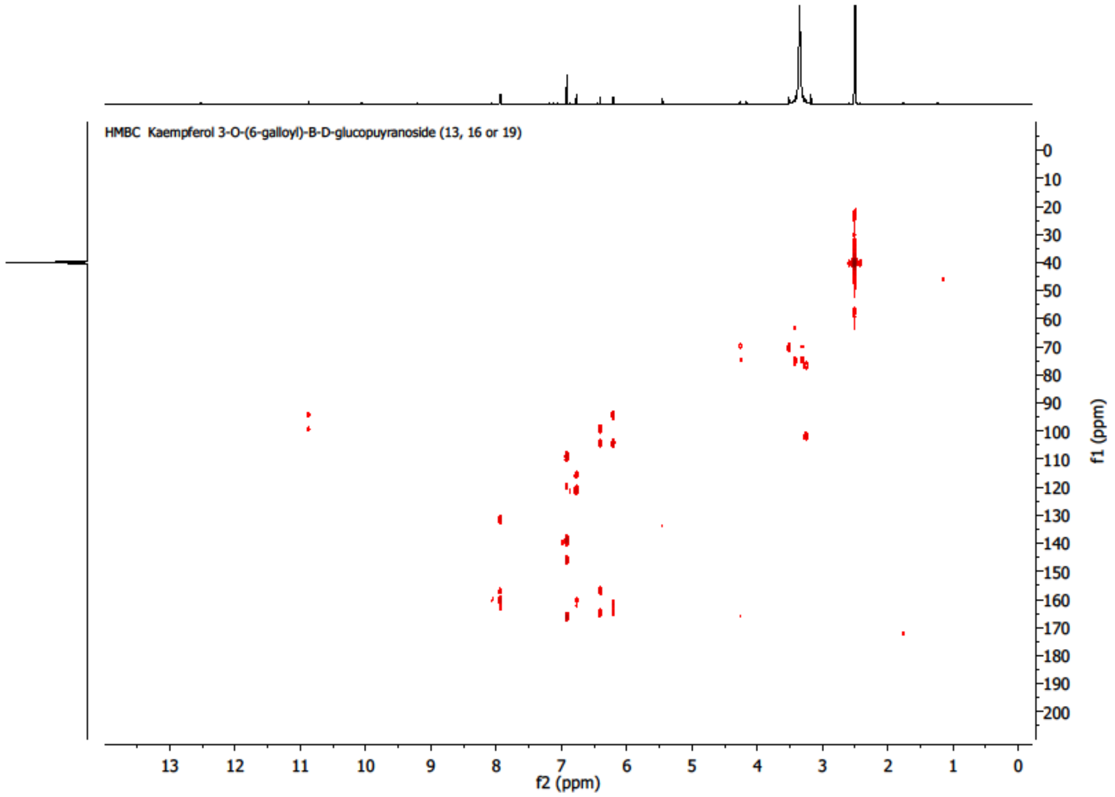

Figure S13. HMBC NMR spectrum (DMSO-d6) of kaempferol 3-O-(6′′-galloyl)-β-D-glucopyranoside (**13**, **16** or **19**).

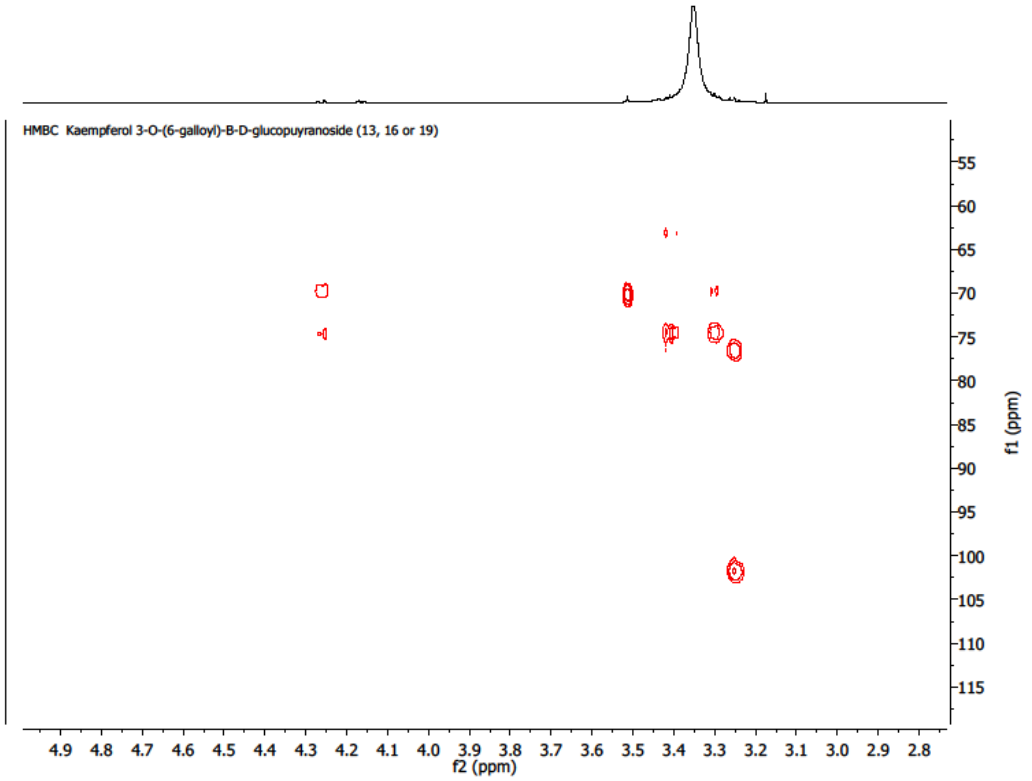

Figure S14. Zoom from 2.8 to 4.9 ppm, HMBC NMR spectrum (DMSO-*d*6) of kaempferol 3-*O*-(6′′-galloyl)-β-D-glucopyranoside (**13**, **16** or **19**).

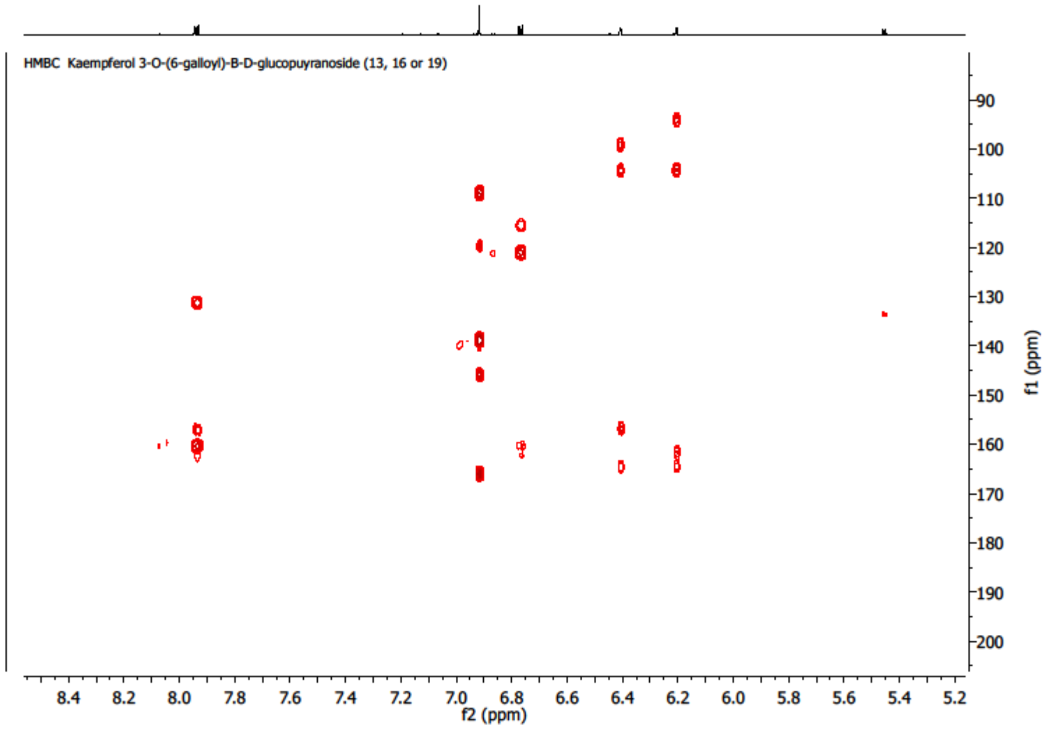

Figure S15. Zoom from 5.2 to 8.4 ppm, HMBC NMR spectrum (DMSO-*d*6) of kaempferol 3-*O*-(6′′-galloyl)-β-D-glucopyranoside (**13**, **16** or **19**).

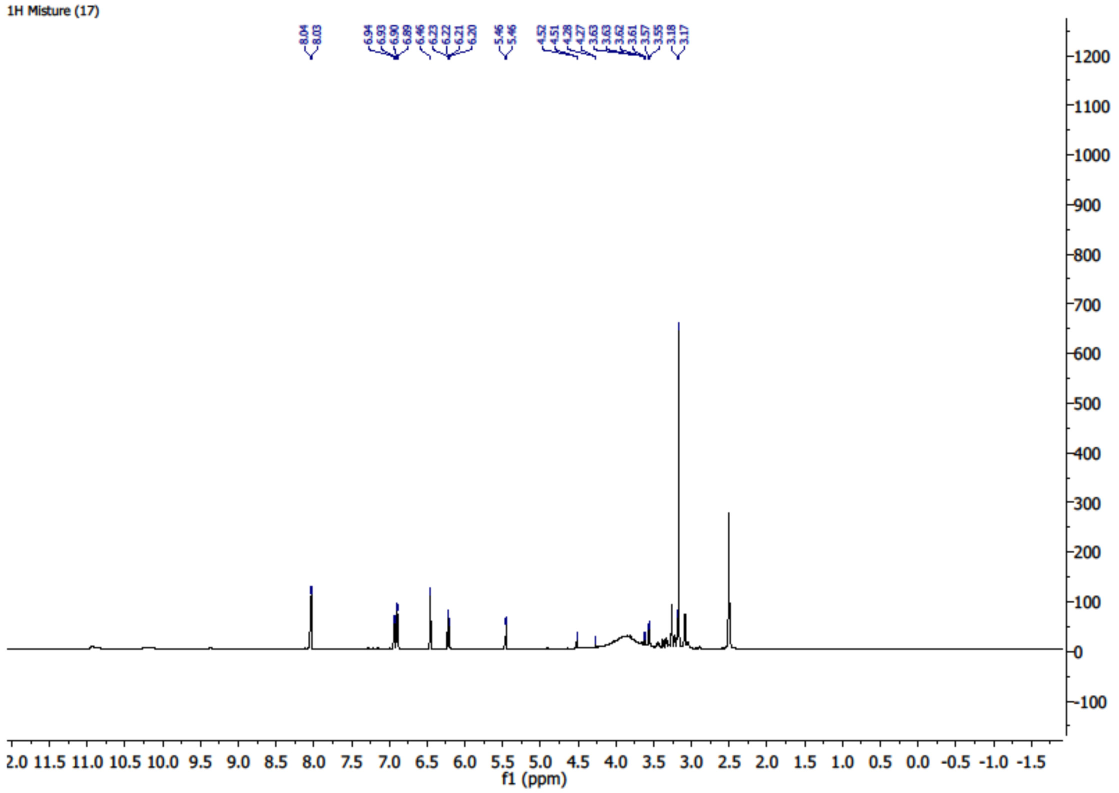

Figure S16. ^{1}H NMR spectrum (DMSO-*d*6) of mixture (**17**).

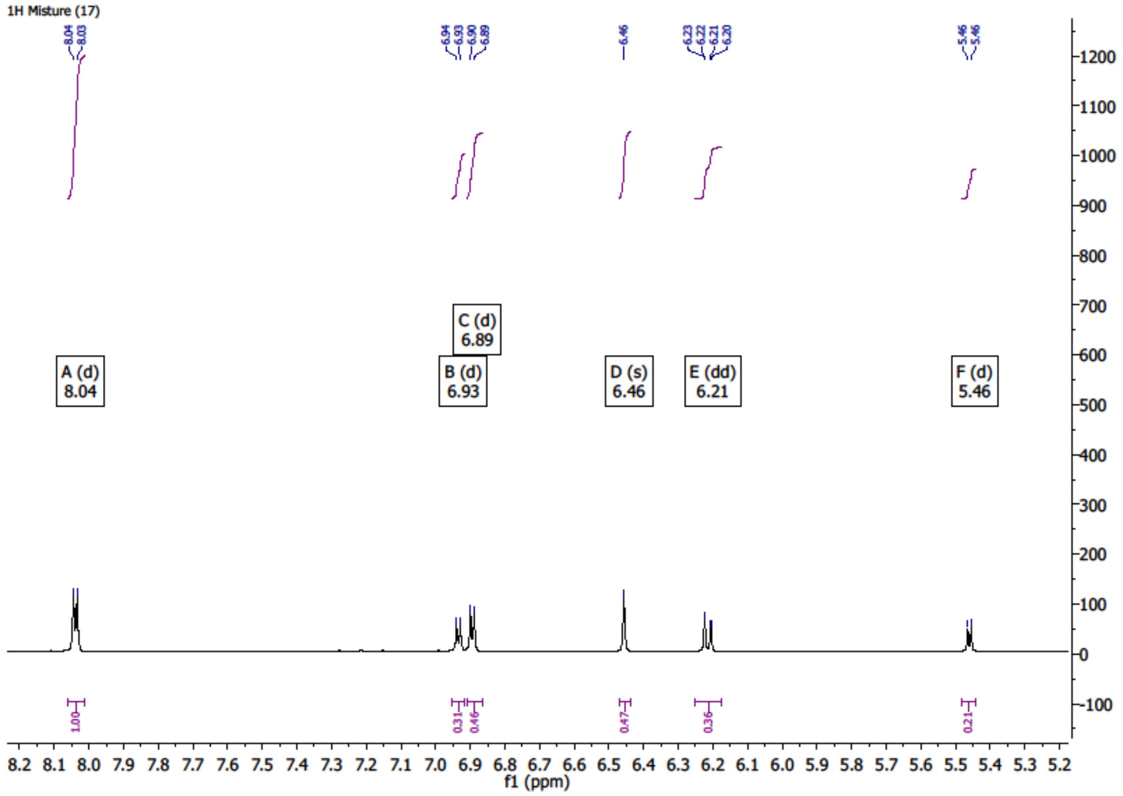

Figure S17. Zoom from 5.2 to 8.2 ppm, ^{1}H NMR spectrum (DMSO-*d*6) of mixture (**17**).

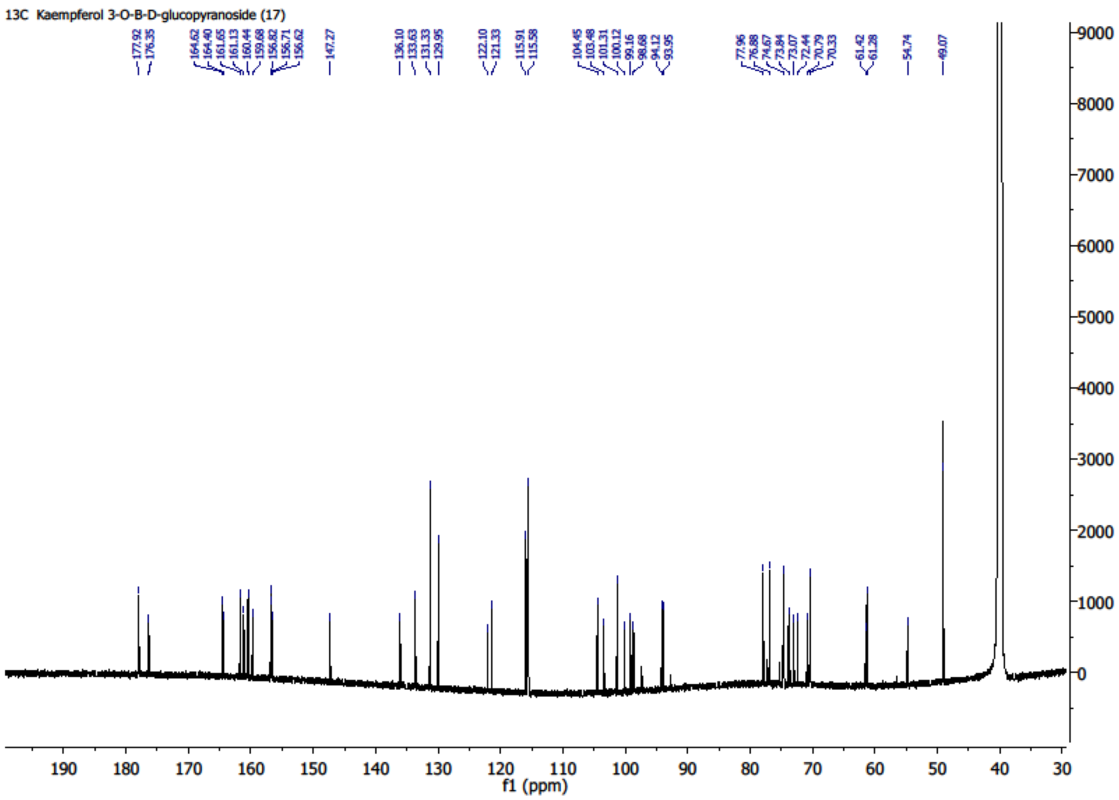

Figure S18. ^{13}C NMR spectrum (DMSO-*d*6) of mixture (**17**).

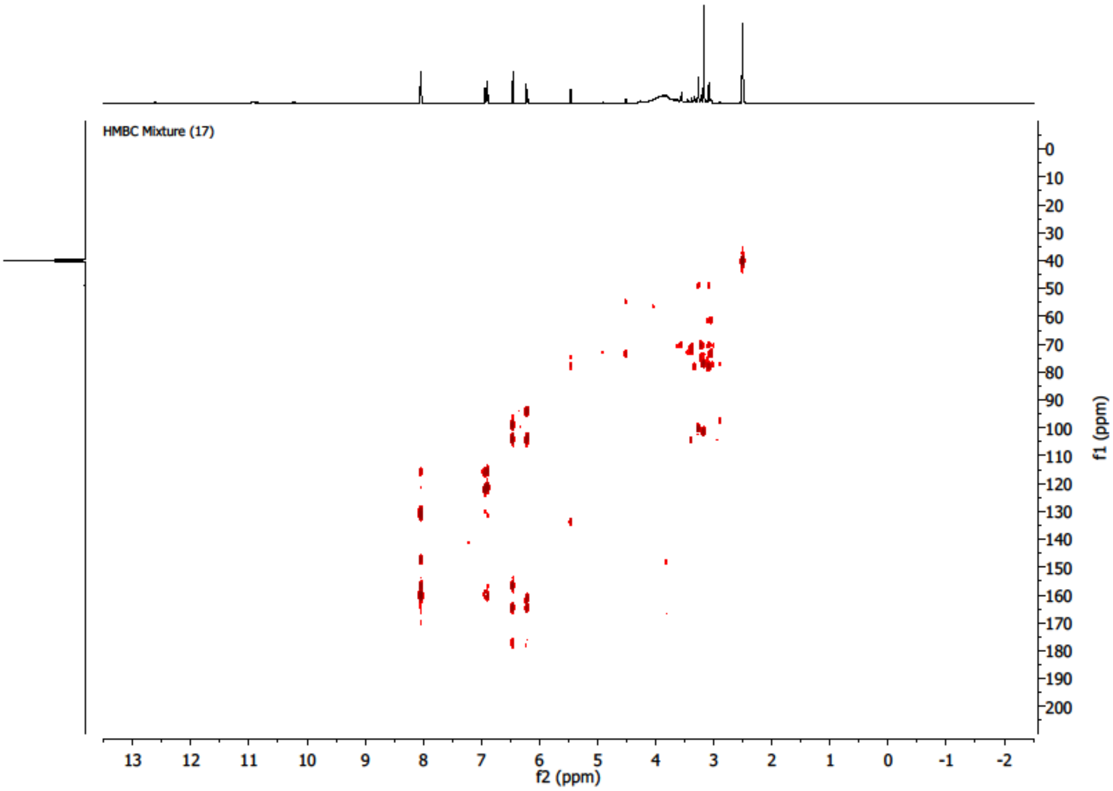

Figure S19. HMBC NMR spectrum (DMSO-*d*6) of mixture (**17**).

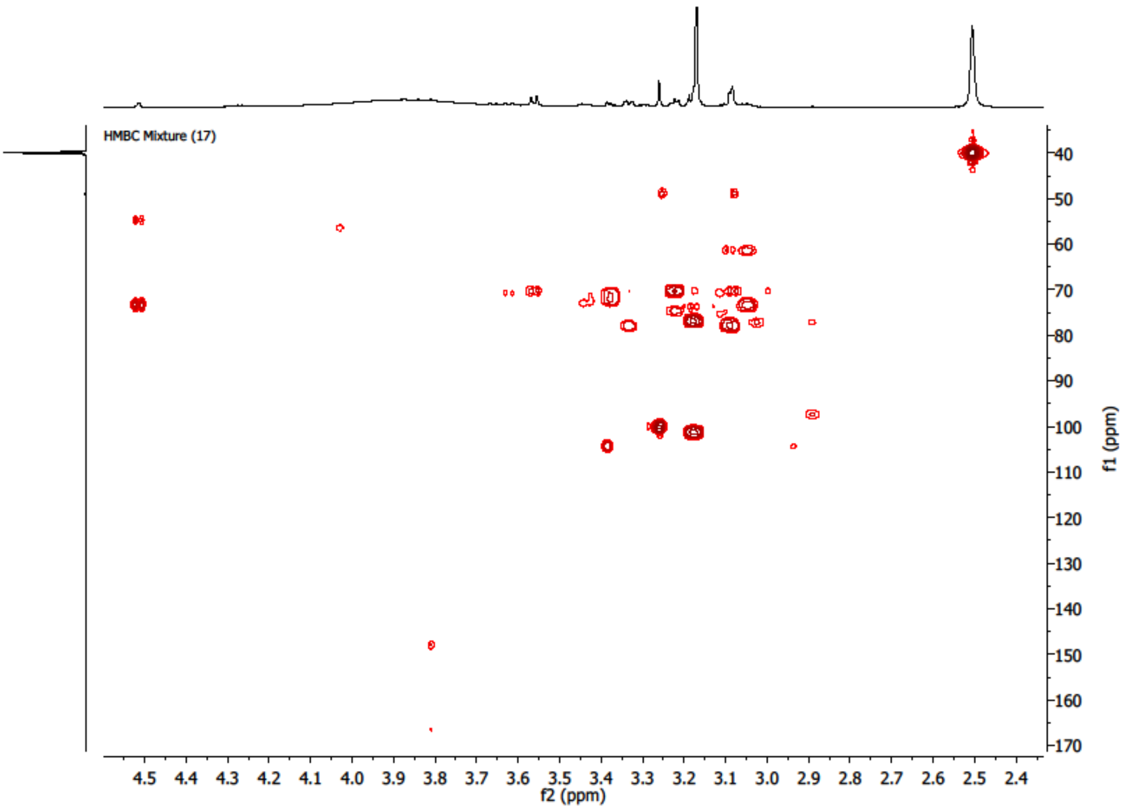

Figure S20. Zoom from 2.4 to 4.5 ppm, HMBC NMR spectrum (DMSO-*d*6) of mixture (**17**).

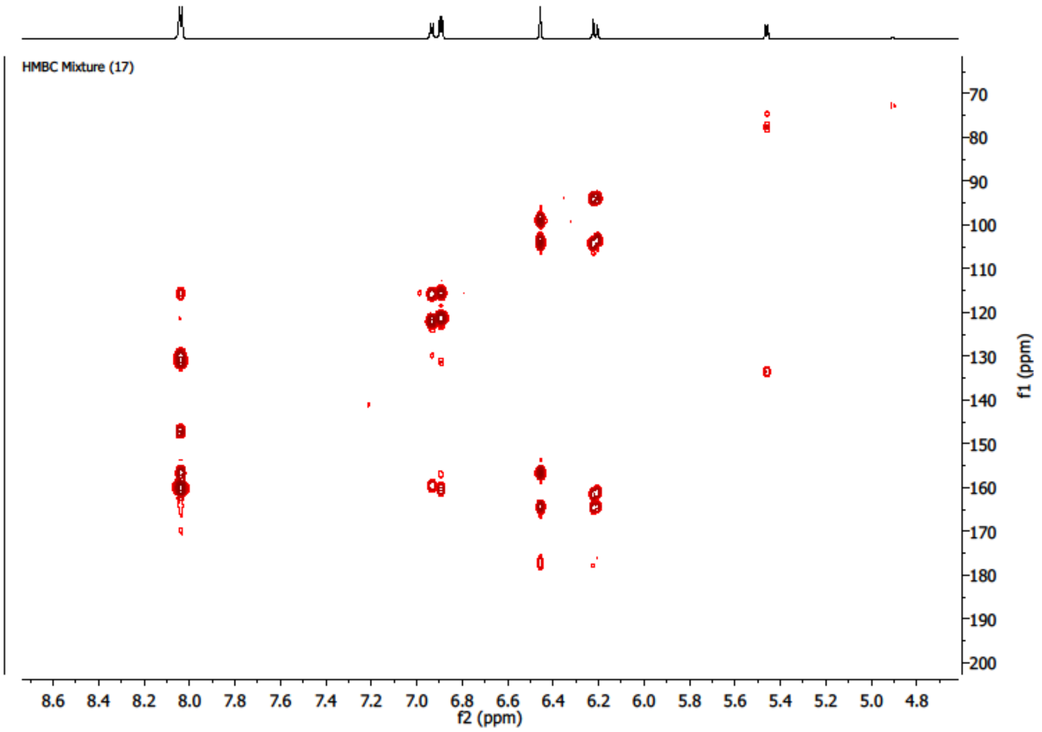

Figure S21. Zoom from 4.8 to 8.6 ppm, HMBC NMR spectrum (DMSO-*d*6) of mixture (**17**).

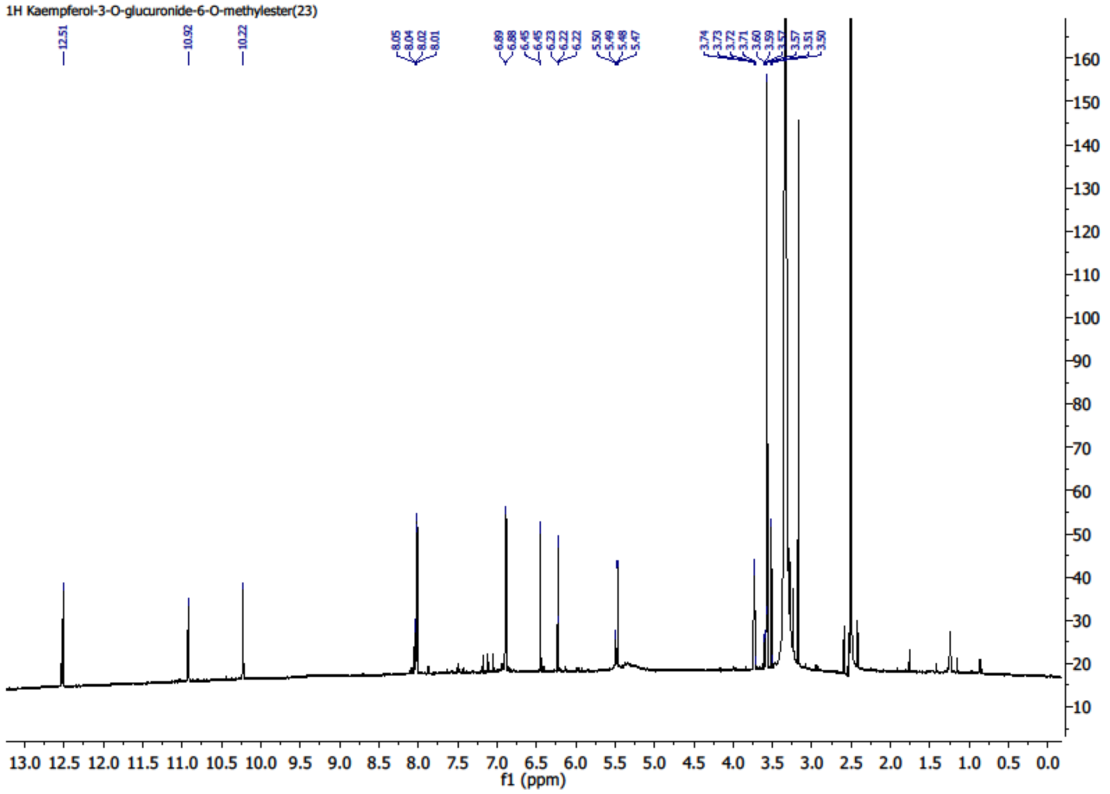

Figure S22. ^{1}H NMR spectrum (DMSO-*d*6) of kaempferol 3-*O*-glucoronide-6′′-*O*-methylester (**23**).

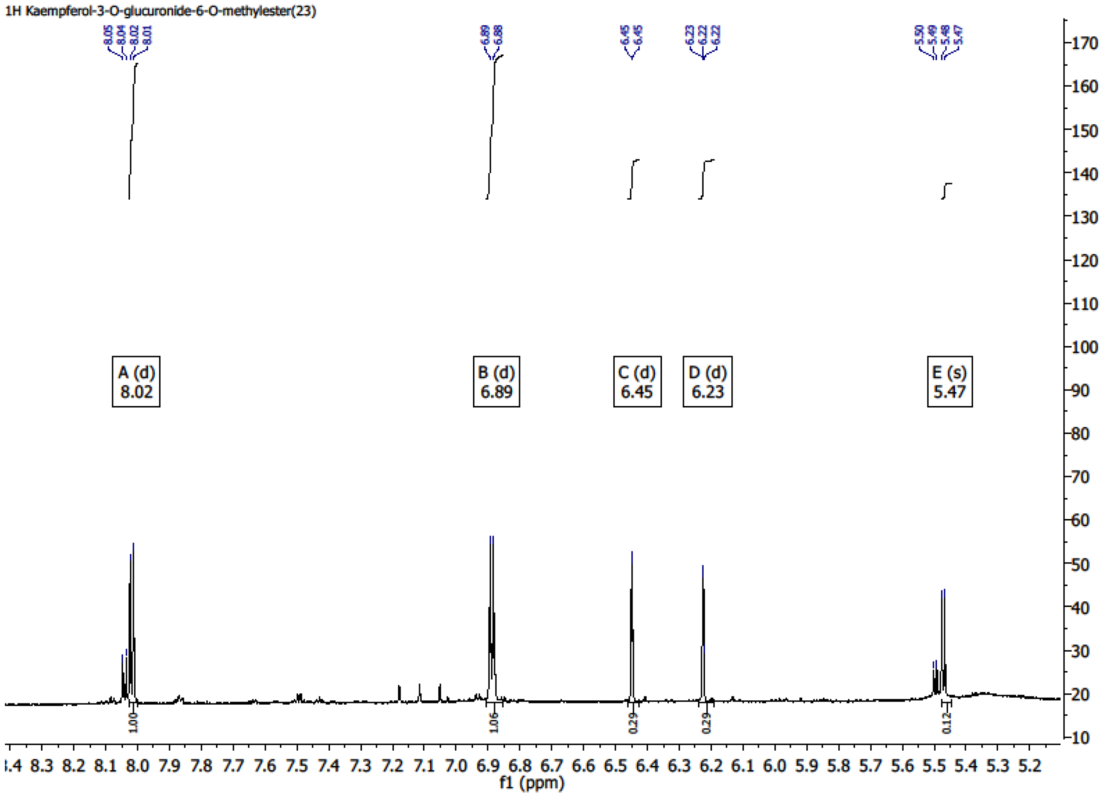

Figure S23. Zoom from 5.1 to 8.1 ppm, ^{1}H NMR spectrum (DMSO-*d*6) of kaempferol 3-*O*-glucoronide-6′′-*O*-methylester (**23**).

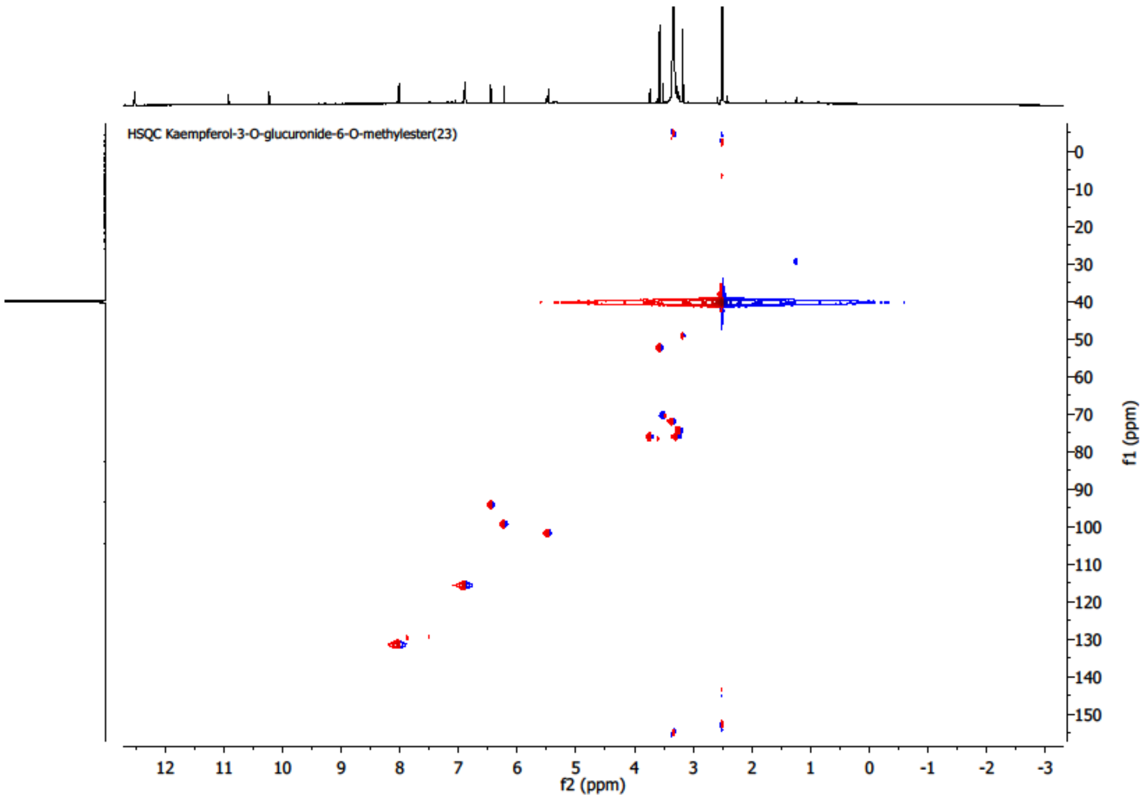

Figure S24. HSQC NMR spectrum (DMSO-*d*6) of kaempferol 3-*O*-glucoronide-6′′-*O*-methylester (**23**).

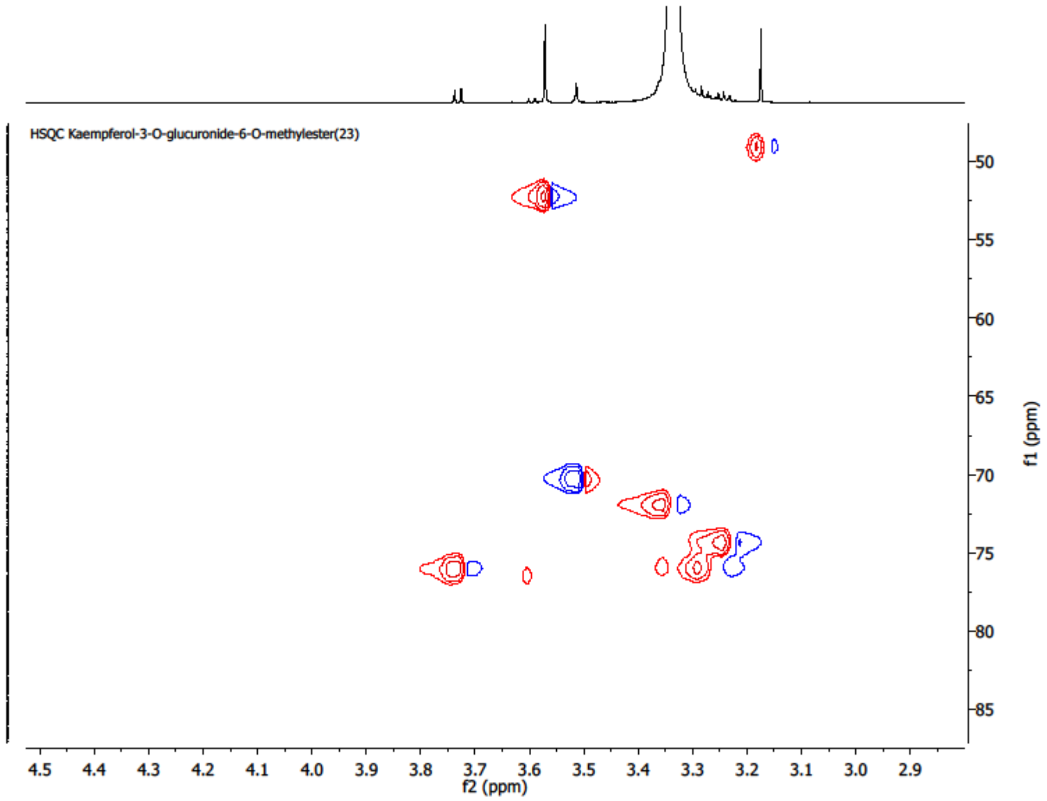

Figure S25. Zoom from 2.9 to 4.5 ppm, HSQC NMR spectrum (DMSO-*d*6) of kaempferol 3-*O*-glucoronide-6′′-*O*-methylester (**23**).

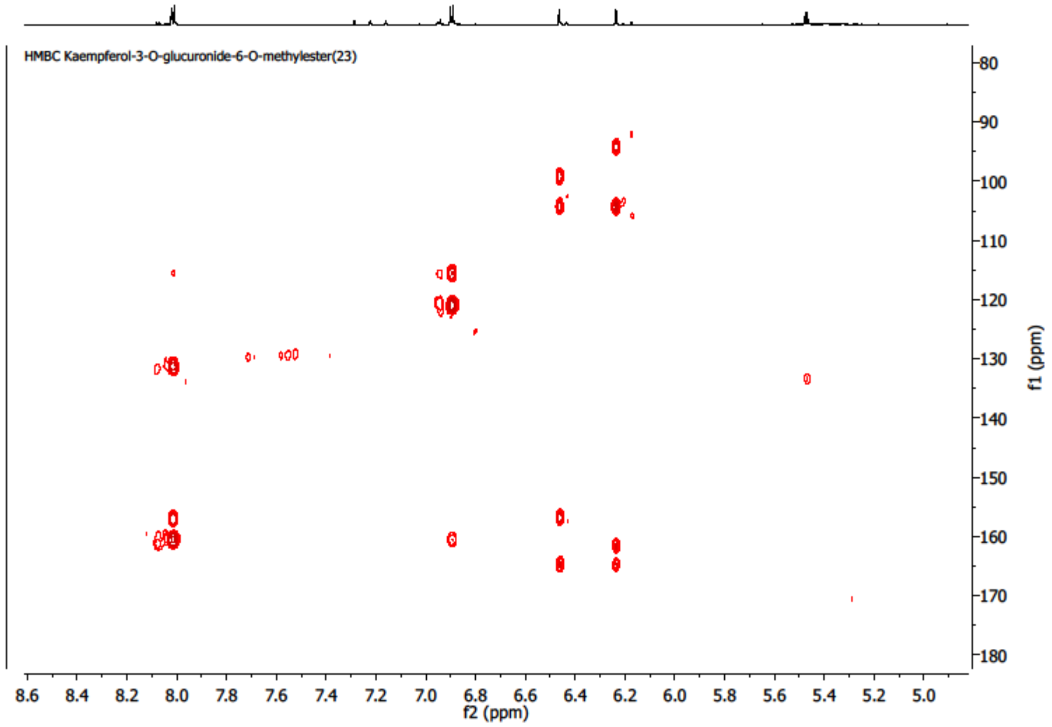

Figure S26. Zoom from 5.0 to 8.6 ppm, HMBC NMR spectrum (DMSO-*d*6) of kaempferol 3-*O*-glucoronide-6′′-*O*-methylester (**23**).

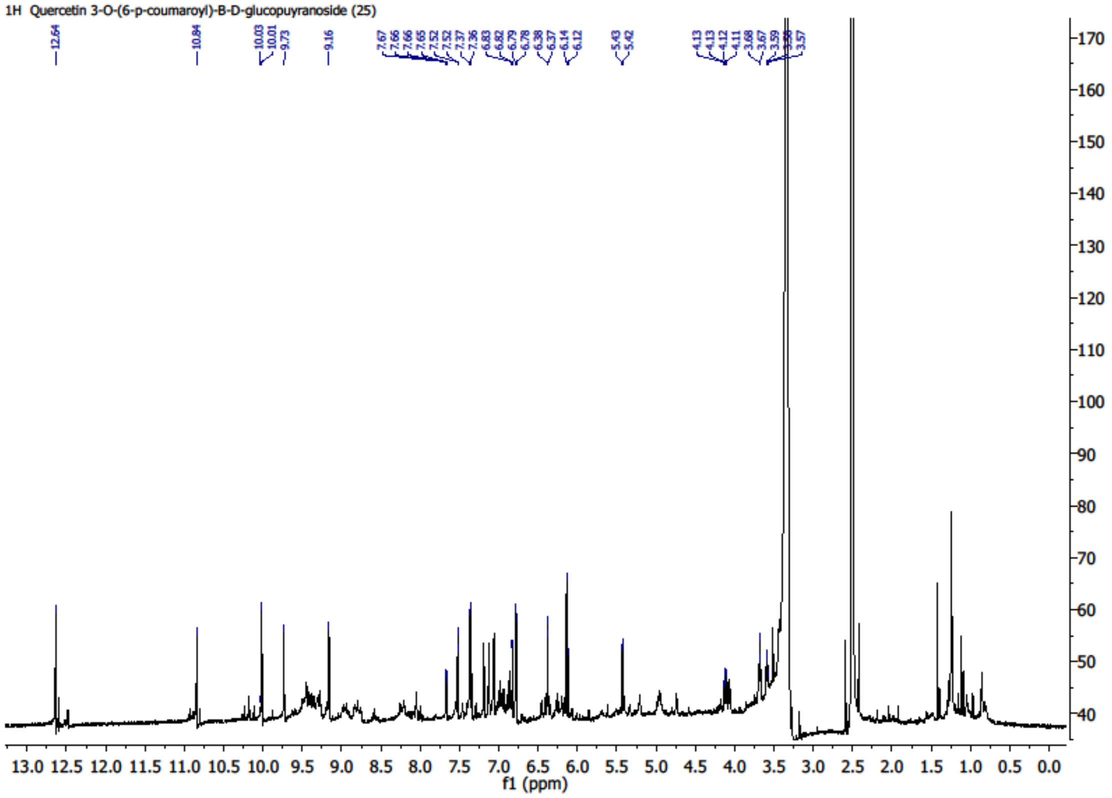

Figure S27. ^{1}H NMR spectrum (DMSO-*d*6) of Quercetin 3-*O*-(6′′-*p*-coumaroyl)-β-D-glucopyranoside (**25**).

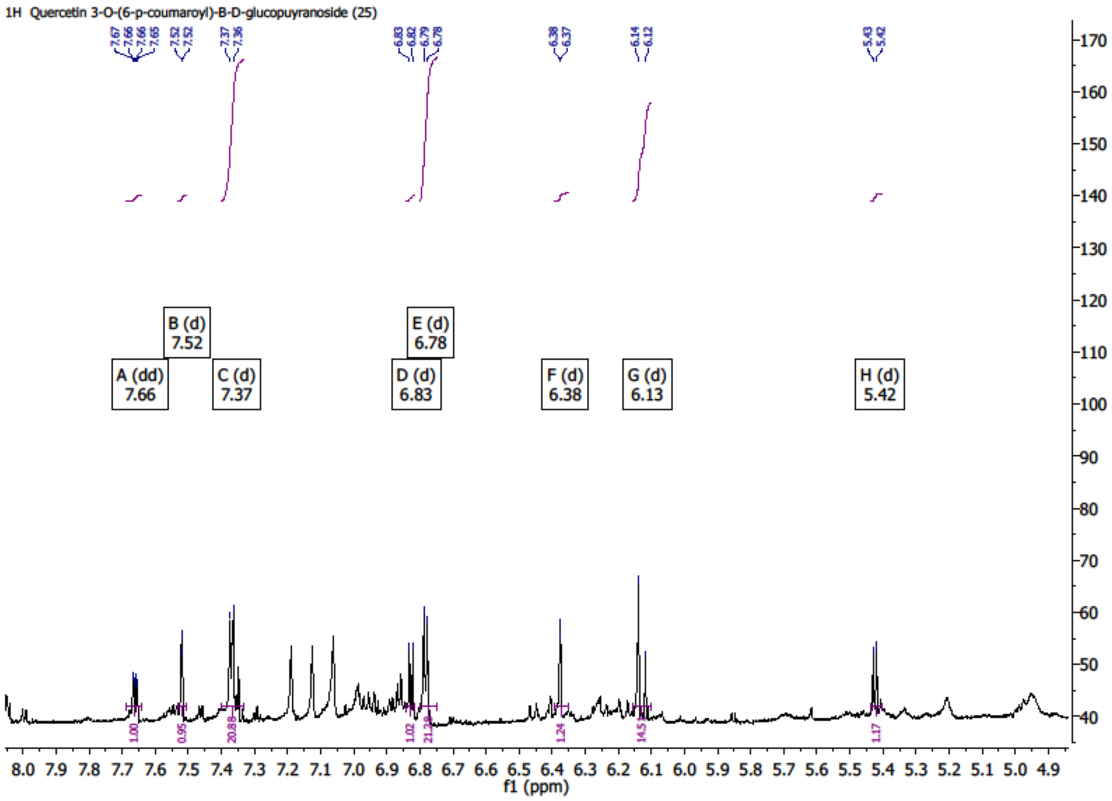

Figure S28. Zoom from 5.1 to 8.1 ppm, ^{1}H NMR spectrum (DMSO-*d*6) of Quercetin 3-*O*-(6′′-*p*-coumaroyl)-β-D-glucopyranoside (**25**).

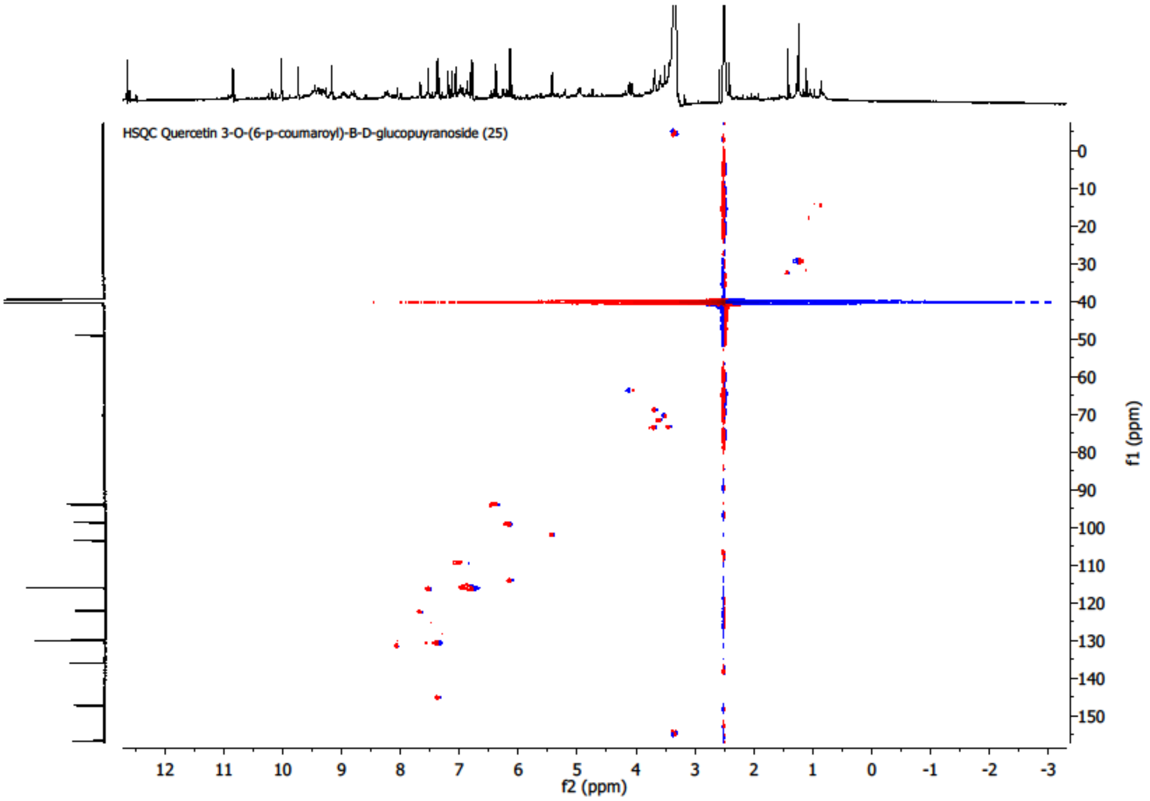

Figure S29. HSQC NMR spectrum (DMSO-*d*6) of Quercetin 3-*O*-(6′′-*p*-coumaroyl)-β-D-glucopyranoside (**25**).

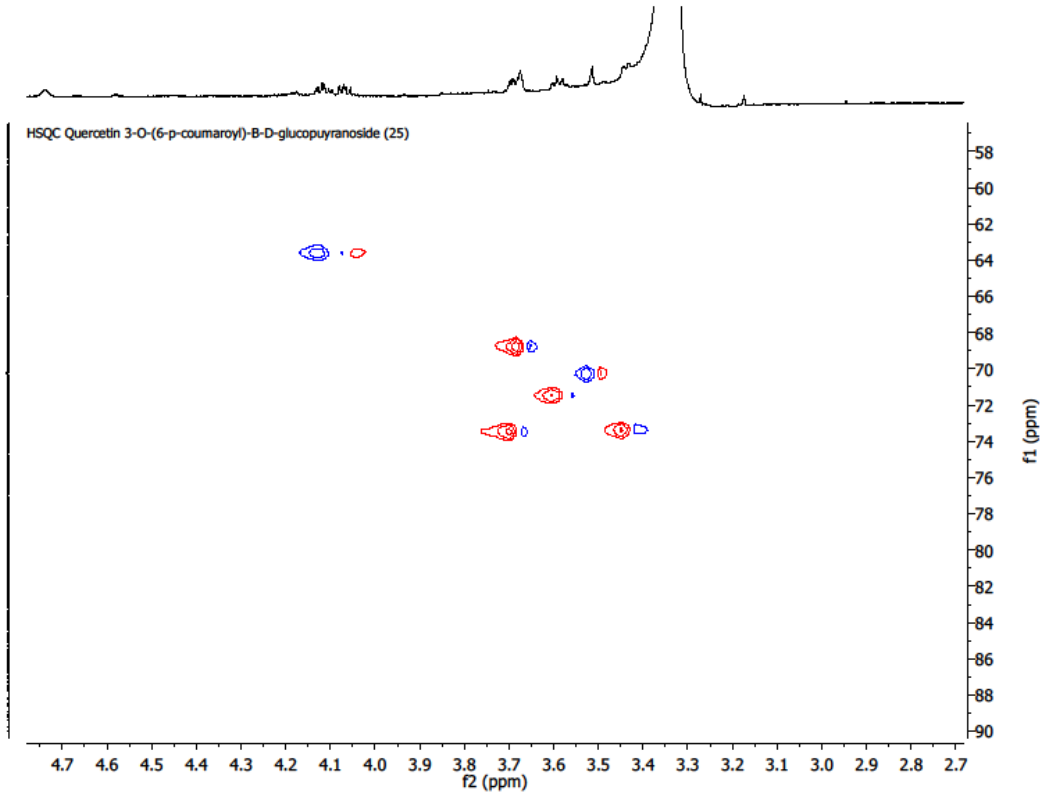

Figure S30. Zoom from 2.7 to 4.7 ppm, HSQC NMR spectrum (DMSO-*d*6) of Quercetin 3-*O*-(6′′-*p*-coumaroyl)-β-D-glucopyranoside (**25**).

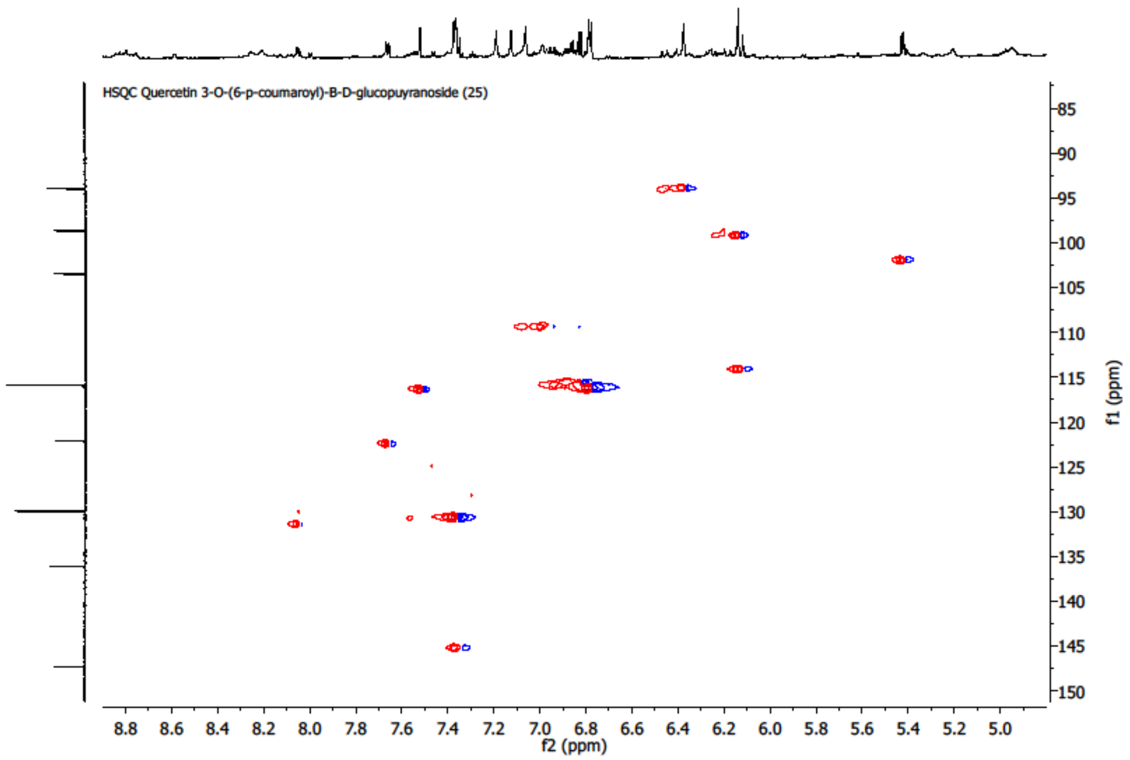

Figure S31. Zoom from 5.0 to 8.8 ppm, HSQC NMR spectrum (DMSO-*d*6) of Quercetin 3-*O*-(6′′-*p*-coumaroyl)-β-D-glucopyranoside (**25**).

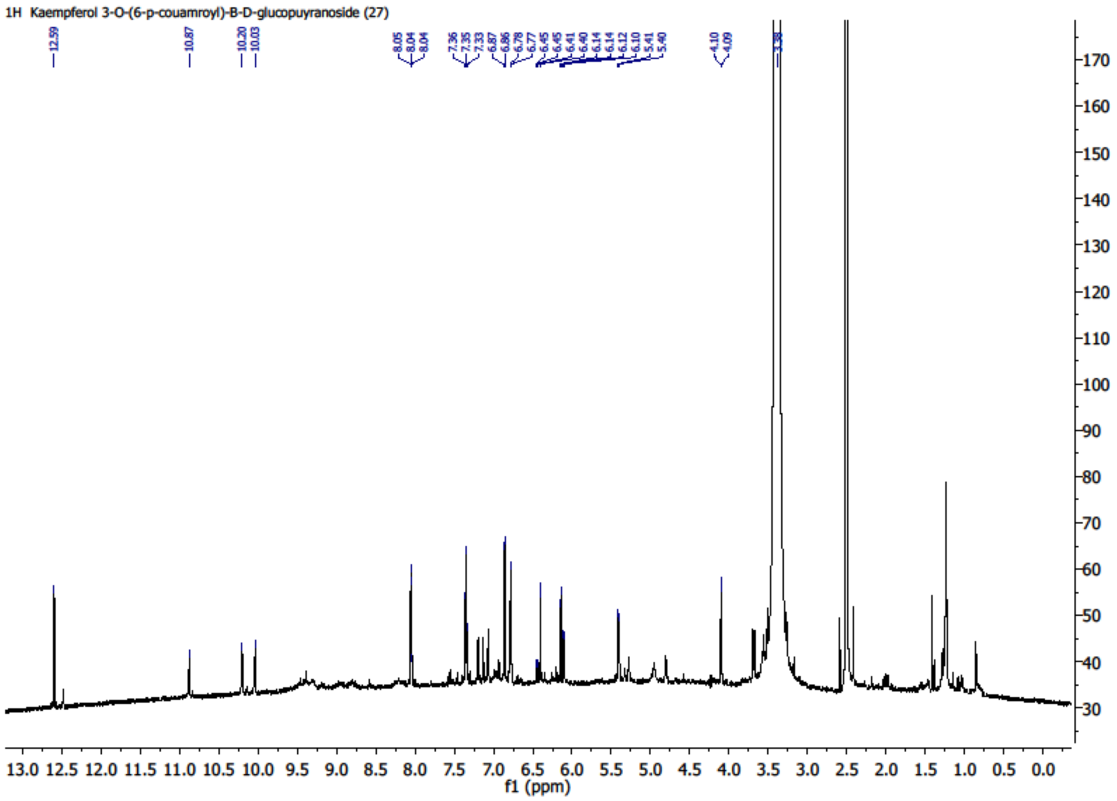

Figure S32. ^{1}H NMR spectrum (DMSO-*d*6) of Kaempferol 3-*O*-(6′′-*p*-coumaroyl)-β-D-glucopyranoside (**27**).

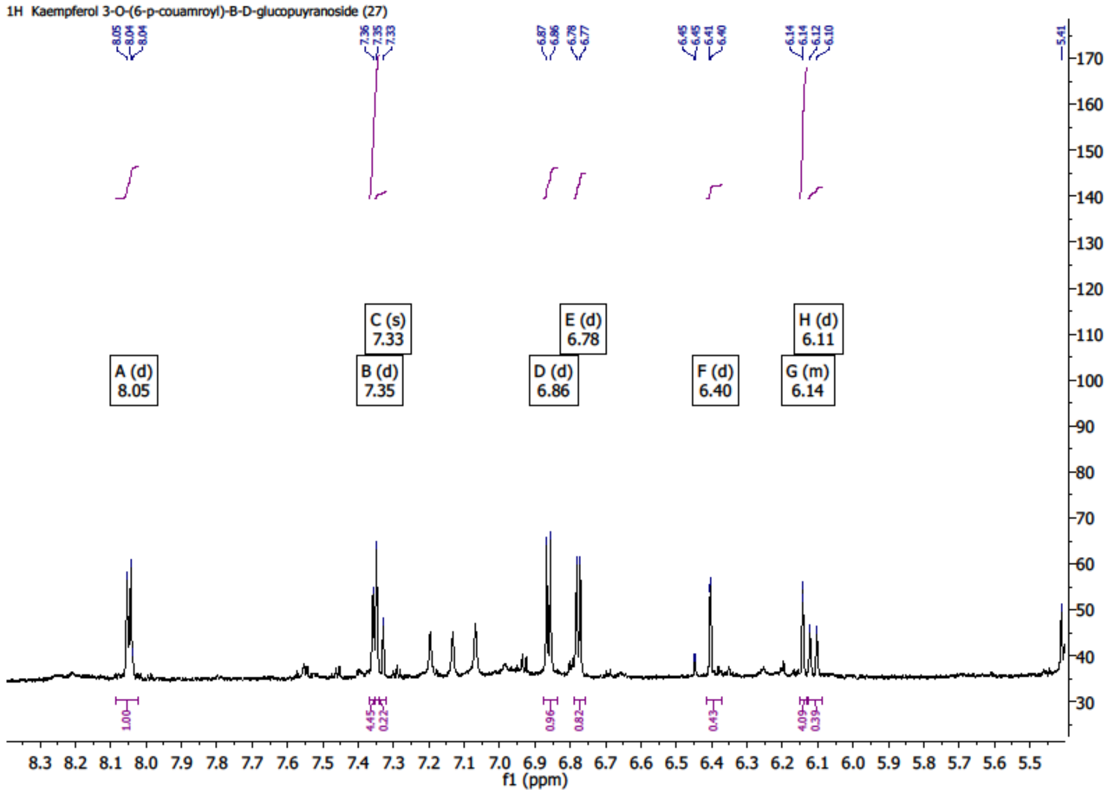

Figure S33. Zoom from 5.1 to 8.1 ppm, ^{1}H NMR spectrum (DMSO-*d*6) of Kaempferol 3-*O*-(6′′-*p*-coumaroyl)-β-D-glucopyranoside (**27**).

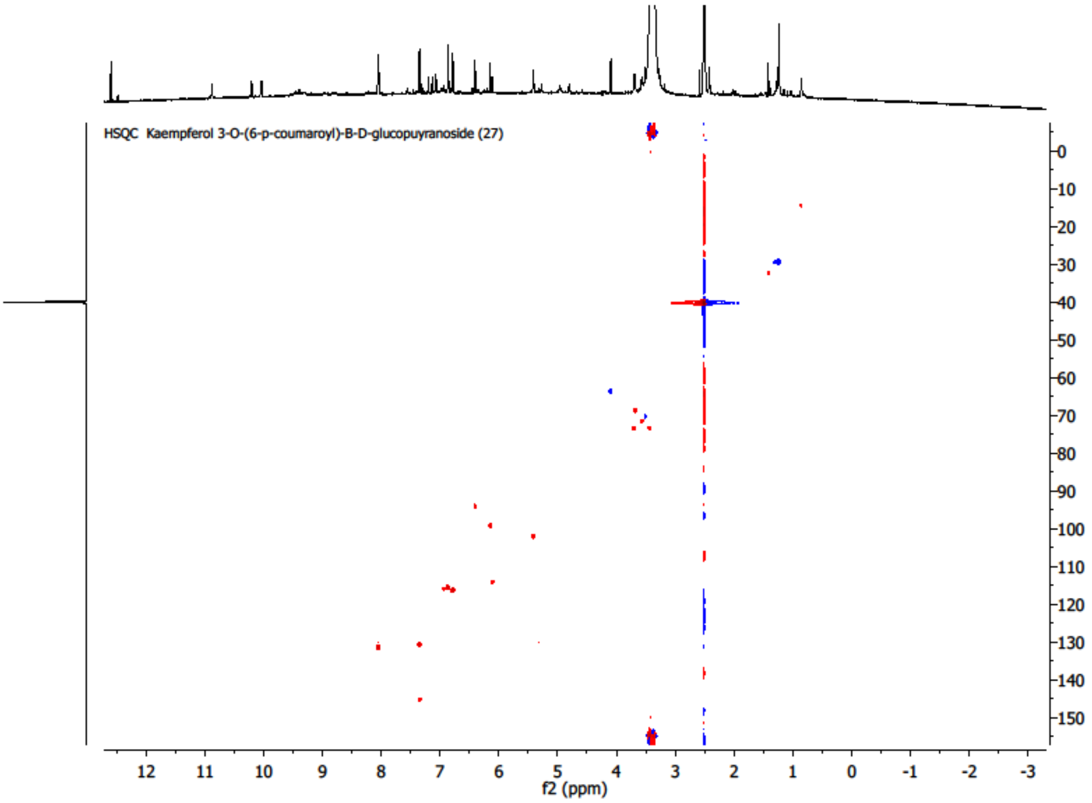

Figure S34. HSQC NMR spectrum (DMSO-*d6*) of Kaempferol 3-*O*-(6′′-*p*-coumaroyl)-β-D-glucopyranoside (**27**).

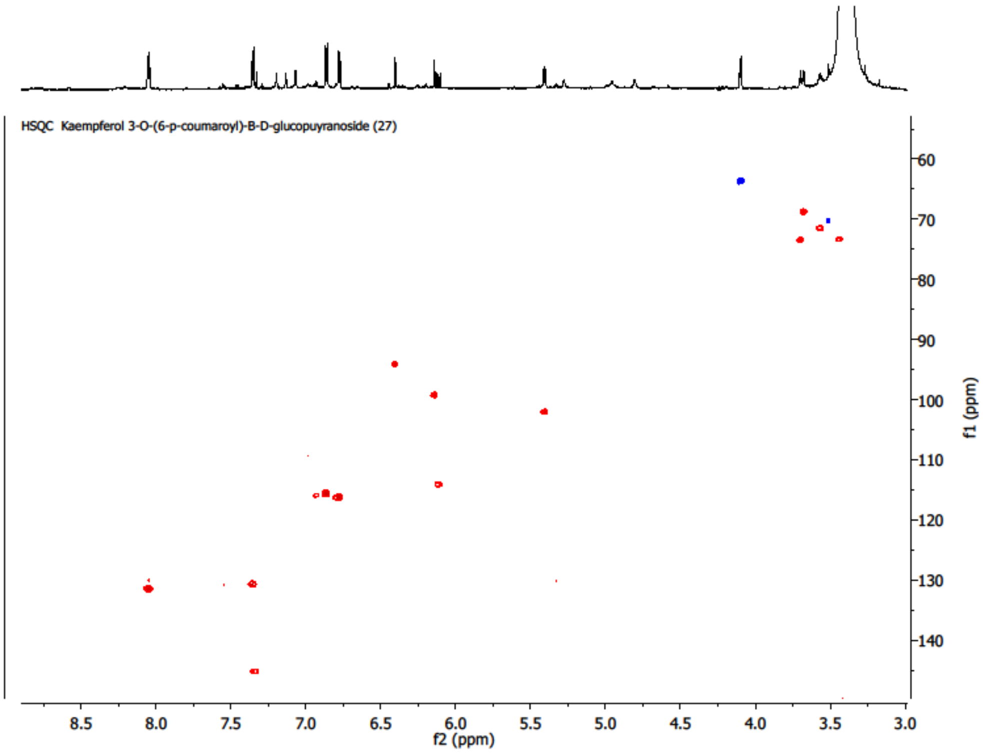

Figure S35. Zoom from 3.0 to 4.6 ppm, HSQC NMR spectrum (DMSO-*d6*) of Kaempferol 3-*O*-(6′′-*p*-coumaroyl)-β-D-glucopyranoside (**27**).

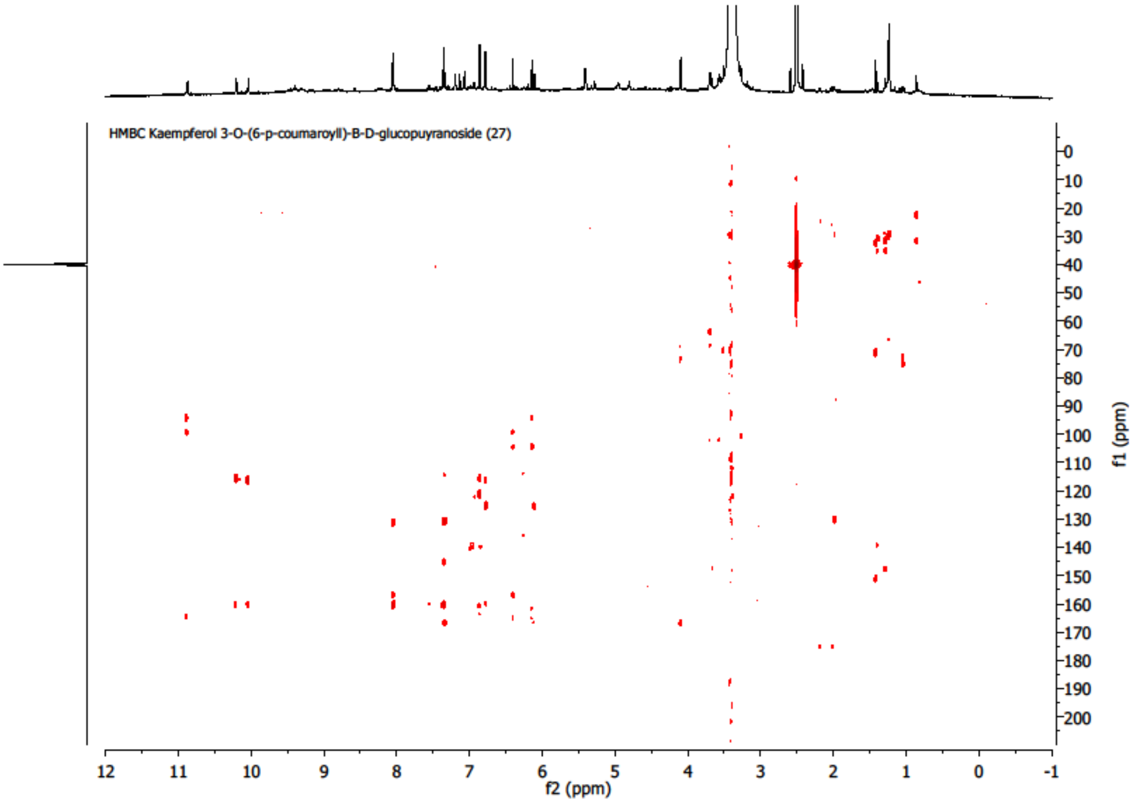

Figure S36. HMBC NMR spectrum (DMSO-*d6*) of Kaempferol 3-*O*-(6′′-*p*-coumaroyl)-β-D-glucopyranoside (**27**).

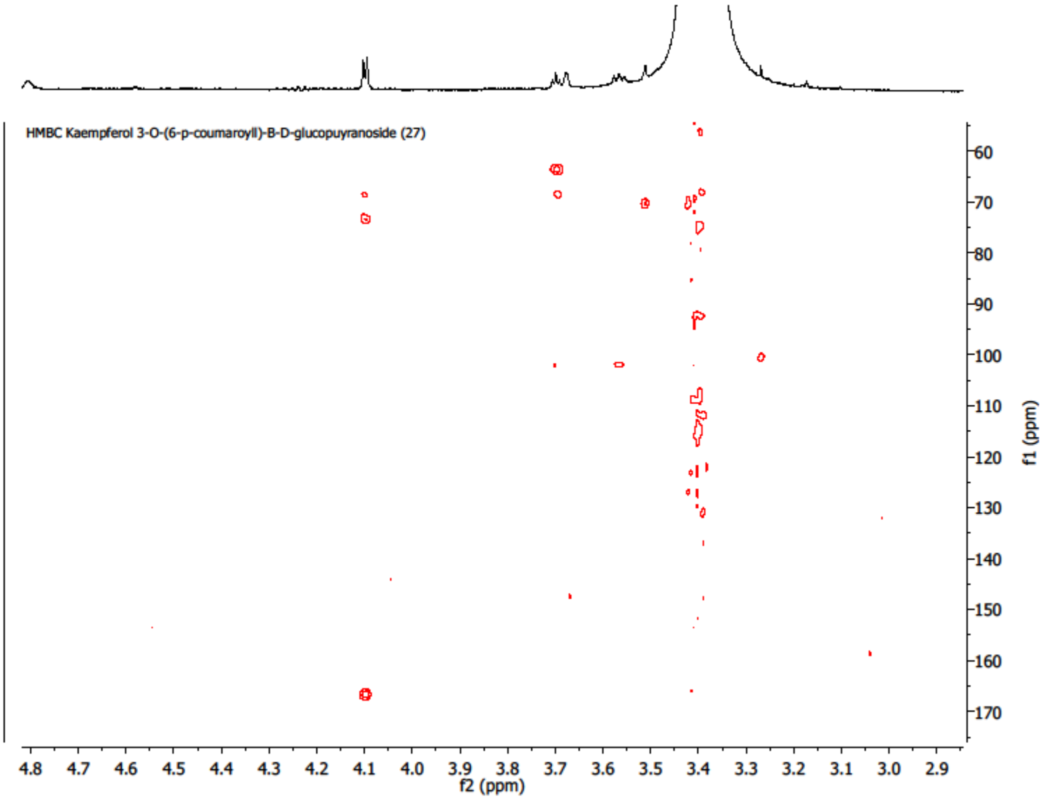

Figure S37. Zoom from 2.9 to 4.8 ppm, HMBC NMR spectrum (DMSO-*d6*) of Kaempferol 3-*O*-(6′′-*p*-coumaroyl)-β-D-glucopyranoside (**27**).

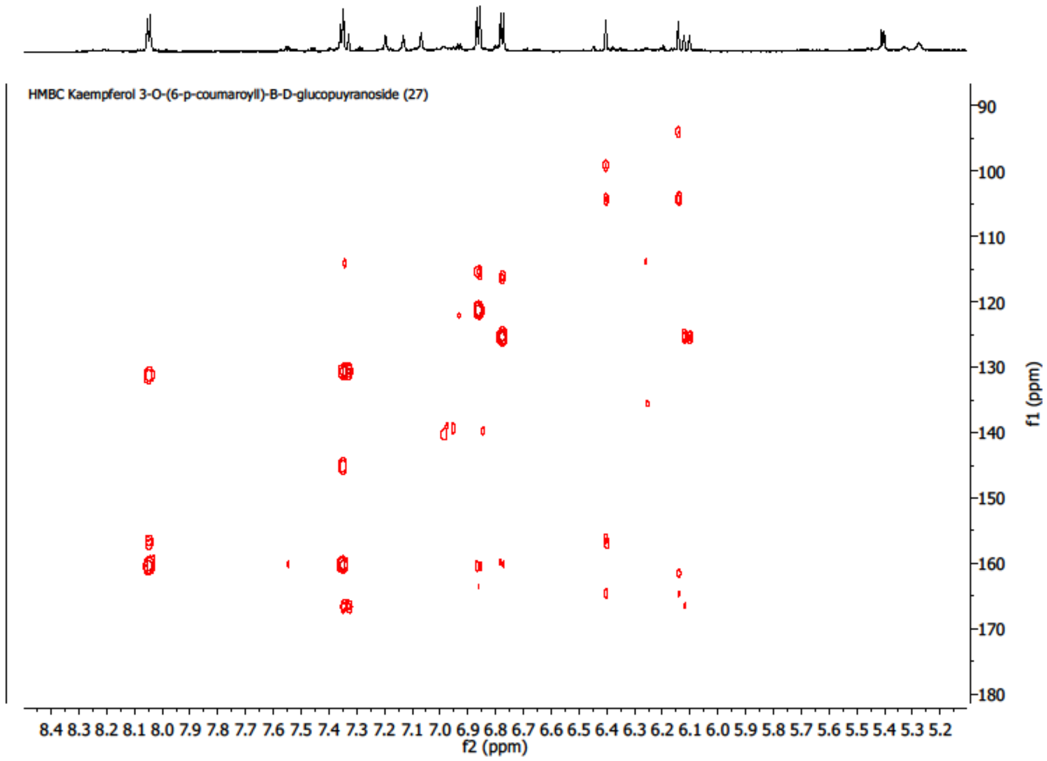

Figure S38. Zoom from 5.2 to 8.4 ppm, HMBC NMR spectrum (DMSO-*d*6) of Kaempferol 3-*O*-(6′′-*p*-coumaroyl)-β-D-glucopyranoside (**27**).

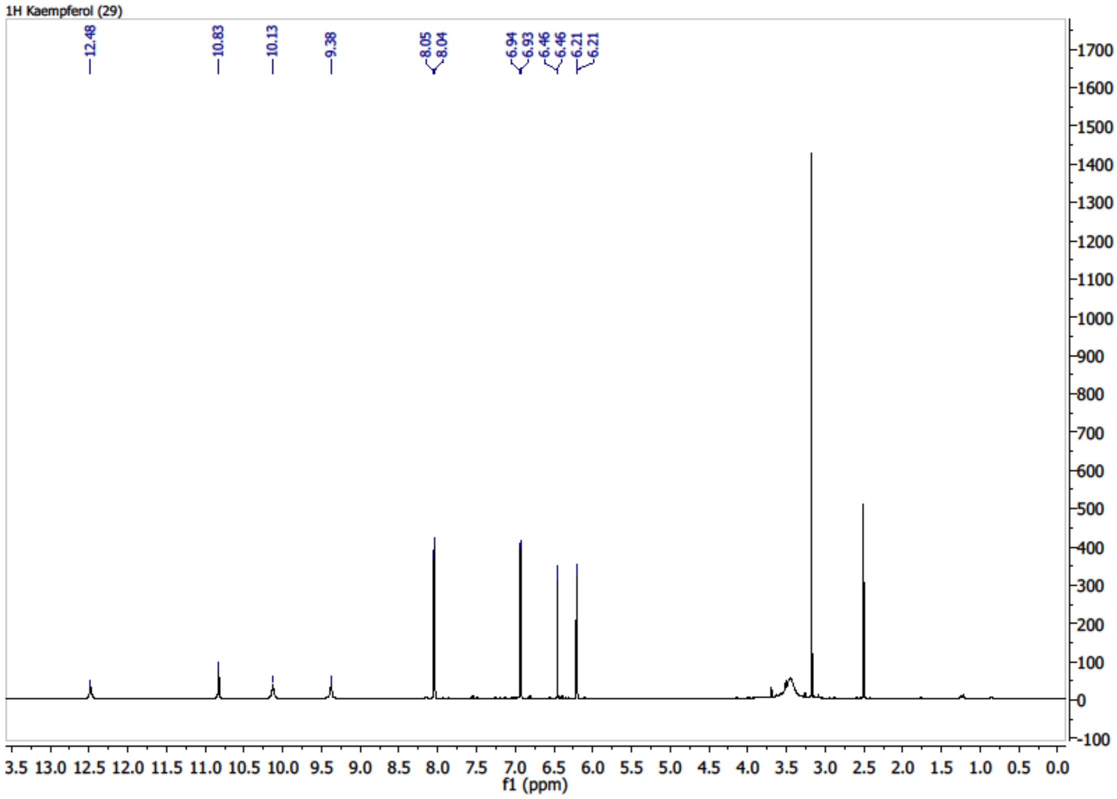

Figure S39. ^{1}H NMR spectrum (DMSO-*d*6) of kaempferol (**29**).

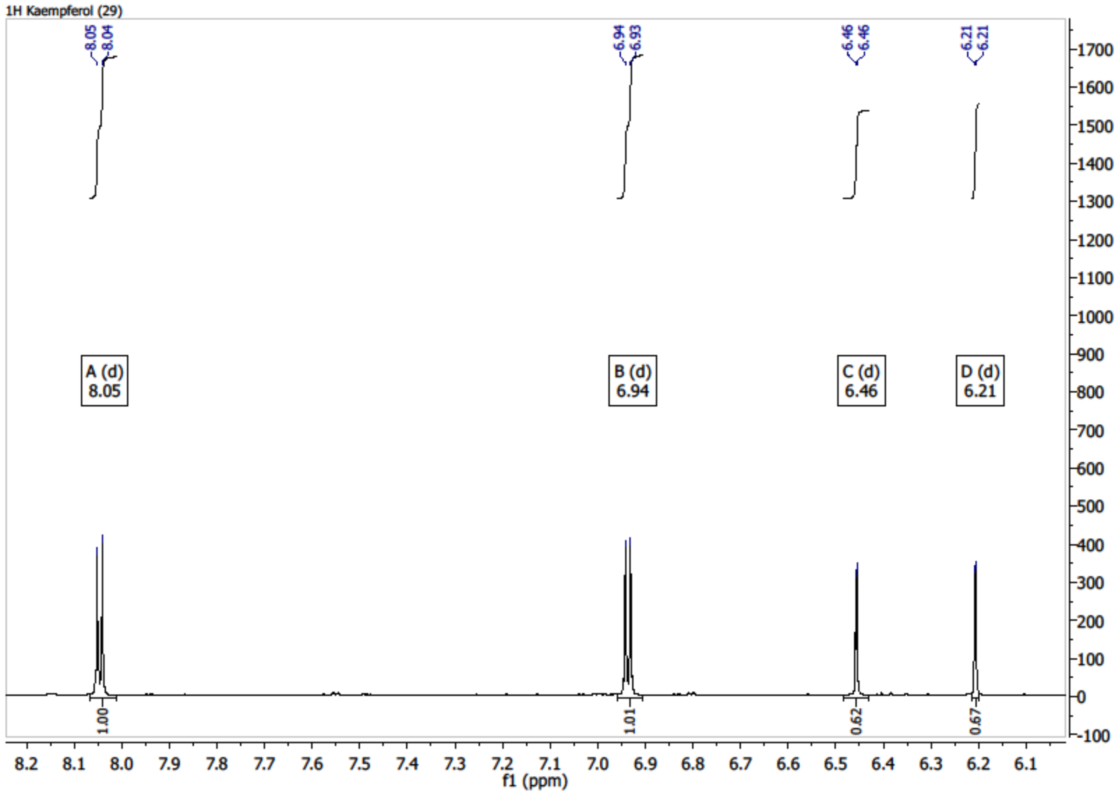

Figure S40. Zoom from 6.1 to 8.2 ppm, ^{1}H NMR spectrum (DMSO-*d6*) of kaempferol (**29**).

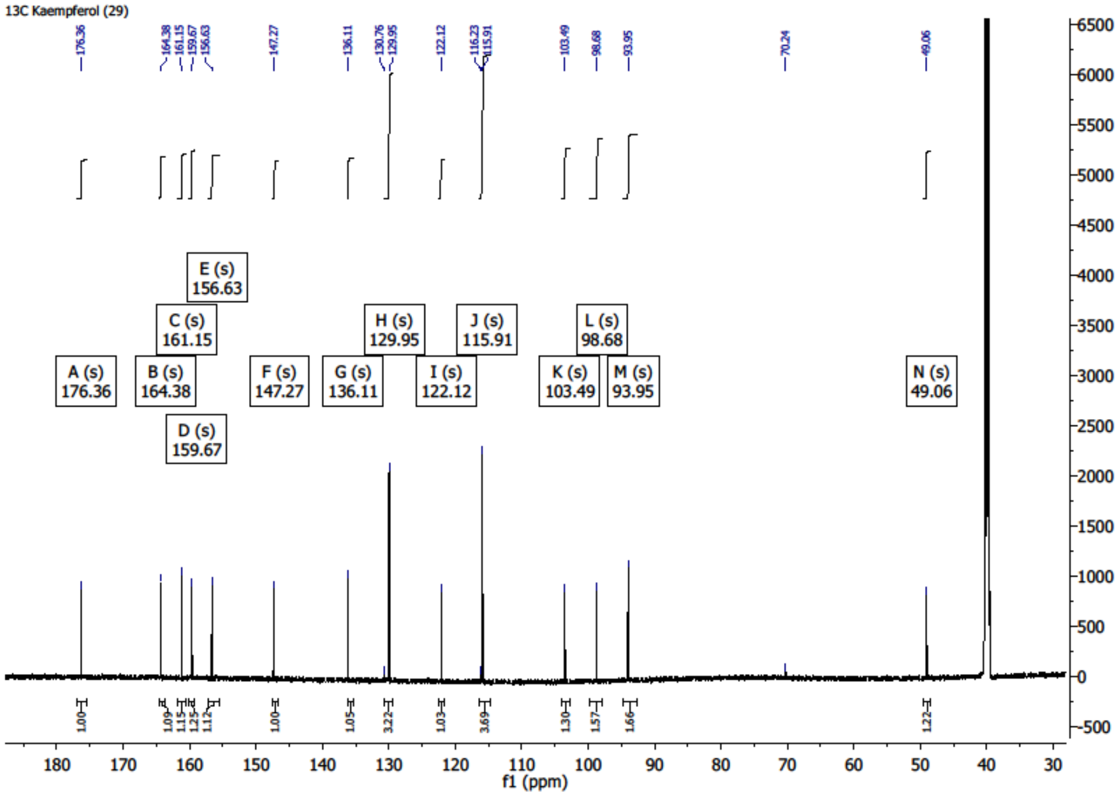

Figure S41. ^{13}C NMR spectrum (DMSO-*d6*) of kaempferol (**29**).

Table S1. NMR data of ^{1}H, ^{13}C and HMBC for kaempferol 3-*O*-(6′′-*O*-galloyl)-β-D-glucopyranoside and kaempferol 3-*O*-(2′′-*O*-galloyl)-β-D-glucopyranoside (**13**, **16** or **19**).

	Kaempferol 3-*O*-(6′′-*O*-galloyl)-β-D-glucopyranoside			Kaempferol 3-*O*-(2′′-*O*-galloyl)-β-D-glucopyranoside		
Carbon number	^{1}H	^{13}C*	HMBC	^{1}H	^{13}C*	HMBC
2	-	157.15	-	-	155.46	-
3	-	133.52	-	-	133.22	-
4	-	-	-	-	-	-
5	-	161.90	-	-	160.00	-
6	6.21 d (*J*= 2.0 Hz)	99.58	C8, C10, C7, C5	6.21 d (*J*= 2.0 Hz)	98.02	C8, C10, C7, C5
7	-	164.50	-	-	163.30	-
8	6.45 d (*J*= 2.0 Hz)	94.91	C10, C9, C7, C6	6.45 d (*J*= 2.0 Hz)	93.05	C10, C6, C7, C9
9	-	157.26	-	-	155.50	-
10	-	104.66	-	-	102.70	-
1'	-	121.04	-	-	120.90	-
2',6'	7.94 d (*J*= 8.8 Hz)	131.19	C2, C4', C6'or 2'	8.04 d (*J*= 8.8 Hz)	129.54	C2'or 6', C4', C2, C3'''or 5'''
3',5'	6.77 d (*J*= 8.8 Hz)	116.46	C4', C1', C5' or 3'	6.89/6.93 d (*J*= 8.8 Hz)	114.66	C3'''or 5''', C4', C1'
4'	-	150.58	-	-	158.40	-
1''	5.45 d (*J*= 7.6 Hz)	102.17	C3	5.47 d (*J*= 7.6 Hz)	100.38	C3
2''	3.24 - 3.50*	74.52	C1'', C3''	3.09 - 3.22*	73.68	C1, C3
3''		76.51	C2'', C4''		75.87	C2, C4
4''		69.89	C2'', C6''		69.48	C5
5''		74.60	-		76.74	C3, C4
6''	4.17 dd (*J*= 12 and 3.8 Hz), 4.26 dd (*J*= 12 and 2.0 Hz)	63.15	C5'', C4'', C7'''	3.62 and 3.38*	60.68	C5, C4
1'''	-	120.00	-	-	124.00	-
2''',6'''	6.92 s	109.70	C7''', C2'''or 6''', C3'''or 5''', C4''', C1'''	6.90 s	108.45	C7''', C4''', C3'''ou 5''', C1''', C2''' or 6'''
3''',5'''	-	146.11	-	-	145.96	-
4'''	-	138.94	-	-	138.86	-
7'''	-	165.36	-	-	162.11	-
OH-C5	12.52 s	-	-			
OH-C7	10.87 s	-	-			
OH-C4'	10.06 s	-	-			

*Chemical shifts of ^{13}C NMR were obtained by correlations in HSQC and HMBC.

Table S2. NMR data of ^{1}H, ^{13}C and HMBC for kaempferol 3-*O*-glucuronide-6''-*O*-methylester (**23**).

Carbon number	^{1}H	^{13}C*	HMBC
2	-	157.17	-
3	-	133.58	-
4	-	-	-
5	-	161.58	-
6	6.23 d (*J*= 2.0 Hz)	99.53	C8, C10, C7
7	-	164.78	-
8	6.45 d (*J*= 2.0 Hz)	94.10	C6, C10, C7, C9
9	-	156.94	-
10	-	104.46	-
1'	-	121.05	-
2',6'	8.02 d (*J*= 8.9 Hz)	131.73	C3, C2' or 6', C4'
3',5'	6.89 d (*J*= 8.9 Hz)	115.50	C1', C3' or 5', C4'
4'	-	160.62	-
1''	5.47 d (*J*= 7.7 Hz)	101.74	C3
2''		74.60	C1'', C3'',
3''	3.24-3.36*	75.92	C2'', C4''
4''		71.95	C5'', C6''
5''	3.73 d (*J*= 9.7 Hz)	76.13	C1'', C6'', C4''
6''	-	169.52	-
MeO-C6′′	3.57 s	52.32	C6''
OH-C5	12.51 s	-	-
OH-C7	10.97 s	-	-
OH-C4'	10.22 s	-	-

*Chemical shifts of ^{13}C NMR were obtained by correlations in HSQC and HMBC.

Table S3. NMR data of ^{1}H, ^{13}C and HMBC for quercetin 3-*O*-(6′′-*O*-*p*-coumaroyl)-β-D-glucopyranoside (**25**) and kaempferol 3-*O*-(6′′-*O*-*p*-coumaroyl)-β-D-glucopyranoside (**27**).

	Quercetin 3-*O*-(6′′-*O*-*p*-coumaroyl)-β-D-glucopyranoside		Kaempferol 3-*O*-(6′′-*O*-*p*-coumaroyl)-β-D-glucopyranoside		
Carbon number	^{1}H	^{13}C*	^{1}H	^{13}C*	HMBC
2	-	-	-	156.96	-
3	-	-	-	-	-
4	-	-	-	-	-
5	-	-	-	161.69	-
6	6.14 d (*J*= 2.1 Hz)	99.19	6.14 d (*J*= 2.1 Hz)	99.30	C8, C10, C5
7	-	-	-	164.56	-
8	6.38 d (*J*= 2.1 Hz)	93.90	6.40 d (*J*= 2.1 Hz)	94.19	C10, C9, C7, C6
9	-	-	-	156.55	-
10	-	-	-	104.20	-
1'	-	-	-	125.58	-
2'	7.52 d (*J*= 2.3 Hz)	116.42	8.05 d (*J*= 8.4 Hz)	131.47	C2, C4', C6'or 2'
3'	-	-	6.78 d (*J*= 8.4 Hz)	116.35	C1', C5' or 3'
4'	-	-	-	160.38	-
5'	6.78 d (*J*= 8.4 Hz)	116.01	6.78 d (*J*= 8.4 Hz)	116.35	
6'	7.66 dd (*J*= 8.4 Hz and 2.3 Hz)	122.38	8.05 d (*J*= 8.4 Hz)	131.47	
1''	5.42 d (*J*= 7.8 Hz)	102.03	5.41 d (*J*= 7.7 Hz)	102.04	-
2''	-	-	3.51 - 3.70	71.47	C1
3''	-	-		70.32	-
4''	-	-		73.40	C5''ou C3'', C1'', C6''or C2''
5''	-	-		69.05	-
6''	4.12 dd (*J*= 11.5 Hz and 4.6Hz)	63.62	4.10 d (*J*= 6.2 Hz)	63.71	C9''', C4'', C5''or C3''
1'''	-	-	-	121.33 or 125.05	-
2''',6'''	7.37 d (*J*= 8.4 Hz)	130.62	7.35 d (*J*= 8.7 Hz)	130.66	C7''', C4'''
3''',5'''	6.83 d (*J*= 8.4 Hz)	115.61	6.86 d (*J*= 8.7 Hz)	115.52	C1''', C5''or C3'', C4'''
4'''	-	-	-	160.53	-
7'''	7.36 d (*J*= 15.7 Hz)	145.31	7.34 d (*J*= 15.9 Hz)	145.24	C9''', C2'''or C6'''
8'''	6.13 d (*J*= 15.7 Hz)	114.10	6.11 d (*J*= 15.9 Hz)	114.29	-
9'''	-	-	-	166.85	-
OH-C5	12.64 s	-	12.59 s	-	-
OH-C7	10.84 s	-	10.87 s	-	C7, C6, C8
OH-C3'	9.16 s	-		-	-
OH-C4'	9.73 s	-	10.03 s	-	-
OH-C4'''	10.01 s	-	10.20 s	-	-

*Chemical shifts of ^{13}C NMR were obtained by correlations in HSQC.

Table S4. NMR data of ^1H and ^{13}C for Kaempferol (**29**).

Carbon number	^1H	^{13}C
2	-	147.27
3	-	136.11
4	-	176.36
5	-	161.15
6	6.21 d (*J*= 2.0 Hz)	96.68
7	-	164.38
8	6.46 d (*J*= 2.0 Hz)	93.95
9	-	156.63
10	-	103.49
1'	-	122.12
2',6'	8.05 d (*J*= 8.9 Hz)	129.95
4'		159.67
3',5'	6.94 d (*J*= 8.9 Hz)	115.91
OH-C3	9.38 s	
OH-C5	12.48 s	-
OH-C7	10.83 s	-
OH-C4'	10.13 s	-
