# Supplementary figures and images for: Acylated Flavonoid Glycosides are the Main Pigments that Determine the Flower Colour of the Brazilian Native Tree Tibouchina pulchra (Cham.) Cogn
Source: Molecules. 2019 Feb 16;24(4):718. doi: 10.3390/molecules24040718 (PMC6412660; doi:10.3390/molecules24040718)

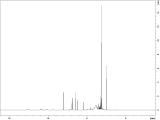

Supplement: Supplementary file 1 [file molecules-24-00718-s001.zip › Compound 17/10/pdata/1/thumb.png]

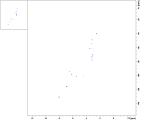

Supplement: Supplementary file 1 [file molecules-24-00718-s001.zip › Compound 17/11/pdata/1/thumb.png]

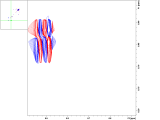

Supplement: Supplementary file 1 [file molecules-24-00718-s001.zip › Compound 17/12/pdata/1/thumb.png]

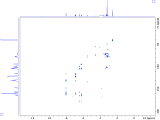

Supplement: Supplementary file 1 [file molecules-24-00718-s001.zip › Compound 17/13/pdata/1/thumb.png]

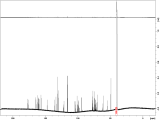

Supplement: Supplementary file 1 [file molecules-24-00718-s001.zip › Compound 17/14/pdata/1/thumb.png]
