# Supplementary material for: Acylated Flavonoid Glycosides are the Main Pigments that Determine the Flower Colour of the Brazilian Native Tree Tibouchina pulchra (Cham.) Cogn
Source: Molecules. 2019 Feb 16;24(4):718. doi: 10.3390/molecules24040718 (PMC6412660; doi:10.3390/molecules24040718)
Supplement: Supplementary file 1 [file molecules-24-00718-s001.zip › Article_Supplementary figures feb 15.doc]

Article

Acylated flavonoid glycosides are the main pigments that determine the flower colour of the Brazilian native tree *Tibouchina pulchra* (Cham.) Cogn.

Fernanda Mendes Rezende ^1,^*, Marcelo José Pena Ferreira ^1^, Mads Hartvig Clausen ^2^,
Magdalena Rossi ^1^ and Claudia Maria Furlan ^1,^*

^1^	Botany Department, Institute of Bioscience, University of São Paulo (USP), São Paulo, Brazil; marcelopena@ib.usp.br (M.J.P.F.); magdarossirosso@gmail.com (M.R.)

^2^	Center for Nanomedicine and Theranostics, Department of Chemistry, Technical University of Denmark, Kgs. Lyngby, Denmark; mhc@kemi.dtu.dk

*****	Correspondence: rezendefm@usp.br (F.M.R.); furlancm@ib.usp.br (C.M.F.); Tel.: +55-011-3091-8065

SUPPLEMENTARY MATERIAL

**Figure S1.** MS^+^ spectra of compounds **1** to **30**. Structures represent the proposed compound and its main fragmentation……………………………………………………………………………………………………………….1

**Figure S2.** ^1^H NMR spectrum (DMSO-*d*6) of kaempferol 3-*O*-(2’’-galloyl)-β-d-glucopyranoside (**13**, **16** or **19**)…………………………………………………………………………………………………………………………….8

**Figure S3.** Zoom from 5.2 to 8.5 ppm, ^1^H NMR spectrum (DMSO-*d*6) of kaempferol 3-*O*-(2’’-galloyl)-β-d-glucopyranoside (**13**, **16** or **19**) ……………………………………………………………………8

**Figure S4.** HSQC NMR spectrum (DMSO-*d*6) of kaempferol 3-*O*-(2’’-galloyl)-β-d-glucopyranoside (**13**, **16** or **19**)…………………………………………………………………………………………………………………………….9

**Figure S5.** Zoom from 2.7 to 3.8 ppm, HSQC NMR spectrum (DMSO-*d*6) of kaempferol 3-*O*-(2’’-galloyl)-β-d-glucopyranoside (**13**, **16** or **19**)……………………………………………………………………9

**Figure S6.** HMBC NMR spectrum (DMSO-*d*6) of kaempferol 3-*O*-(2’’-galloyl)-β-d-glucopyranoside (**13**, **16** or **19**)…………………………………………………………………………………………………………………………...10

**Figure S7.** Zoom from 2.7 to 3.9 ppm, HMBC NMR spectrum (DMSO-*d*6) of kaempferol 3-*O*-(2’’-galloyl)-β-d-glucopyranoside (**13**, **16** or **19**) …………………………………………………………………..10

**Figure S8.** Zoom from 5.2 to 8.6 ppm, HMBC NMR spectrum (DMSO-*d*6) of kaempferol 3-*O*-(2’’-galloyl)-β-d-glucopyranoside (**13**, **16** or **19**) .………………………………………………………………….11

**Figure S9.** ^1^H NMR spectrum (DMSO-*d*6) of kaempferol 3-*O*-(6’’-galloyl)-β-d-glucopyranoside (**13**, **16** or **19**)…………………………………………………………………………………………………………………………...11

**Figure S10.** Zoom from 5.1 to 8.1 ppm, ^1^H NMR spectrum (DMSO-*d*6) of kaempferol 3-*O*-(6’’-galloyl)-β-d-glucopyranoside (**13**, **16** or **19**). ………………………………………………………………….12

**Figure S11.** HSQC NMR spectrum (DMSO-*d*6) of kaempferol 3-*O*-(6’’-galloyl)-β-d-glucopyranoside (**13**, **16** or **19**)…………………………………………………………………………………………………………………………...12

**Figure S12.** Zoom from 3.0 to 4.6 ppm, HSQC NMR spectrum (DMSO-*d*6) of kaempferol 3-*O*-(6’’-galloyl)-β-d-glucopyranoside (**13**, **16** or **19**)………………………………………………………………….. 13

**Figure S13.** HMBC NMR spectrum (DMSO-d6) of kaempferol 3-O-(6’’-galloyl)-β-D-glucopyranoside (13, 16 or 19)…………………………………………………………………………………………………………………………...13

**Figure S14.** Zoom from 2.8 to 4.9 ppm, HMBC NMR spectrum (DMSO-*d*6) of kaempferol 3-*O*-(6’’-galloyl)-β-d-glucopyranoside (**13**, **16** or **19**)…………………………………………………………………. .14

**Figure S15.** Zoom from 5.2 to 8.4 ppm, HMBC NMR spectrum (DMSO-*d*6) of kaempferol 3-*O*-(6’’-galloyl)-β-d-glucopyranoside (**13**, **16** or **19**)………………………………………………………………….. 14

**Figure S16.** ^1^H NMR spectrum (DMSO-*d*6) of mixture (**17**)………………………………………………………………………………………………………………………….15

**Figure S17.** Zoom from 5.2 to 8.2 ppm, ^1^H NMR spectrum (DMSO-*d*6) of mixture (**17**)…………………………….............................................................................................................................................15

**Figure S18.** ^13^C NMR spectrum (DMSO-*d*6) of mixture (**17**)………………………………………………………………………………………………………………………….16

**Figure S19.** HMBC NMR spectrum (DMSO-*d*6) of mixture (**17**)……………………………………………………….....................................................................................................16

**Figure S20.** Zoom from 2.4 to 4.5 ppm, HMBC NMR spectrum (DMSO-*d*6) of mixture (**17**)………………………………………………………………………………………………………………………….17

**Figure S21.** Zoom from 4.8 to 8.6 ppm, HMBC NMR spectrum (DMSO-*d*6) of mixture (**17**)……………………………………………………………………………………………………………………….....17

**Figure S22.** ^1^H NMR spectrum (DMSO-*d*6) of kaempferol 3-*O*-glucoronide-6’’-*O*-methylester (**23**)……………….................................................................................................................................................................18

**Figure S23.** Zoom from 5.1 to 8.1 ppm, ^1^H NMR spectrum (DMSO-*d*6) of kaempferol 3-*O*-glucoronide-6’’-*O*-methylester (**23**)………………………………………………………………………………...18

**Figure S24.** HSQC NMR spectrum (DMSO-*d*6) of kaempferol 3-*O*-glucoronide-6’’-*O*-methylester (**23**)……………………………………………………………………………………………………………………….....19

**Figure S25.** Zoom from 2.9 to 4.5 ppm, HSQC NMR spectrum (DMSO-*d*6) of kaempferol 3-*O*-glucoronide-6’’-*O*-methylester (**23**)………………………………………………………………………………...19

**Figure S26.** Zoom from 5.0 to 8.6 ppm, HMBC NMR spectrum (DMSO-*d*6) of kaempferol 3-*O*-glucoronide-6’’-*O*-methylester (**23**)………………………………………………………………………………...20

**Figure S27.** ^1^H NMR spectrum (DMSO-*d*6) of quercetin 3-*O*-(6’’-*p*-coumaroyl)-β-d-glucopyranoside (**25**)………………………………………………………………………………………………………………………….20

**Figure S28.** Zoom from 5.1 to 8.1 ppm, ^1^H NMR spectrum (DMSO-*d*6) of quercetin 3-*O*-(6’’-*p*-coumaroyl)-β-d-glucopyranoside (**25**)………………………………………………………………………21

**Figure S29.** HSQC NMR spectrum (DMSO-*d*6) of quercetin 3-*O*-(6’’-*p*-coumaroyl)-β-d-glucopyranoside (**25**)………………………………………………………………………………………………………………………….21

**Figure S30.** Zoom from 2.7 to 4.7 ppm, HSQC NMR spectrum (DMSO-*d*6) of quercetin 3-*O*-(6’’-*p*-coumaroyl)-β-d-glucopyranoside (**25**)………………………………………………………………………22

**Figure S31.** Zoom from 5.0 to 8.8 ppm, HSQC NMR spectrum (DMSO-*d*6) of quercetin 3-*O*-(6’’-*p*-coumaroyl)-β-d-glucopyranoside (**25**)………………………………………………………………………22

**Figure S32.** ^1^H NMR spectrum (DMSO-*d*6) of kaempferol 3-*O*-(6’’-*p*-coumaroyl)-β-d-glucopyranoside (**27**)……………………………………………………………………………………………………………………….....23

**Figure S33.** Zoom from 5.1 to 8.1 ppm, ^1^H NMR spectrum (DMSO-*d*6) of kaempferol 3-*O*-(6’’-*p*-coumaroyl)-β-d-glucopyranoside (**27**)…………………………………………………………………..…..23

**Figure S34.** HSQC NMR spectrum (DMSO-*d*6) of kaempferol 3-*O*-(6’’-*p*-coumaroyl)-β-d-glucopyranoside (**27**)………………………………………………………………………………………………………………………….24

**Figure S35.** Zoom from 3.0 to 4.6 ppm, HSQC NMR spectrum (DMSO-*d*6) of kaempferol 3-*O*-(6’’-*p*-coumaroyl)-β-d-glucopyranoside (**27**)………………………………………………………………………24

**Figure S36.** HMBC NMR spectrum (DMSO-*d*6) of kaempferol 3-*O*-(6’’-*p*-coumaroyl)-β-d-glucopyranoside (**27**)………………………………………………………………………………………………………………………….25

**Figure S37.** Zoom from 2.9 to 4.8 ppm, HMBC NMR spectrum (DMSO-*d*6) of kaempferol 3-*O*-(6’’-*p*-coumaroyl)-β-d-glucopyranoside (**27**)……………………………………………………………………....25

**Figure S38.** Zoom from 5.2 to 8.4 ppm, HMBC NMR spectrum (DMSO-*d*6) of kaempferol 3-*O*-(6’’-*p*-coumaroyl)-β-d-glucopyranoside (**27**)………………………………………………………………………26

**Figure S39.** ^1^H NMR spectrum (DMSO-*d*6) of kaempferol (2**9**)………………………………………………………………………………………………………….………………26

**Figure S40.** Zoom from 6.1 to 8.2 ppm, ^1^H NMR spectrum (DMSO-*d*6) of kaempferol (2**9**)………………………………………………………………………………………………………………………….27

**Figure S41.** ^13^C NMR spectrum (DMSO-*d*6) of kaempferol (**29**)……………………………………………………………………………………………………………………….....27

**Table S1**. NMR data of ^1^H, ^13^C and HMBC for kaempferol 3-*O*-(6’’-*O*-galloyl)-β-D-glucopyranoside and Kaempferol 3-*O*-(2’’-*O*-galloyl)-β-D-glucopyranoside (13, 16 or 19)…………………………………………………28

**Table S2.** NMR data of ^1^H, ^13^C and HMBC for kaempferol 3-*O*-glucuronide-6''-*O*-methylester (**23**)…………….29

**Table S3.** NMR data of ^1^H, ^13^C and HMBC for quercetin 3-*O*-(6’’-*O*-*p*-coumaroyl)-β-D-glucopyranoside (**25**) and Kaempferol 3-*O*-(6’’-*O*-*p*-coumaroyl)-β-D-glucopyranoside (**27**)…………………………………………………….30

**Table S4.** NMR data of ^1^H and ^13^C for kaempferol (29)………….……………………………………………………31

**Figure S1.** MS^+^ spectra of compounds **1** to **30**. Structures represent the proposed compound and its main fragmentation.

**Figure S1 (continue).**

**Figure S1 (continue).**

**Figure S1 (continue).**

**Figure S1 (continue).**

**Figure S1 (continue).**

**Figure S1 (continue).**

**Figure S2.** ^1^H NMR spectrum (DMSO-*d*6) of kaempferol 3-*O*-(2’’-galloyl)-β-d-glucopyranoside (**13**, **16** or **19**).

**Figure S3.** Zoom from 5.2 to 8.5 ppm, ^1^H NMR spectrum (DMSO-*d*6) of kaempferol 3-*O*-(2’’-galloyl)-β-d-glucopyranoside (**13**, **16** or **19**).

**Figure S4.** HSQC NMR spectrum (DMSO-*d*6) of kaempferol 3-*O*-(2’’-galloyl)-β-d-glucopyranoside (**13**, **16** or **19**).

**Figure S5.** Zoom from 2.7 to 3.8 ppm, HSQC NMR spectrum (DMSO-*d*6) of kaempferol 3-*O*-(2’’-galloyl)-β-d-glucopyranoside (**13**, **16** or **19**).

**Figure S6.** HMBC NMR spectrum (DMSO-*d*6) of kaempferol 3-*O*-(2’’-galloyl)-β-d-glucopyranoside (**13**, **16** or **19**).

**Figure S7.** Zoom from 2.7 to 3.9 ppm, HMBC NMR spectrum (DMSO-*d*6) of kaempferol 3-*O*-(2’’-galloyl)-β-d-glucopyranoside (**13**, **16** or **19**).

**Figure S8.** Zoom from 5.2 to 8.6 ppm, HMBC NMR spectrum (DMSO-*d*6) of kaempferol 3-*O*-(2’’-galloyl)-β-d-glucopyranoside (**13**, **16** or **19**).

**Figure S9.** ^1^H NMR spectrum (DMSO-*d*6) of kaempferol 3-*O*-(6’’-galloyl)-β-d-glucopyranoside (**13**, **16** or **19**).

**Figure S10.** Zoom from 5.1 to 8.1 ppm, ^1^H NMR spectrum (DMSO-*d*6) of kaempferol 3-*O*-(6’’-galloyl)-β-d-glucopyranoside (**13**, **16** or **19**).

**Figure S11.** HSQC NMR spectrum (DMSO-*d*6) of kaempferol 3-*O*-(6’’-galloyl)-β-d-glucopyranoside (**13**, **16** or **19**).

**Figure S12.** Zoom from 3.0 to 4.6 ppm, HSQC NMR spectrum (DMSO-*d*6) of kaempferol 3-*O*-(6’’-galloyl)-β-d-glucopyranoside (**13**, **16** or **19**).

**Figure S13.** HMBC NMR spectrum (DMSO-d6) of kaempferol 3-O-(6’’-galloyl)-β-D-glucopyranoside (**13**, **16** or **19**).

**Figure S14.** Zoom from 2.8 to 4.9 ppm, HMBC NMR spectrum (DMSO-*d*6) of kaempferol 3-*O*-(6’’-galloyl)-β-d-glucopyranoside (**13**, **16** or **19**).

**Figure S15.** Zoom from 5.2 to 8.4 ppm, HMBC NMR spectrum (DMSO-*d*6) of kaempferol 3-*O*-(6’’-galloyl)-β-d-glucopyranoside (**13**, **16** or **19**).

**Figure S16.** ^1^H NMR spectrum (DMSO-*d*6) of mixture (**17**).

**Figure S17.** Zoom from 5.2 to 8.2 ppm, ^1^H NMR spectrum (DMSO-*d*6) of mixture (**17**).

**Figure S18.** ^13^C NMR spectrum (DMSO-*d*6) of mixture (**17**).

**Figure S19.** HMBC NMR spectrum (DMSO-*d*6) of mixture (**17**).

**Figure S20.** Zoom from 2.4 to 4.5 ppm, HMBC NMR spectrum (DMSO-*d*6) of mixture (**17**).

**Figure S21.** Zoom from 4.8 to 8.6 ppm, HMBC NMR spectrum (DMSO-*d*6) of mixture (**17**).

**Figure S22.** ^1^H NMR spectrum (DMSO-*d*6) of kaempferol 3-*O*-glucoronide-6’’-*O*-methylester (**23**).

**Figure S23.** Zoom from 5.1 to 8.1 ppm, ^1^H NMR spectrum (DMSO-*d*6) of kaempferol 3-*O*-glucoronide-6’’-*O*-methylester (**23**).

**Figure S24.** HSQC NMR spectrum (DMSO-*d*6) of kaempferol 3-*O*-glucoronide-6’’-*O*-methylester (**23**).

**Figure S25.** Zoom from 2.9 to 4.5 ppm, HSQC NMR spectrum (DMSO-*d*6) of kaempferol 3-*O*-glucoronide-6’’-*O*-methylester (**23**).

**Figure S26.** Zoom from 5.0 to 8.6 ppm, HMBC NMR spectrum (DMSO-*d*6) of kaempferol 3-*O*-glucoronide-6’’-*O*-methylester (**23**).

**Figure S27.** ^1^H NMR spectrum (DMSO-*d*6) of Quercetin 3-*O*-(6’’-*p*-coumaroyl)-β-d-glucopyranoside (**25**).

**Figure S28.** Zoom from 5.1 to 8.1 ppm, ^1^H NMR spectrum (DMSO-*d*6) of Quercetin 3-*O*-(6’’-*p*-coumaroyl)-β-d-glucopyranoside (**25**).

**Figure S29.** HSQC NMR spectrum (DMSO-*d*6) of Quercetin 3-*O*-(6’’-*p*-coumaroyl)-β-d-glucopyranoside (**25**).

**Figure S30.** Zoom from 2.7 to 4.7 ppm, HSQC NMR spectrum (DMSO-*d*6) of Quercetin 3-*O*-(6’’-*p*-coumaroyl)-β-d-glucopyranoside (**25**).

**Figure S31.** Zoom from 5.0 to 8.8 ppm, HSQC NMR spectrum (DMSO-*d*6) of Quercetin 3-*O*-(6’’-*p*-coumaroyl)-β-d-glucopyranoside (**25**).

**Figure S32.** ^1^H NMR spectrum (DMSO-*d*6) of Kaempferol 3-*O*-(6’’-*p*-coumaroyl)-β-d-glucopyranoside (**27**).

**Figure S33.** Zoom from 5.1 to 8.1 ppm, ^1^H NMR spectrum (DMSO-*d*6) of Kaempferol 3-*O*-(6’’-*p*-coumaroyl)-β-d-glucopyranoside (**27**).

**Figure S34.** HSQC NMR spectrum (DMSO-*d*6) of Kaempferol 3-*O*-(6’’-*p*-coumaroyl)-β-d-glucopyranoside (**27**).

**Figure S35.** Zoom from 3.0 to 4.6 ppm, HSQC NMR spectrum (DMSO-*d*6) of Kaempferol 3-*O*-(6’’-*p*-coumaroyl)-β-d-glucopyranoside (**27**).

**Figure S36.** HMBC NMR spectrum (DMSO-*d*6) of Kaempferol 3-*O*-(6’’-*p*-coumaroyl)-β-d-glucopyranoside (**27**).

**Figure S37.** Zoom from 2.9 to 4.8 ppm, HMBC NMR spectrum (DMSO-*d*6) of Kaempferol 3-*O*-(6’’-*p*-coumaroyl)-β-d-glucopyranoside (**27**).

**Figure S38.** Zoom from 5.2 to 8.4 ppm, HMBC NMR spectrum (DMSO-*d*6) of Kaempferol 3-*O*-(6’’-*p*-coumaroyl)-β-d-glucopyranoside (**27**).

**Figure S39.** ^1^H NMR spectrum (DMSO-*d*6) of kaempferol (2**9**).

**Figure S40.** Zoom from 6.1 to 8.2 ppm, ^1^H NMR spectrum (DMSO-*d*6) of kaempferol (2**9**).

**Figure S41.** ^13^C NMR spectrum (DMSO-*d*6) of kaempferol (**29**).

Table S1. NMR data of ^1^H, ^13^C and HMBC for kaempferol 3-*O*-(6’’-*O*-galloyl)-β-D-glucopyranoside and kaempferol 3-*O*-(2’’-*O*-galloyl)-β-D-glucopyranoside (13, 16 or 19).

|  | Kaempferol 3-*O*-(6’’-*O*-galloyl)-β-D-glucopyranoside | | | Kaempferol 3-*O*-(2’’-*O*-galloyl)-β-D-glucopyranoside | | | | | |  |
| --- | --- | --- | --- | --- | --- | --- | --- | --- | --- | --- |
| Carbon number | ^1^H | ^13^C* | HMBC | ^1^H | | ^13^C* | | HMBC | |  |
| 2 | - | 157.15 | - | - | 155.46 | | - | |  | |
| 3 | - | 133.52 | - | - | 133.22 | | - | | | |
| 4 | - | - | - | - | - | | - | | | |
| 5 | - | 161.90 | - | - | 160.00 | | - | | | |
| 6 | 6.21 d (*J*= 2.0 Hz) | 99.58 | C8, C10, C7, C5 | 6.21 d (*J*= 2.0 Hz) | 98.02 | | C8, C10, C7, C5 | | | |
| 7 | - | 164.50 | - | - | 163.30 | | - | | | |
| 8 | 6.45 d (*J*= 2.0 Hz) | 94.91 | C10, C9, C7, C6 | 6.45 d (*J*= 2.0 Hz) | 93.05 | | C10, C6, C7, C9 | | | |
| 9 | - | 157.26 | - | - | 155.50 | | - | | | |
| 10 | - | 104.66 | - | - | 102.70 | | - | | | |
| 1' | - | 121.04 | - | - | 120.90 | | - | | | |
| 2',6' | 7.94 d (*J*= 8.8 Hz) | 131.19 | C2, C4', C6'or 2' | 8.04 d (*J*= 8.8 Hz) | 129.54 | | C2'or 6', C4', C2, C3'''or 5''' | | | |
| 3',5' | 6.77 d (*J*= 8.8 Hz) | 116.46 | C4', C1', C5' or 3' | 6.89/6.93 d (*J*= 8.8 Hz) | 114.66 | | C3'''or 5''', C4', C1' | | | |
| 4' | - | 150.58 | - | - | 158.40 | | - | | | |
| 1'' | 5.45 d (*J*= 7.6 Hz) | 102.17 | C3 | 5.47 d (*J*= 7.6 Hz) | 100.38 | | C3 | | | |
| 2'' | 3.24 - 3.50* | 74.52 | C1'', C3'' | 3.09 - 3.22* | 73.68 | | C1, C3 | | | |
| 3'' |  | 76.51 | C2'', C4'' |  | 75.87 | | C2, C4 | | | |
| 4'' |  | 69.89 | C2'', C6'' |  | 69.48 | | C5 | | | |
| 5'' |  | 74.60 | - |  | 76.74 | | C3, C4 | | | |
| 6'' | 4.17 dd (*J*= 12 and 3.8 Hz), 4.26 dd (*J*= 12 and 2.0 Hz) | 63.15 | C5'', C4'', C7''' | 3.62 and 3.38* | 60.68 | | C5, C4 | | | |
| 1''' | - | 120.00 | - | - | 124.00 | | - | | | |
| 2''',6''' | 6.92 s | 109.70 | C7''', C2'''or 6''', C3'''or 5''', C4''', C1''' | 6.90 s | 108.45 | | C7''', C4''', C3'''ou 5''', C1''', C2''' or 6''' | | | |
| 3''',5''' | - | 146.11 | - | - | 145.96 | | - | | | |
| 4''' | - | 138.94 | - | - | 138.86 | | - | | | |
| 7''' | - | 165.36 | - | - | 162.11 | | - | | | |
| OH-C5 | 12.52 s | - | - |  |  | |  | | | |
| OH-C7 | 10.87 s | - | - |  |  | |  | | | |
| OH-C4' | 10.06 s | - | - |  |  | |  | | | |

*Chemical shifts of ^13^C NMR were obtained by correlations in HSQC and HMBC.

**Table S2.** NMR data of ^1^H, ^13^C and HMBC for kaempferol 3-*O*-glucuronide-6''-*O*-methylester (**23**).

| Carbon number | ^1^H | ^13^C* | HMBC |
| --- | --- | --- | --- |
| 2 | - | 157.17 | - |
| 3 | - | 133.58 | - |
| 4 | - | - | - |
| 5 | - | 161.58 | - |
| 6 | 6.23 d (*J*= 2.0 Hz) | 99.53 | C8, C10, C7 |
| 7 | - | 164.78 | - |
| 8 | 6.45 d (*J*= 2.0 Hz) | 94.10 | C6, C10, C7, C9 |
| 9 | - | 156.94 | - |
| 10 | - | 104.46 | - |
| 1' | - | 121.05 | - |
| 2',6' | 8.02 d (*J*= 8.9 Hz) | 131.73 | C3, C2' or 6', C4' |
| 3',5' | 6.89 d (*J*= 8.9 Hz) | 115.50 | C1', C3' or 5', C4' |
| 4' | - | 160.62 | - |
| 1'' | 5.47 d (*J*= 7.7 Hz) | 101.74 | C3 |
| 2'' | 3.24-3.36* | 74.60 | C1'', C3'', |
| 3'' |  | 75.92 | C2'', C4'' |
| 4'' |  | 71.95 | C5'', C6'' |
| 5'' | 3.73 d (*J*= 9.7 Hz) | 76.13 | C1'', C6'', C4'' |
| 6'' | - | 169.52 | - |
| MeO-C6’’ | 3.57 s | 52.32 | C6'' |
| OH-C5 | 12.51 s | - | - |
| OH-C7 | 10.97 s | - | - |
| OH-C4' | 10.22 s | - | - |

*Chemical shifts of ^13^C NMR were obtained by correlations in HSQC and HMBC.

**Table S3.** NMR data of ^1^H, ^13^C and HMBC for quercetin 3-*O*-(6’’-*O*-*p*-coumaroyl)-β-D-glucopyranoside (**25**) and kaempferol 3-*O*-(6’’-*O*-*p*-coumaroyl)-β-D-glucopyranoside (**27**).

|  | Quercetin 3-*O*-(6’’-*O*-*p*-coumaroyl)-β-D-glucopyranoside | | Kaempferol 3-*O*-(6’’-*O*-*p*-coumaroyl)-β-D-glucopyranoside | | |
| --- | --- | --- | --- | --- | --- |
| Carbon number | ^1^H | ^13^C* | ^1^H | ^13^C* | HMBC |
| 2 | - | - | - | 156.96 | - |
| 3 | - | - | - | - | - |
| 4 | - | - | - | - | - |
| 5 | - | - | - | 161.69 | - |
| 6 | 6.14 d (*J*= 2.1 Hz) | 99.19 | 6.14 d (*J*= 2.1 Hz) | 99.30 | C8, C10, C5 |
| 7 | - | - | - | 164.56 | - |
| 8 | 6.38 d (*J*= 2.1 Hz) | 93.90 | 6.40 d (*J*= 2.1 Hz) | 94.19 | C10, C9, C7, C6 |
| 9 | - | - | - | 156.55 | - |
| 10 | - | - | - | 104.20 | - |
| 1' | - | - | - | 125.58 | - |
| 2' | 7.52 d (*J*= 2.3 Hz) | 116.42 | 8.05 d (*J*= 8.4 Hz) | 131.47 | C2, C4', C6'or 2' |
| 3' | - | - | 6.78 d (*J*= 8.4 Hz) | 116.35 | C1', C5' or 3' |
| 4' | - | - | - | 160.38 | - |
| 5' | 6.78 d (*J*= 8.4 Hz) | 116.01 | 6.78 d (*J*= 8.4 Hz) | 116.35 |  |
| 6' | 7.66 dd (*J*= 8.4 Hz and 2.3 Hz) | 122.38 | 8.05 d (*J*= 8.4 Hz) | 131.47 |  |
| 1'' | 5.42 d (*J*= 7.8 Hz) | 102.03 | 5.41 d (*J*= 7.7 Hz) | 102.04 | - |
| 2'' | - | - | 3.51 - 3.70 | 71.47 | C1 |
| 3'' | - | - |  | 70.32 | - |
| 4'' | - | - |  | 73.40 | C5''ou C3'', C1'', C6''or C2'' |
| 5'' | - | - |  | 69.05 | - |
| 6'' | 4.12 dd (*J*= 11.5 Hz and 4.6Hz) | 63.62 | 4.10 d (*J*= 6.2 Hz) | 63.71 | C9''', C4'', C5''or C3'' |
| 1''' | - | - | - | 121.33 or 125.05 | - |
| 2''',6''' | 7.37 d (*J*= 8.4 Hz) | 130.62 | 7.35 d (*J*= 8.7 Hz) | 130.66 | C7''', C4''' |
| 3''',5''' | 6.83 d (*J*= 8.4 Hz) | 115.61 | 6.86 d (*J*= 8.7 Hz) | 115.52 | C1''', C5''or C3'', C4''' |
| 4''' | - | - | - | 160.53 | - |
| 7''' | 7.36 d (*J*= 15.7 Hz) | 145.31 | 7.34 d (*J*= 15.9 Hz) | 145.24 | C9''', C2'''or C6''' |
| 8''' | 6.13 d (*J*= 15.7 Hz) | 114.10 | 6.11 d (*J*= 15.9 Hz) | 114.29 | - |
| 9''' | - | - | - | 166.85 | - |
| OH-C5 | 12.64 s | - | 12.59 s | - | - |
| OH-C7 | 10.84 s | - | 10.87 s | - | C7, C6, C8 |
| OH-C3' | 9.16 s | - |  | - | - |
| OH-C4' | 9.73 s | - | 10.03 s | - | - |
| OH-C4''' | 10.01 s | - | 10.20 s | - | - |

*Chemical shifts of ^13^C NMR were obtained by correlations in HSQC.

Table S4. NMR data of ^1^H and ^13^C for Kaempferol (29).

| Carbon number | ^1^H | ^13^C |
| --- | --- | --- |
| 2 | - | 147.27 |
| 3 | - | 136.11 |
| 4 | - | 176.36 |
| 5 | - | 161.15 |
| 6 | 6.21 d (*J*= 2.0 Hz) | 96.68 |
| 7 | - | 164.38 |
| 8 | 6.46 d (*J*= 2.0 Hz) | 93.95 |
| 9 | - | 156.63 |
| 10 | - | 103.49 |
| 1' | - | 122.12 |
| 2',6' | 8.05 d (*J*= 8.9 Hz) | 129.95 |
| 4' |  | 159.67 |
| 3',5' | 6.94 d (*J*= 8.9 Hz) | 115.91 |
| OH-C3 | 9.38 s |  |
| OH-C5 | 12.48 s | - |
| OH-C7 | 10.83 s | - |
| OH-C4' | 10.13 s | - |
